# Evolutionary histories determine DNA barcoding success in vascular plants: seven case studies using intraspecific broad sampling of closely related species

**DOI:** 10.1186/s12862-016-0678-0

**Published:** 2016-05-13

**Authors:** Sofia Caetano Wyler, Yamama Naciri

**Affiliations:** Laboratoire de Systématique Végétale et Biodiversité, Conservatoire et Jardin botaniques & University of Geneva, Chemin de l’Impératrice, 1, 1292 Chambésy Geneva, Switzerland; SwissBOL, University of Geneva, Department of Genetics and Evolution, Quai Ernest Ansermet 30, 1211 Geneva, Switzerland

**Keywords:** *Acer*, *Adenostyles*, Chloroplast capture, Incomplete lineage sorting, Interspecific hybridization, *Gentiana*, *Geranium*, *Lonicera*, *Salix*, *Veronica*

## Abstract

**Background:**

Four plastid regions, *rpo*B, *rpo*C1, *mat*K, and *trn*H-*psb*A, have been recommended as DNA barcodes for plants. Their success in delimiting species boundaries depends on the existence of a clear-cut difference between inter- and intraspecific variability. We tested the ability of these regions to discriminate among closely related species in seven genera of flowering plants with different generation times (trees, perennials, and annuals). To ensure a maximum coverage of intraspecific diversity, and therefore to better evaluate the resolution power of each barcode, we applied a population genetics approach by sampling three to 45 individuals per species over a wide geographical range.

**Results:**

All possible combinations between loci were analysed, which showed that using more than one locus does not always improve the resolution power. The *trn*H-*psb*A locus was most effective at discriminating among closely related species (*Acer*, *Lonicera*, *Geranium*, and *Veronica*), singly or in combination. For *Salix*, *Adenostyles,* and *Gentiana*, the best results were obtained with the combination of *mat*K, *rpo*B, and *trn*H-*psb*A. No barcoding gap was found within six genera analysed, excepting *Lonicera*. This is due to shared polymorphisms among species, combined with very divergent sequences within species. These genetic patterns reflect incomplete lineage sorting and hybridization events followed by chloroplast capture.

**Conclusions:**

Our results strongly suggest that adding *trn*H-*psb*A to the two obligate DNA barcodes proposed by the CBOL plant-working group (*mat*K and *rbc*L) should be mandatory for closely related species. In our sampling, generation time had no influence on DNA barcoding success, as the best and worst identification successes were found for the two tree genera (*Acer*, 64 % success and *Salix*, 86 % failure). Evolutionary histories are the main factor influencing DNA barcoding success in the studied genera.

**Electronic supplementary material:**

The online version of this article (doi:10.1186/s12862-016-0678-0) contains supplementary material, which is available to authorized users.

## Background

DNA barcoding uses a short DNA sequence from a standard locus to identify the species to which a particular specimen belongs [1]. Since DNA barcoding was first used in plants, several regions have been recommended as universal barcodes [2–7]. Primarily located in the chloroplast genome, these regions focus on coding and non-coding loci. Kress and Erickson [2] proposed the combined use of *rbc*L and *trn*H-*psb*A, but other combinations have been suggested as well ([8–10]; among others). More recently, the Plant Working Group of the Consortium for the Barcode of Life adopted *rbc*L and *mat*K as the core DNA barcodes for plants [11], with *trn*H-*psb*A as an additional marker. Other studies have suggested the use of the nuclear ribosomal locus ITS [4, 12, 13], but the aim of the present study was to test for the accuracy of the chloroplast barcodes *per se* and we therefore selected *mat*K, *rpo*C1, *rpo*B, and *trn*H-*psb*A. The barcode studies published so far agree that *mat*K and *trn*H-*psb*A are the two most promising chloroplast regions for discriminating among closely related species, whereas other regions, such as *rbc*L, are more suitable for identifications at the family and/or the genus level [14]. This is the main reason why we decided to discard *rbc*L, although it is one of the official barcodes.

Because the debate has long focused on which marker(s) should be used to obtain the best assignment to species [2, 4, 7, 10, 11, 13–20], other fundamental issues have received less attention, although they are of high relevance for barcoding success. One of these issues is how many individuals should be analysed within a species to generate a reliable reference for an accurate identification. Early studies that analysed the success of DNA barcoding [2, 14, 16] did not use the closest species when pairs of species were selected. Accordingly, a higher identification success was usually obtained when barcoding floras, for which closely related taxa are not always included, versus taxonomical groups for which it is usually the case [7, 8]. Meyer and Paulay [21] raised the sample size concern but it has rarely been addressed in barcoding studies (but see [7, 22]), although it is particularly critical when working with closely related species, for which intra- and interspecific genetic variation may overlap quite frequently. Therefore the methods by which intraspecific variability is documented has a direct influence on the accuracy with which a given DNA sequence identifies species.

DNA barcoding success depends on the existence of a clear cut-off between intraspecific variation and interspecific divergence, the so-called “barcoding gap”. The barcoding gap is largely dependent on the studied groups and species, which constitutes a second issue that has hardly ever been addressed (but see [7]). Many plant species evolved recently through adaptive radiations and rapid speciation [3, 23–26]. Recent speciation with consecutive incomplete lineage sorting often results in reduced sequence divergence between the newly speciating taxa [27–29]. In the worst case, i.e. retention of ancestral polymorphism(s) among species, the identification of specimens is impossible [30]. Problematic identification of specimens also arises from hybridization between species, which is very frequent [31, 32], and polyploidization [29, 33, 34]. Therefore, the success of DNA barcoding is expected to vary among groups depending on their evolutionary history.

Still, a general prediction about DNA barcoding success can be made based on life traits such as the generation time. The short generation times that characterize annual plants are expected to lead to a rapid accumulation of mutations and to prompt species differentiation. Significant barcoding gaps are expected for such plants, leading to high DNA barcoding assignment success. The longer life spans and slower accumulation of mutations in woody plants are expected to result in poorer species delimitations [35, 36].

In this study, we analysed the impact of generation times and large sample sizes on DNA barcoding success. We addressed this question using four chloroplast loci (*mat*K, *rpo*B, *rpo*C1, and *trn*H-*psb*A) that have been proposed as barcodes [14]. These markers were evaluated for closely related species within seven genera that display different generation times: *Acer* and *Salix* (trees); *Adenostyles*, *Gentiana*, and *Lonicera* (perennials); and *Geranium* and *Veronica* (annuals). Within genera, we selected species that have clear taxonomical status with overlapping geographical distributions. We then sampled as many populations as possible in order to assess intraspecific and interspecific variation in the barcoding loci to infer how well specimens could be assigned to species with the selected chloroplast barcodes.

## Results and discussion

### Sampling

A total of 485 individuals were sampled for the 27 species used in this study (Additional file 1). Differences in sampling sizes per genus are explained by the relative abundance of some species (*Acer*—103 individuals) compared to others (*Geranium*—16 individuals) and by the effort put into sampling *Gentiana* (137 individuals) for a detailed study on the phylogeography of the Ciminalis group [37]. Samples were collected in Austria, the Czech Republic, France, Italy, Norway, Portugal, Switzerland, the United Kingdom, Spain, and Sweden from 37.05° to 69.30° in latitude and from −8.38° to 22.48° in longitude.

### Primer universality and amplification success

A DNA barcode must fulfil several requirements and should optimally be universal (present in all taxa), easily amplified (i.e., without species-specific PCR primers), short enough (so that it can be easily sequenced, even on degraded samples), informative at the species level (with enough variation insuring a satisfactory identification of species), and conserved or slightly polymorphic at the intraspecific level (so that a barcode gap can be observed).

Four candidate chloroplast regions were targeted in the present study: *mat*K, *rpo*C1, *rpo*B, and *trn*H-*psb*A. Only 440 specimens were amplified and sequenced successfully for the four loci (91 %). Loci were sequenced with 100 % success, except for *rpo*C1 and *rpo*B in one individual of *Gentiana*, and *mat*K in *Acer*, *Gentiana*, *Lonicera*, and *Veronica* (Table 1). We used four combinations of five *mat*K primers (one of them newly designed in this study) to improve the results (Additional file 2). Still, we were not able to obtain *mat*K sequences from 13 individuals of *Veronica hederifolia* (sequencing success: 58.6 %; Table 1). This marker is known to have a lower success rate of PCR amplification and sequencing [11, 13] and our results emphasize the lack of primer universality for this DNA barcode, even at the genus level (*Acer* and *Veronica*; Additional file 2). Moreover, generating fully bidirectional sequences for *matK* was sometimes challenging, a problem that has also been reported in many families, including Asteraceae [27] and Lemnaceae [10].

**Table 1:** Diversity measures for *mat*K, *rpo*C1, *rpo*B, and *trn*H-*psb*A, given separately for the seven genera (n is the number of sampled individuals)

		*mat*K	*rpo*C1	*rpo*B	*trn*H-*psb*A
*Acer* (*n* = 103)	Aligned length (bp)	849	508	349	512
	Sequencing success (%)	95.1	100	100	100
	Conserved sites (%)	98	99.2	99.1	93.8
	Parsimony informative sites (%)	1.3	0.8	0.9	3.7
*Salix* (*n* = 69)	Aligned length (bp)	855	508	349	325
	Sequencing success (%)	100	100	100	100
	Conserved sites (%)	96.4	100	99.7	91.4
	Parsimony informative sites (%)	0	0	0.3	0.9
*Adenostyles* (*n* = 37)	Aligned length (bp)	798	508	349	508
	Sequencing success (%)	100	100	100	100
	Conserved sites (%)	98.2	99.8	100	99
	Parsimony informative sites (%)	0.1	0.2	0	0.8
*Gentiana* (*n* = 135)	Aligned length (bp)	761	508	349	460
	Sequencing success (%)	98.5	99.3	99.3	100
	Conserved sites (%)	92.6	96.7	98.3	80
	Parsimony informative sites (%)	6.6	1.6	1.7	19.6
*Lonicera* (*n* = 67)	Aligned length (bp)	1190	508	340	525
	Sequencing success (%)	85.1	100	100	100
	Conserved sites (%)	96.7	99.8	98.8	97.1
	Parsimony informative sites (%)	1.7	0.2	1.2	2.7
*Geranium* (*n* = 16)	Aligned length (bp)	769	508	349	356
	Sequencing success (%)	87.5	100	100	100
	Conserved sites (%)	94.1	96.7	99.4	89
	Parsimony informative sites (%)	5.6	1.6	0	2.5
*Veronica* (*n* = 58)	Aligned length (bp)	1228	508	349	393
	Sequencing success (%)	58.6	100	100	100
	Conserved sites (%)	91.5	94.5	94.3	81.7
	Parsimony informative sites (%)	5.7	5.3	5.2	15.5

Total aligned sequence length (bp), percentage of individuals successfully amplified and sequenced, percentage of conserved and parsimony informative characters in the aligned sequences

### Sequence variation and discriminating power

Alignments, sequence variation analyses, and identification of unique sequences were performed within each genus separately. The alignment lengths for *rpo*C1 and *rpo*B were conserved for all genera, while those of *mat*K and *trn*H-*psb*A ranged from 761 to 1228 bp and from 325 to 525 bp, respectively (Table 1). For the *trn*H-*psb*A spacer, the differences in length are not surprising and are easily explained by a high number of insertion/deletion events. The use of different primer pairs for different genera explains the range in *mat*K product size.

Sequence variation was quantified using the number of conserved and parsimony informative sites. The percentage of conserved sites was high for each genus, ranging from 80 % in *Gentiana* for *trn*H-*psb*A to 100 % in *Salix* and *Adenostyles* for *rpo*C1 and *rpo*B, respectively (Table 1). The percentage of congeneric species resolved as monophyletic was accordingly very low for *rpo*C1 and *rpo*B. This is not a surprising result given the slow evolutionary rate of these two coding regions. These loci are therefore not suitable to distinguish closely related species, as also reported in other studies (e.g., [10]). We highlight that both loci have slightly lower resolution powers compared to that of the recommended DNA barcode *rbc*L [11]. Therefore the use of the latter region would not have dramatically changed our results in the present study.

The percentage of parsimony informative sites was low for most markers in all genera, especially *rpo*C1 and *rpo*B (mean = 1.4 and 1.3 %, respectively). The locus *trn*H-*psb*A harbours the highest percentage of parsimony informative sites, except in *Geranium*, for which the highest value is found with *mat*K (5.6 % instead of 2.5 % with *trn*H-*psb*A).

When considered separately, the locus with the highest number of sequences private to a single species was found with *trn*H-*psb*A (Table 2). Accordingly, the highest identification success at the species level was also observed using this locus. The ability of *trn*H-*psb*A to distinguish species is generally well accepted [16]. Many studies have recommended using this marker as a DNA barcode on a regular basis [2, 38–40]. Moreover, its use in intraspecific population studies [41, 42] highlights its utility for discriminating closely related species, which agrees with the results obtained here. Intergenic spacers are generally difficult to align across genera [43], but performing the analyses independently within each genus can surpass this obstacle.

**Table 2:**
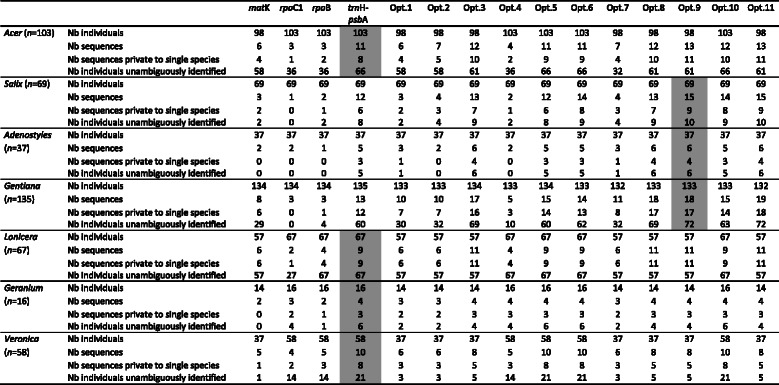
Assignment success for *mat*K, *rpo*C1, *rpo*B, and *trn*H-*psb*A given alone and arranged according to all possible combinations

Opt. for Option. Option1: *mat*K + *rpo*C1; Option2: *mat*K + *rpo*B; Option3: *mat*K+ *trn*H-*psb*A; Option4: *rpo*C1 + *rpo*B; Option5: *rpo*C1 + *trn*H-*psb*A; Option6: *rpo*B + *trn*H-*psb*A; Option7: *mat*K + *rpo*C1 + *rpo*B; Option8: *mat*K + *rpo*C1 + *trn*H-*psb*A; Option9: *mat*K + *rpo*B + *trn*H-*psb*A; Option10: *rpo*C1 + *rpo*B + *trn*H-*psb*A; Option11: *mat*K + *rpo*C1 + *rpo*B + *trn*H-*psb*A. Number of individuals successfully amplified and sequenced, number of total different sequences within the genus and the ones that are private to a single species, and number of individuals harbouring species’ private sequences. The statistics are given for the seven genera separately (n is the number of sampled individuals). The best DNA barcode(s) are highlighted in grey for each genus

### Monophyly tested by phylogenetic trees

For each barcode, we estimated the recovered species monophyly using multiple individuals per species and phylogenetic NJ trees (Additional file 3). It should be noted that the main purpose of the trees was not to study evolutionary relationships, but rather species identification.

The 103 *Acer* individuals were divided into three clades for *mat*K, *rpo*C1, and *trn*H-*psb*A: 1, *A. campestre* L. and *A. platanoides* L., 2, *A. opalus* Mill. and *A. monspessulanum* L*.*, and 3, *A. pseudoplatanus* Falk. With *rpo*B, *A. campestre*, *A. platanoides*, *A. opalus*, and *A. monspessulanum* grouped together in a single clade. *Adenostyles* species did not cluster into distinguishable clades with the four markers. For *Gentiana*, the four loci separated the four species into two main clades: 1, *G. alpina* Vill. and *G. clusii* E.P.Perrier & Songeon, and 2, *G. acaulis* L. and *G. angustifolia* Vill. Still, three *G. alpina* individuals were clustered in the second clade. For *Geranium*, *rpo*C1 and *trn*H-*psb*A were the only markers able to distinguish *G. columbinum* L. from the other two species that clustered together. For *Lonicera*, only *rpo*C1 failed to distinguish the four species into monophyletic clades (*L. caerulea* L., *L. nigra* L., and *L. alpigena* L. clustered in a single clade). *Salix* species were indiscernible with the four DNA barcodes. *Veronica hederifolia* L. individuals formed a monophyletic clade with three loci (*mat*K failed to amplify this species). With *mat*K, two clades could be observed, the first one comprising almost all *V. arvensis* L. individuals and the second one grouping *V. persica* Poir. and *V. polita* Fr. together. The four loci also agreed in clustering two *V. polita* individuals within the *V. arvensis* clade and two *V. arvensis* individuals in the *persica*-*polita* clade (Additional file 3).

Therefore, monophyletic clades grouping conspecific individuals were only observed in *Lonicera* with *mat*K, *rpo*B, and *trn*H-*psb*A. For the six remaining genera, none of the chloroplast regions was successful in reconstructing monophyletic species clades.

### Locus combination and barcode gaps

Combining markers improves the rate of correct species identification [20, 27]. In the present study, all possible combinations between loci were analysed and are reported in Table 2. Our results clearly showed that combining loci is not always an advantage. For instance, option 11, which combines all four loci, did not result in the highest identification rate, as one might expect if each locus was informative. The highest success in discriminating closely related species was always attained with a combination involving *trn*H-*psb*A. We stress, however, that it is not always the same combination of loci that gave the best results. With two loci (options 1 to 6), option 6 (*rpo*B + *trn*H-*psb*A) performed well for most genera in terms of private intraspecific diversity and number of individuals unambiguously identified. The exceptions were *Salix*, *Adenostyles*, and *Gentiana*, for whom identical or better results were obtained with option 3 (*mat*K + *trn*H-*psb*A). For the combinations with three loci (options 7–10), the same pattern was observed: whenever the number of individuals sequenced was the same among options, the combination of *mat*K and *trn*H-*psb*A performed slightly better in discriminating species. The barcoding success was enhanced when these two loci were combined, but the lower sequencing success of *mat*K limited its utility in this dataset.

None of the loci or combinations of loci performed equally for the seven genera in terms of sequencing and identification successes and no locus or combination of loci proved to be ideal for DNA barcoding. We selected *trn*H-*psb*A alone as the DNA barcode for *Acer*, *Lonicera*, *Geranium*, and *Veronica*, as the addition of other loci did not improve discrimination of species in these four genera. This is in line with the original concept of DNA barcoding, which advocates the use of a single sequence. For *Adenostyles*, combining *mat*K and *trn*H-*psb*A (option 3) performed equally or better than other options while minimizing the number of loci involved. For *Salix* and *Gentiana*, option 9, which combined *mat*K, *rpo*B, and *trn*H-*psb*A, gave the best discriminatory results.

**Fig. 1:**
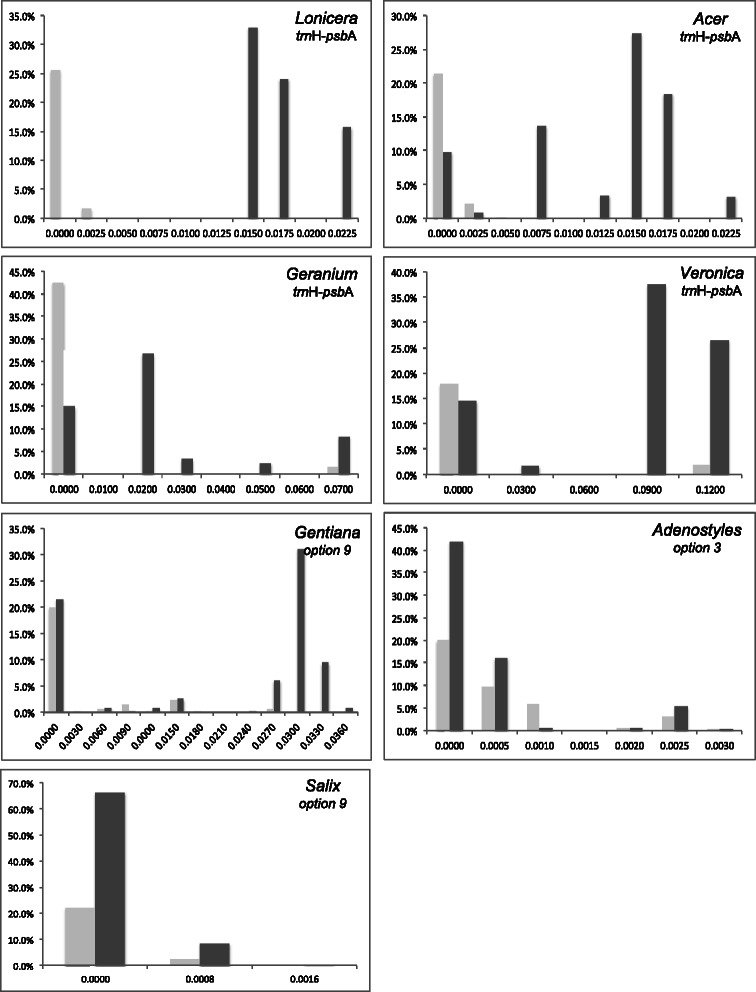
Relative distribution of intra- (light grey) and interspecific (dark grey) divergence, as measured by the K2P distance, of the defined DNA barcode alone or combined within each genus

Barcoding gaps were evaluated by comparing the intra- and interspecific divergences within each genus [21]. The Kimura 2-parameter (K2P) distances were computed for the chosen locus/combination according to the above chosen options: *trn*H-*psb*A for *Acer*, *Lonicera*, *Geranium*, and *Veronica*, option 3 for *Adenostyles*, and option 9 for *Salix* and *Gentiana. Lonicera* was the only genus with a clear barcoding gap (Fig. 1). The expected cut-off between intra- and interspecific K2P distances was not observed in all other genera. *Acer*, *Geranium*, *Veronica*, and *Gentiana* also tend to have higher inter- than intraspecific distances though there is some overlap at frequencies ranging between 9 and 20 %. Conversely, intra- and interspecific distances overlap completely in *Veronica* and *Geranium*.

**Fig. 2:**
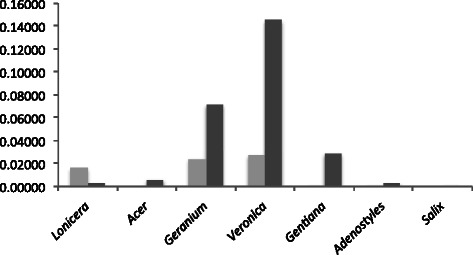
Minimum average interspecific distance (light grey) against the maximum intraspecific divergence (dark grey), as measured by the K2P distance, of the defined DNA barcode within each genus

Analyses were performed separately in each genus, so overlaps between intra- and interspecific variation are expected when closely related taxa are included. In our study, the overlap between the two distributions indicated that DNA barcoding with the studied chloroplast loci is not effective for the studied genera, except *Lonicera*. Indeed, the nearest-neighbour distance (minimum average interspecific distance) was, with the exception of *Lonicera*, lower than the maximum intraspecific distance (Fig. 2). This type of result is associated to two main population genetic factors, incomplete lineage sorting and interspecific hybridization [21, 28, 44]. Recently diverged species are likely to have a null or very low average sequence distance to the most closely related species. Moreover, hybridization events associated with chloroplast captures tend to maximize the intraspecific divergence, as divergent chloroplasts can be exchanged and shared among species [28]. This seems to be the case in *Geranium*, *Gentiana*, and *Veronica*.

### Influence of sampling size

The extent to which large sample sizes influenced the capture of intraspecific variability was analysed. The correlation between sampling sizes and number of unique sequences was only found for *Adenostyles* (*r* = 0.99, *n* = 3, *P* < 0.05). The lack of correlation was observed for the majority of the genera, within genera (*n* = 3—5) and overall (*r* = 0.21, *n* = 27, *P* > 0.31). We employed the rarefaction method to quantify the average number of different sequences that would be recovered using a small sampling size within species. For a sampling size of three individuals, the sequence richness (*Rs*) ranged between one for species with no intraspecific diversity for the studied loci (*Acer monspessulanum, A. platanoides, Adenostyles leucophylla* DC.*, Gentiana acaulis, Geranium columbinum, Lonicera nigra*, and *Veronica persica*) and 2.5 (*Gentiana clusii*, *Salix herbacea* Schrenk, and *S. reticulata* L.). Interestingly, the most variable species never reached *Rs* = 3, despite having six to nine sequences. Similarly, other species that displayed moderate variation (two to three unique sequences) had very low *Rs* values (*Acer pseudoplatanus Rs* = 1.2 and *Gentiana angustifolia Rs* = 1.1). *Rs* was calculated using observed sequence frequencies, emphasizing the fact that small samples will often miss rare sequences.

### Median joining networks and life histories

**Fig. 3:**
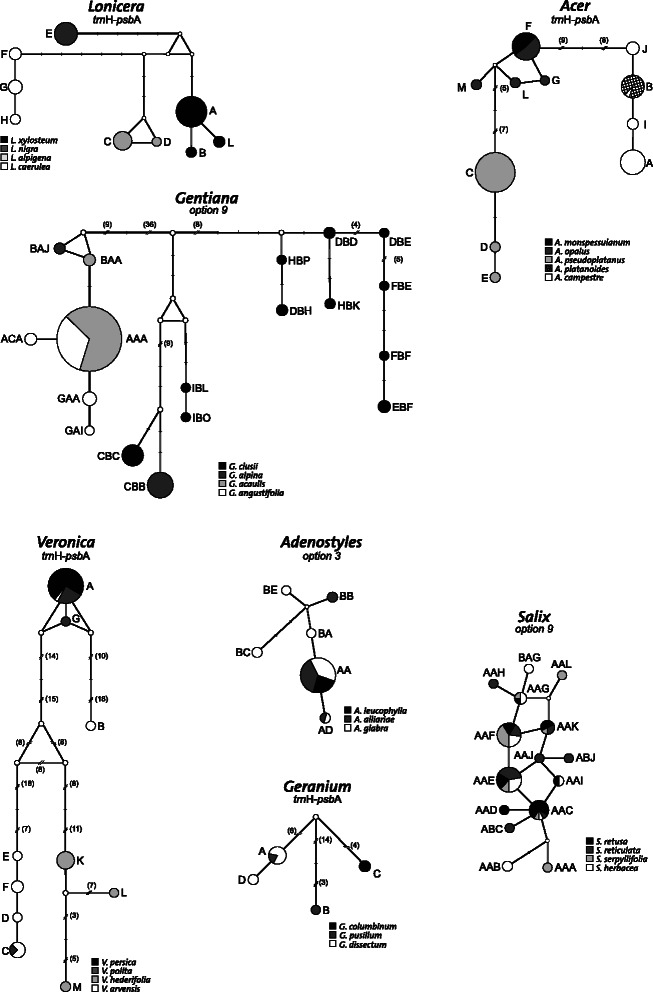
Sequence networks drawn for selected DNA barcode(s) within each genus. Each unique sequence combination is represented by a circle, with size proportional to the number of individuals sharing the sequence. Each branch segment represents a single mutation; substitutions are coded as full lines and indels as double lines

Median joining networks were drawn with the selected barcode for each genus separately (Fig. 3) and illustrate why barcoding gaps were seldom observed. Sister species shared the same sequences in six out of the seven genera. *Lonicera* was the only genus for which complete lineage sorting was observed. According to the most recent phylogeny of the genus, the four species analysed here belong to separate subclades of the *Lonicera* clade [45]. However, these four sections were poorly supported, so it would be interesting to analyse the DNA barcoding performance if one had considered species from the same subclade.

Among the other genera, three different processes can explain the sharing of chloroplast sequences between species. The first is incomplete lineage sorting among sister species, which is observed in four genera. 1) Within *Acer* this pattern occurs twice: between *A. campestre* and *A. platanoides* (sequence B) and between *A. monspessulanum* and *A. opalus* (sequence F). The latest *Acer* phylogeny [46] confirms that these two pairs are sister species. 2) Within *Gentiana*, the AAA sequences are shared between *G. angustifolia* and *G. acaulis*. According to Christe et al. [37], who studied the phylogeographic patterns within the Ciminalis group, these species have diverged recently. 3) Within *Veronica*, *V. persica* and *V. polita* share sequence A. The latest phylogeny, based on ITS, reported that these are sister species within subgenus *Pocilla* [47]. 4) *Adenostyles alliariae* Kern and *A. leucophylla* share sequence A, which reflects their status as sister species [48].

The second process that explains sequence sharing is putative hybridization between species. This is observed within both genera of annual plants: *Veronica polita* is characterized by sequences A, G, and C. Sequence C, which is distinct by 66 mutations from the two others, is shared with *V. arvensis*. Hybridization is recognized as an important evolutionary force for some subgenera of *Veronica* [9]. In published phylogenies, the species for which hybridization is suspected are grouped together in the ITS consensus tree and the cladogram based on the ITS sequences, chromosome numbers, and iridoid composition [47, 49]. *Geranium pusillum* L. harbours two different sequences that are separated by 23 mutations; one is shared with *G. dissectum* L. (sequence A). A third case of hybridization was also observed within *Gentiana*. Indeed, *G. alpina* possesses two sequences that are distinct at 63 positions, one of which (BAJ) is closely related to the most frequent sequence (CBC) in *G. clusii*. Hybridizations between *Gentiana* species have often been reported [50–52], and distinct events of chloroplast capture involving these species have also been suggested [37].

The complete lack of structure observed within *Salix* was surprising, but not new. The three most common sequences were shared among the four species analysed in this study, and only 14.5 % of the specimens had private sequences. Our results agreed with a recent study that documented little variation in chloroplast loci among *Salix* species, with most taxa sharing the same barcode sequence. Complex processes involving “recent repeated plastid capture events, aided by widespread hybridization and long-range seed dispersal, but primarily propelled by one or more trans-species selective sweeps” were suggested to explain the observed pattern [53].

In summary, our results illustrate the effect of species’ evolutionary histories on DNA barcoding success. In this study, evolutionary history refers to recent speciation events with incomplete lineage sorting and retention of ancestral sequences, interspecific hybridization events with chloroplast capture, and spatial expansions with sequence surfing [54]. It is commonly acknowledged that several processes underlying the evolutionary patterns in plants cause a partial failure of DNA barcodes to track species boundaries [13, 28, 29, 43], but this study shows that the absence of a barcoding gap among closely related species is quite common, with extensive sharing of diversity among species (49 %).

## Conclusions

The main factor that impacts DNA barcoding success is a species’ evolutionary history. Sampling many specimens from a wide geographical distribution within species was shown to be important as it increases the likelihood of capturing the intraspecific genetic variation. However, sampling sizes were not correlated to the number of different sequences found within a species, because variability is mostly influenced by the species’ evolutionary history. Our study shows that within the same genus, and even within the same section, sequence variation can range from low to high, depending on the species (for instance, *Gentiana clusii* and *G. acaulis* – 11 and 2 different sequences, respectively, with similar sampling sizes collected from the whole distribution range). Such diverse patterns were obtained through different demographic regimes (bottlenecks, spatial or demographic expansions) that shaped the diversity and its structuring.

Life traits, such as generation time, do not influence the DNA barcode success in our study. The best and worst identification successes were indeed found for the two tree genera (*Acer*, 64 % success and *Salix*, 86 % failure). The annual plants analysed here showed, on average, a higher number of mutations between sequences than was observed in perennials. This should, theoretically, be an advantage for DNA barcoding success, but the incidence of interspecific hybridization within these genera highly shapes the observed genetic pattern and results in specimen identification failures. Therefore, our results underline the impact of species’ evolutionary histories on the ability to successfully identify a given specimen.

We found that the most useful combination of loci for discriminating closely related species can differ from one genus to another, and this agrees with other papers that discussed the interest of different loci as DNA barcodes. However, our results demonstrated that *trn*H-*psb*A is almost always the best DNA barcode locus. This supports the proposal for *trn*H-*psb*A to be added to the two core DNA chloroplast barcodes proposed by the CBOL plant working group. Moreover, our results show that the K2P metric is not the most appropriate, as it does not take into account invertion/deletion events that are of high interest, especially for *trn*H-*psb*A, to distinguish and document sequence variation.

## Methods

### Sampling strategy

Genera and species were selected for the present study based on the following criteria: generation times, geographic distribution ranges, clear taxonomical status, and ease of recognition. In each case, all possible closely related species were sampled except any rare or endangered ones. Species of two genera are trees (*Acer* and *Salix*), three genera include perennial herbaceous or woody species (*Adenostyles*, *Gentiana*, and *Lonicera*), whereas two genera include annual species (*Geranium* and *Veronica*). For each species, as many localities as possible were sampled, over the largest possible geographical range, to gather as much intraspecific variation as possible (Additional file 1). For each individual, an herbarium voucher was collected, identified by an expert, and deposited at the Geneva herbarium (G). For protected *Gentiana* species, high-quality photos were taken in lieu of herbarium specimens.

### DNA extraction, amplification and sequencing

Total genomic DNA was extracted using the NucleoSpin© Plant II kit (Macherey-Nagel, GmbH & Co. KG, Düren, Germany) following the supplier’s instructions. Three cpDNA coding regions (*mat*K, *rpo*C1, and *rpo*B) and one cpDNA spacer (*trn*H-*psb*A) were amplified and sequenced. PCR was performed in 20 μL total volume with 0.60 U Taq (Roche, Mannheim, Germany), 2 μL of 10X buffer containing 20 mM MgCl_2_, 0.8 μL of each primer (10 mM), 0.4 μl of a mix containing 10 mM of each dNTP (Roche), and 0.85 μL of template DNA of unknown concentration. The PCR program had an initial heating step at 95 °C for 6 min, followed by 35 cycles of denaturation at 95 °C for 30 s, annealing for 30 s at a locus-specific temperature, elongation at 72 °C for 45 s, and a final elongation step at 72 °C for 10 min. Annealing temperatures varied between 45 and 52 °C depending on locus and species (see Additional file 2 for details). The primers used are also listed in Additional file 2. PCR products were cleaned and bidirectionally sequenced using the PCR primers on an ABI 377 automated sequencer (Applied Biosystems, Foster City, CA, USA) following the manufacturer’s protocols.

### Sequence alignment and data analyses

Contig assembly and sequence consensus were generated using Sequencher (GeneCodes Corporation, Ann Arbor, Michigan, USA). Barcode sequences were aligned in BIOEDIT 7.0.3.5 [55] and edited manually. Sequence variation was then characterized using the percentage of conserved sites, the percentage of parsimony informative sites, and the number of unique sequences per species. This last measure is the only one that takes into account insertion/deletion and inversion events. Both events were manually coded as single mutation steps [56]. Sequence variation analyses were then performed in MEGA version 6 [57]. All sequences were deposited in GenBank under accession numbers KU672731—KU674305 and KU672731—KU674305 (Additional file 4).

In order to investigate how well the different markers performed individually in identifying species within a genus, the number of sequences that were private to a single species was checked and the number of individuals unambiguously identified was reported. We also performed a comparison of all possible locus-combinations.

Species discrimination was evaluated using tree-based analyses. The Neighbour-Joining tree reconstruction recommended as the standard barcoding method [1] was adopted and performed with SeaView 4.4.0, based on the K2P model and 100 replicates for bootstrap analyses [58].

The presence of barcoding gaps was analysed by graphing the distributions of intra- and interspecific genetic distances for each genus. Sequence divergences were calculated using pairwise distances with the Kimura 2-parameter in MEGA [57].

The correlation between sampling size and the number of unique sequences was computed overall species and within genera for *trn*H-*psb*A, which was the most diverse barcode within species and the only one common to all genera. The sequence richness (*Rs*) was computed for a sample size of three individuals, using the rarefaction methods that takes into account sequence frequencies in each species [59]. *Rs* was used to quantify the average number of different sequences that would be recovered using a sampling size of three individuals within species. Correlations and *Rs* were computed in Excel and confidence intervals for correlation coefficients were assessed in the online program VassarStat (http://vassarstats.net/) using the Fisher r-to-z transformation.

Median joining networks of the sequences were drawn using the program Network [60]. These analyses were performed, within each genus, on the defined DNA barcode alone or combined: *trn*H-*psb*A alone for *Acer*, *Lonicera*, *Geranium*, and *Veronica*; option 3 (*mat*K and *trn*H-*psb*A) for *Adenostyles*, and option 9 (*mat*K, *rpo*B, and *trn*H-*psb*A) for *Salix* and *Gentiana*. Site mutations and indels were equally weighted and all the structural mutations (inversions and insertions/deletions of more than 1 bp) were treated as single-step events.

### Availability of data and materials

The datasets supporting the conclusions of this article are available in the Genbank repository, [accession numbers KU672731—KU674305 and KU672731—KU674305 at http://www.ncbi.nlm.nih.gov/genbank/].

## Additional files

Additional file 1: List of individuals. For each individual, the country, district, and absolute coordinates in decimal degrees are given. (PDF 29 kb) Additional file 2: Amplification conditions. For each species, annealing temperature and the primer combination are given for *mat*K, *rpo*C1, *rpo*B, and *trn*H-*psb*A. (PDF 27 kb) Additional file 3: Phylogenetic trees. For each genus and locus, a neighbour joining tree is presented. Bootstrap values above 80 % are shown above branches. Codes following species names are individual numbers (see Additional file 1). (PDF 572 kb) Additional file 4: Genbank accession numbers. For each sample, accession numbers for the four loci are given. (PDF 71 kb)
