# Supplementary material for: Evolutionary histories determine DNA barcoding success in vascular plants: seven case studies using intraspecific broad sampling of closely related species
Source: BMC Evol Biol. 2016 May 13;16:103. doi: 10.1186/s12862-016-0678-0 (PMC4866073; doi:10.1186/s12862-016-0678-0)
Supplement: Additional file 1: — List of individuals. For each individual, the country, district, and absolute coordinates in decimal degrees are given. (PDF 29 kb) [file 12862_2016_678_MOESM1_ESM.pdf]

Genus	Species	Individual	Country	District	Longitude	Latitude
	campestre	Aca_1503	Switzerland	Genève	6.1236	46.2895
		Aca_1522	France	Haute-Savoie	5.8930	46.1083
		Aca_1553	France	Savoie	5.9256	45.5132
		Aca_0033	France	Ain	5.7111	45.9809
		Aca_1577	Switzerland	Vaud	6.2320	46.4232
		Aca_1593	Switzerland	Vaud	6.4420	46.6447
		Aca_1627	France	Isère	5.6297	45.0057
		Aca_1658	France	Isère	5.8980	44.7746
		Aca_1697	France	Hautes-Alpes	5.8398	44.6397
		Aca_1762	France	Haute-Savoie	6.4196	46.0280
		Aca_1088	France	Hautes-Pyrénées	0.0312	42.8831
		Aca_1328	France	Tarn	2.0549	43.4315
		Aca_1005	Switzerland	Vaud	6.8451	46.4632
		Aca_5515	France	Hautes-Alpes	6.0528	44.5701
		Aca_5525	France	Hautes-Alpes	5.8951	44.2958
		Aca_5552	France	Alpes-de-Haute-Provence	5.9510	44.1252
		Aca_5558	France	Alpes-de-Haute-Provence	5.8059	43.8978
		Aca_5588	France	Alpes-de-Haute-Provence	6.0350	43.6945
		Aca_5626	France	Var	5.9709	43.2663
		Aca_5647	France	Var	5.9315	43.3894
		Aca_5659	France	Var	5.8188	43.3507
		Aca_5821	Czech Republic	Prague	14.4121	50.0434
		Aca_5824	Czech Republic	Prague	14.3893	50.0418
		Aca_5836	Czech Republic	Prague	14.1556	49.9634
		Aca_1038	United Kingdom	Cumbria	-3.1250	54.6775
		Aca_1025	Italy	Teramo	13.7167	42.5833
		Aca_1031	Italy	Chicti	14.1833	42.1833
	platanoides	Apl_1560	France	Savoie	5.9354	45.5304
		Apl_1575	Switzerland	Vaud	6.2320	46.4232
		Apl_1607	Switzerland	Vaud	6.6116	46.7028
		Apl_1631	France	Isère	5.6193	45.0088
		Apl_1679	France	Hautes-Alpes	5.9532	44.6729
		Apl_1870	Switzerland	Tessin	9.0577	46.2481
		Apl_1098	France	Pyrénées Atlantiques	-0.3832	42.9712
		Apl_1006	Switzerland	Vaud	6.8451	46.4632
		Apl_5088	Austria	Carinthie	12.9553	46.6365
		Apl_5518	France	Hautes-Alpes	6.0528	44.5701
		Apl_5597	France	Var	6.0295	43.4952
		Apl_5822	Czech Republic	Prague	14.4121	50.0434
		Apl_5830	Czech Republic	Prague	14.3893	50.0418
		Apl_1036	United Kingdom	Cumbria	-3.1072	54.6778
		Apl_6265	Sweden	Uppland	17.6214	59.8417
	opalus	Aop_1597	Switzerland	Vaud	6.4420	46.6447
		Aop_1624	France	Isère	5.6297	45.0057
		Aop_1629	France	Isère	5.6297	45.0057
		Aop_1655	France	Isère	5.8980	44.7746
		Aop_1694	France	Hautes-Alpes	5.8398	44.6397
		Aop_1765	Switzerland	Valais	7.4551	46.2759
		Aop_5208	France	Isère	5.7463	45.3538

	opalus	Aop_5157	France	Alpes-de-Haute-Provence	6.7688	44.4736
		Aop_5512	France	Hautes-Alpes	6.0528	44.5701
Acer		Aop_5632	France	Var	5.9633	43.2630
		Aop_5650	France	Var	5.9315	43.3894
		Aop_5661	France	Var	5.7069	43.3237
		Aop_5549	France	Alpes-de-Haute-Provence	5.9510	44.1252
		Aop_1028	France	Somme	14.1833	42.1833
		Amo_1520	France	Haute-Savoie	5.8930	46.1083
		Amo_0168	France	Hautes-Alpes	5.7939	44.2754
		Amo_5546	France	Alpes-de-Haute-Provence	5.9088	44.0758
		Amo_5585	France	Alpes-de-Haute-Provence	6.0350	43.6945
		Amo_5595	France	Var	6.0152	43.5727
	monspessulanum	Amo_5629	France	Var	5.9637	43.2639
		Amo_5644	France	Var	5.9315	43.3894
		Amo_5656	France	Var	5.8188	43.3507
		Amo_5667	France	Bouches-du-Rhône	5.6542	43.3417
		Amo_6102	Italy	Sardaigne	9.4333	40.2500
		Amo_6105	Italy	Sardaigne	9.3000	40.0167
		Aps_1557	France	Savoie	5.9354	45.5304
		Aps_1572	Switzerland	Vaud	6.2320	46.4232
		Aps_1605	Switzerland	Vaud	6.6116	46.7028
		Aps_1634	France	Isère	5.6193	45.0088
		Aps_1682	France	Hautes-Alpes	5.9532	44.6729
		Aps_1759	France	Haute-Savoie	6.9473	46.0046
		Aps_1805	Switzerland	Valais	8.1384	46.3887
		Aps_1853	Switzerland	Tessin	8.9110	46.5232
		Aps_1865	Switzerland	Tessin	9.0577	46.2481
		Aps_1866	Switzerland	Tessin	9.0577	46.2481
		Aps_0502	Corsica	Corse-du-Sud	9.1852	41.8733
		Aps_0509	Corsica	Corse-du-Sud	9.1497	42.0757
		Aps_1085	France	Hautes-Pyrénées	0.1010	42.9031
		Aps_1092	France	Pyrénées Atlantiques	-0.3832	42.9712
		Aps_1316	France	Hautes-Pyrénées	-0.0149	42.7375
		Aps_1022	Scotland	Highland	-4.4116	57.9724
		Aps_1008	Switzerland	Vaud	6.8451	46.4632
	pseudoplatanus	Aps_0141	Italy	Cuneo	7.0426	44.2581
		Aps_1126	Italy	Cuneo	7.0098	44.5793
		Aps_5277	Austria	Tyrol Nord	11.7643	47.4444
		Aps_5282	Austria	Tyrol Nord	12.4157	47.3692
		Aps_5294	Austria	Styrie	13.6842	47.4390
		Aps_5021	Austria	Styrie	15.6992	47.6816
		Aps_5036	Austria	Carinthie	14.5552	46.4825
		Aps_5085	Austria	Carinthie	12.9553	46.6365
		Aps_5188	France	Alpes-de-Haute-Provence	6.5919	44.2455
		Aps_5520	France	Hautes-Alpes	6.0528	44.5701
		Aps_5664	France	Var	5.7069	43.3237
		Aps_5750	Switzerland	Lucerne	47.0083	8.2742
		Aps_5789	France	Savoie	6.4648	45.2022
		Aps_5827	Czech Republic	Prague	14.3893	50.0418
		Aps_1037	United Kingdom	Cumbria	-3.1275	54.6814
		Aps_1042	United Kingdom	Cumbria	-3.3964	54.7664
		Aps_6269	Sweden	Uppland	17.6286	59.8425

		Aps_1128	Italy	Cuneo	7.0098	44.5793
		Aps_0801	Portugal	Braga	-8.3772	41.5519
		She_1747	Switzerland	Valais	6.9704	46.0273
		She_1783	Switzerland	Valais	7.5552	46.1328
		She_1885	Switzerland	Valais	8.3869	46.4779
		She_1176	Switzerland	Valais	7.1391	45.8892
		She_0553	Norvege	Sor-Trondelag	9.6167	62.2972
		She_0556	Norvege	Nordland	15.3233	66.5493
		She_0559	Norvege	Oppland	9.1581	61.3958
	herbacea	She_0562	Norvege	Hordaland	7.4931	60.3710
		She_0138	France	Alpes-de-Haute-Provence	6.9321	44.4267
		She_1111	France	Hautes-Alpes	6.9917	44.7975
		She_1133	France	Hautes-Alpes	6.9486	44.6626
		She_5109	Switzerland	Grisons	9.9766	46.7422
		She_5132	Switzerland	Valais	7.0649	46.0151
		She_5176	France	Alpes-de-Haute-Provence	6.5448	44.2429
		She_5194	France	Hautes-Alpes	6.1875	44.7238
		She_5713	France	Savoie	6.7324	44.8217
		Sri_1750	Switzerland	Valais	6.9704	46.0273
		Sri_1794	Switzerland	Valais	7.5628	46.1308
		Sri_1840	Switzerland	Tessin	8.8080	46.5618
		Sri_1173	Switzerland	Valais	7.1019	46.1815
		Sri_0544	Norvege	Sor-Trondelag	9.6167	62.2972
		Sri_0547	Norvege	Finnmark	22.4768	69.2973
		Sri_0550	Norvege	Hordaland	7.4931	60.3710
		Sri_1891	France	Pyrénées Atlantiques	-0.3264	42.9362
	reticulata	Sri_0105	France	Savoie	6.6968	45.7162
		Sri_0117	France	Savoie	6.1891	45.2179
		Sri_1343	Switzerland	Valais	7.2923	46.3563
		Sri_1123	France	Hautes-Alpes	6.9654	44.7972
		Sri_1142	France	Hautes-Alpes	6.9461	44.6609
		Sri_5077	Italy	Udine	12.9548	46.6033
		Sri_5147	France	Savoie	6.6138	45.1133
		Sri_5720	France	Savoie	6.7324	44.8217
		Sri_5763	Switzerland	Valais	6.8911	46.0539
		Sri_5783	France	Hautes-Alpes	7.0183	44.8243
Salix		Sru_1753	Switzerland	Valais	6.9704	46.0273
		Sru_1846	Switzerland	Tessin	8.8080	46.5618
		Sru_1876	Switzerland	Tessin	9.1586	46.1663
		Sru_1170	Switzerland	Valais	7.1019	46.1815
		Sru_1894	France	Pyrénées Atlantiques	-0.3264	42.9362
		Sru_0102	France	Savoie	6.6968	45.7162
		Sru_0114	France	Savoie	6.1891	45.2179
		Sru_1346	Switzerland	Valais	7.2923	46.3563
		Sru_1114	France	Hautes-Alpes	6.9917	44.7975
		Sru_1139	France	Hautes-Alpes	6.9493	44.6661
	retusa	Sru_5214	France	Isère	5.7199	45.3428
		Sru_5252	Austria	Vorarlberg	10.1588	47.1569
		Sru_5269	Austria	Northern Tirol	11.7653	47.4453
		Sru_5060	Austria	Carinthie	14.4983	46.5030
		Sru_5079	Italy	Udine	12.9548	46.6033
		Sru_5106	Switzerland	Grisons	9.9766	46.7422

		Sru_5135	Switzerland	Valais	7.0649	46.0151
		Sru_5152	France	Savoie	6.6138	45.1133
		Sru_5717	France	Savoie	6.7324	44.8217
		Sru_5757	Switzerland	Valais	6.8825	43.0522
		Sru_5786	France	Hautes-Alpes	6.7365	44.8195
		Sru_1789	Switzerland	Valais	7.5628	46.1308
	serpillifolia	Sse_1771	Switzerland	Valais	7.5628	46.1308
		Sse_1843	Switzerland	Tessin	8.8080	46.5618
		Sse_1167	Switzerland	Valais	7.0994	46.1725
		Sse_0135	Italy	Cuneo	6.9410	44.4233
		Sse_1108	France	Hautes-Alpes	6.9917	44.7975
		Sse_1136	France	Hautes-Alpes	6.9493	44.6661
		Sse_5052	Austria	Carinthie	14.4862	46.5025
		Sse_5097	Switzerland	Grisons	10.2928	46.6438
		Sse_5150	France	Savoie	6.6138	45.1133
		Sse_5723	France	Hautes-Alpes	6.7321	44.8196
		Sse_5766	Switzerland	Valais	6.8911	46.0539
		Sse_5777	France	Hautes-Alpes	7.0183	44.8243
		Sse_5780	France	Hautes-Alpes	7.0183	44.8243
Adenostyles	*alliariae*	Aal_1797	Switzerland	Valais	7.5609	46.1588
		Aal_1821	Switzerland	Valais	8.3641	46.5610
		Aal_1833	Switzerland	Grisons	8.8448	46.6338
		Aal_1882	Switzerland	Tessin	9.1223	46.1878
		Aal_0060	Italy	Bolzano	11.8099	46.5509
		Aal_1158	Switzerland	Valais	7.1070	46.0281
		Aal_1082	France	Hautes-Pyrénées	0.1839	42.9134
		Aal_1898	France	Pyrénées Atlantiques	-0.2869	42.9540
		Aal_5220	France	Isère	5.5872	45.1815
		Aal_5274	Austria	Northern Tirol	11.7653	47.4453
		Aal_5009	Austria	Styrie	14.0772	47.2924
		Aal_5027	Austria	Styrie	15.7100	47.6790
		Aal_5045	Austria	Carinthie	14.5134	46.5009
		Aal_5754	Switzerland	Valais	6.9383	46.0688
	glabra	Agl_0020	France	Drôme	5.4836	44.7623
		Agl_1703	France	Hautes-Alpes	5.9077	44.6113
		Agl_0081	Italy	Belluno	12.0830	46.5060
		Agl_1164	Switzerland	Valais	7.1040	46.1675
		Agl_0126	France	Alpes-de-Haute-Provence	6.6911	44.5337
		Agl_1340	Switzerland	Valais	7.2923	46.3563
		Agl_1117	France	Hautes-Alpes	6.9543	44.8092
		Agl_1145	France	Hautes-Alpes	6.9316	44.6666
		Agl_5211	France	Isère	NA	NA
		Agl_5012	Austria	Styrie	15.6978	47.6838
		Agl_5024	Austria	Styrie	15.6992	47.6816
		Agl_5082	Italy	Udine	12.9502	46.6000
		Agl_5166	France	Alpes-de-Haute-Provence	6.7400	44.4792
		Agl_5185	France	Alpes-de-Haute-Provence	6.5550	44.2462
		Agl_5747	Switzerland	Lucerne	8.2733	47.0077
		Agl_5706	France	Hautes-Alpes	6.7220	44.8338
		Ale_0123	France	Alpes-de-Haute-Provence	6.6916	44.5374
		Ale_0132	Italy	Cuneo	7.0861	44.2313
		Ale_0150	Italy	Aosta	7.5035	45.9394

leucophylla	Ale_1148	France	Hautes-Alpes	6.9316	44.6619
	Ale_5122	Switzerland	Valais	7.0649	46.0151
	Ale_5179	France	Alpes-de-Haute-Provence	6.5466	44.2453
	Ale_5774	France	Turin	6.9686	44.8119
	Gan_0005	France	Drôme	5.4226	44.8613
	Gan_0008	France	Drôme	5.3969	44.7898
	Gan_0011	France	Drôme	5.3014	44.8778
	Gan_0014	France	Drôme	5.4441	44.7402
	Gan_0017	France	Drôme	5.4394	44.7192
	Gan_0023	France	Drôme	5.5769	44.6887
	Gan_1538	France	Savoie	5.8639	45.4836
	Gan_1546	France	Savoie	5.9085	45.4768
	Gan_1548	France	Savoie	5.9354	45.5304
	Gan_1640	France	Isère	5.5476	44.9145
	Gan_1643	France	Isère	5.5587	44.8744
	Gan_1646	France	Isère	5.5584	44.8703
	Gan_1652	France	Isère	5.5991	44.7869
	Gan_1661	France	Hautes-Alpes	5.8910	44.7419
	Gan_1667	France	Hautes-Alpes	5.9574	44.6977
angustifolia	Gan_1691	France	Hautes-Alpes	5.8398	44.6397
	Gan_1700	France	Hautes-Alpes	5.9077	44.6113
	Gan_1720	France	Hautes-Alpes	6.0475	44.6233
	Gan_1728	France	Isère	5.7476	44.9145
	Gan_0155	France	Drôme	5.2037	44.6153
	Gan_0158	France	Drôme	5.2575	44.4878
	Gan_0170	France	Drôme	5.3869	44.4439
	Gan_5202	France	Isère	5.7463	45.3538
	Gan_5205	France	Isère	5.7229	45.3462
	Gan_5216	France	Isère	5.5862	45.1793
	Gan_5223	France	Isère	5.5926	45.1928
	Gan_5225	France	Isère	5.5758	45.2405
	Gan_5226	France	Isère	5.6124	45.1503
	Gan_5228	France	Isère	5.6133	45.0828
	Gan_5302	France	Isère	5.9758	44.8591
	Gan_0174	France	Drôme	6.6138	45.1133
	Gac_0029	France	Ain	5.7216	46.0810
	Gac_1725	France	Isère	5.7281	44.9107
	Gac_1741	Switzerland	Valais	6.9693	46.0336
	Gac_1744	Switzerland	Valais	6.9703	46.0302
	Gac_1786	Switzerland	Valais	7.5628	46.1308
	Gac_1827	Switzerland	Uri	8.4327	46.5884
	Gac_1830	Switzerland	Grisons	8.6709	46.6589
	Gac_1850	Switzerland	Tessin	8.8119	46.5596
	Gac_1856	Switzerland	Tessin	9.0633	46.2671
	Gac_1879	Switzerland	Tessin	9.1588	46.1671
	Gac_0050	Italy	Trento	11.6600	46.5003
	Gac_0063	Italy	Belluno	12.2579	46.5835
	Gac_0072	Italy	Belluno	12.2258	46.5225
	Gac_0087	France	Haute-Savoie	6.2942	45.8384
	Gac_1179	Switzerland	Valais	7.1240	45.8960
	Gac_1896	France	Pyrénées Atlantiques	-0.3264	42.9362
	Gac_1312	France	Hautes-Pyrénées	-0.0450	42.7190

		Gac_0108	France	Savoie	6.6995	45.7140
		Gac_0129	Italy	Cuneo	7.0942	44.2306
		Gac_0147	Italy	Aosta	7.5209	45.9256
		Gac_0162	France	Haute-Savoie	6.5814	46.1732
		Gac_0177	France	Drôme	5.5731	44.8122
	acaulis	Gac_5002	Austria	Styrie	13.7315	47.3621
		Gac_5004	Austria	Styrie	14.0800	47.2720
		Gac_5033	Austria	Carinthie	14.6866	46.9494
		Gac_5071	Austria	Carinthie	13.2990	46.5639
		Gac_5091	Italy	Bolzano	11.3262	46.8441
		Gac_5100	Switzerland	Grisons	10.2918	46.6431
		Gac_5103	Switzerland	Grisons	9.9841	46.7445
		Gac_5124	Switzerland	Valais	7.0625	46.0210
		Gac_5141	Switzerland	Valais	6.9386	46.0724
		Gac_5144	France	Savoie	6.6075	45.1193
		Gac_5169	France	Alpes-de-Haute-Provence	6.7351	44.4791
		Gac_5182	France	Alpes-de-Haute-Provence	6.5501	44.2452
		Gac_5233	France	Savoie	6.0408	45.6300
		Gac_5234	France	Savoie	6.0411	45.6304
Gentiana		Gac_5255	Austria	Vorarlberg	10.1571	47.1572
		Gac_5261	Austria	Northern Tirol	10.2101	47.1291
		Gac_5267	Austria	Northern Tirol	11.7642	47.4444
		Gac_5709	France	Hautes-Alpes	6.3896	45.0524
		Gac_5524	Switzerland	Vaud	7.1964	46.3243
		Gac_5313	Spain	Aragon	0.1198	42.6646
		Gac_5318	Spain	Aragon	0.6029	42.5573
		Gac_0194	Italy	Verona	10.8942	45.6950
		Gac_0200	Italy	Brescia	10.4617	45.8383
		Gal_1738	Switzerland	Valais	6.9693	46.0336
		Gal_1774	Switzerland	Valais	7.5447	46.1330
		Gal_1859	Switzerland	Tessin	9.0631	46.2733
		Gal_1873	Switzerland	Tessin	9.1586	46.1661
		Gal_1185	Switzerland	Valais	7.0858	46.1726
		Gal_1019	France	Hautes-Pyrénées	0.1348	42.9356
		Gal_1888	France	Pyrénées Atlantiques	-0.3264	42.9362
		Gal_1305	France	Hautes-Pyrénées	-0.0450	42.7190
		Gal_0111	France	Savoie	6.1880	45.2141
		Gal_0120	France	Isère	5.9083	45.1295
		Gal_5112	Spain	Granada	-3.3665	37.0530
	alpina	Gal_5115	Spain	Granada	-3.3546	37.0463
		Gal_5118	Spain	Granada	-3.3731	37.0752
		Gal_5138	Switzerland	Valais	6.9386	46.0724
		Gal_5143	France	Savoie	6.6138	45.1133
		Gal_5172	France	Alpes-de-Haute-Provence	6.5448	44.2429
		Gal_5191	France	Hautes-Alpes	6.1795	44.7104
		Gal_5197	France	Hautes-Alpes	6.1795	44.7104
		Gal_5769	France	Savoie	6.4008	45.4881
		Gal_5351	Spain	Aragon	0.6677	42.5557
		Gal_5357	Spain	Aragon	0.6161	42.5557
		Gal_5244	France	Pyrénées-Orientales	2.2186	42.3861
		Gal_5246	France	Pyrénées-Orientales	2.1543	42.3894
		Gcl_0044	France	Haute-Savoie	6.4196	46.0280

	Gcl_1780	Switzerland	Valais	7.5563	46.1350
	Gcl_0054	Italy	Bolzano	11.9689	46.5577
	Gcl_0075	Italy	Belluno	12.2292	46.5312
	Gcl_0078	Italy	Belluno	12.1041	46.4912
	Gcl_0084	Italy	Trento	11.6346	46.4552
	Gcl_0090	France	Haute-Savoie	6.2968	45.8389
	Gcl_1160	Switzerland	Valais	7.1077	46.1700
	Gcl_0099	France	Savoie	6.7023	45.7240
	Gcl_0153	Switzerland	Vaud	6.4661	46.7726
	Gcl_1331	Switzerland	Berne	7.0512	47.1308
	Gcl_1337	Switzerland	Berne	7.3460	47.2475
	Gcl_0165	France	Haute-Savoie	6.5813	46.1823
	Gcl_5006	Austria	Styrie	15.6988	47.6844
	Gcl_5048	Austria	Carinthie	14.4875	46.5031
	Gcl_5049	Austria	Carinthie	14.4862	46.5025
	Gcl_5056	Austria	Carinthie	14.4876	46.5021
clusii	Gcl_5094	Switzerland	Grisons	10.2942	46.6432
	Gcl_5068	Austria	Carinthie	13.2995	46.5678
	Gcl_5074	Italy	Udine	12.9537	46.6033
	Gcl_5231	France	Savoie	6.0458	45.6310
	Gcl_5258	Austria	Vorarlberg	10.1541	47.1577
	Gcl_5264	Austria	Northern Tirol	11.7653	47.4453
	Gcl_5285	Austria	Styrie	13.6876	47.4456
	Gcl_5236	France	Savoie	6.0411	45.6304
	Gcl_5760	Switzerland	Valais	6.8911	46.0539
	Gcl_6526	Switzerland	Vaud	7.1964	46.3243
	Gcl_5315	Spain	Aragon	0.0259	42.6558
	Gcl_0180	Italy	Trento	11.0400	46.2125
	Gcl_0184	Italy	Trento	10.7267	45.8756
	Gcl_0187	Italy	Brescia	10.6992	45.8314
	Gcl_0189	Italy	Brescia	10.7125	45.8253
	Gcl_0191	Italy	Verona	10.9642	45.7019
	Gcl_0198	Italy	Brescia	10.4992	45.7914
	Gcl_0202	Italy	Brescia	10.3042	45.9864
	Gcl_0205	Italy	Brescia	10.2667	46.0125
	Lca_0041	France	Jura	6.0801	46.4923
	Lca_1756	Switzerland	Valais	6.9704	46.0273
	Lca_1799	Switzerland	Valais	7.5684	46.1712
	Lca_1824	Switzerland	Valais	8.3641	46.5610
	Lca_1836	Switzerland	Grisons	8.8186	46.6042
caerulea	Lca_0057	Italy	Bolzano	11.6412	46.5429
	Lca_1155	Switzerland	Valais	7.1153	46.0234
	Lca_0144	Italy	Aosta	7.6577	45.5912
	Lca_1130	France	Hautes-Alpes	6.9889	44.8131
	Lca_5271	Austria	Northern Tirol	11.7653	47.4453
	Lca_5297	Austria	Styrie	13.6838	47.4365
	Lca_5063	Austria	Carinthie	14.4983	46.5030
	Lal_1529	France	Haute-Savoie	5.9172	46.0986
	Lal_1541	France	Savoie	5.8639	45.4836
	Lal_0026	France	Ain	5.7333	46.0893
	Lal_1673	France	Hautes-Alpes	5.9491	44.6588
	Lal_1705	France	Hautes-Alpes	5.9077	44.6113

	alpigena	Lal_0047	France	Haute-Savoie	6.4378	46.0401
		Lal_0069	Italy	Belluno	12.2589	46.5850
		Lal_0093	France	Haute-Savoie	6.3060	45.8405
		Lal_5291	Austria	Styrie	13.6842	47.4390
		Lal_5018	Austria	Styrie	15.6992	47.6816
		Lal_5039	Austria	Carinthie	14.5228	46.4927
		Lal_5163	France	Alpes-de-Haute-Provence	6.7400	44.4792
	nigra	Lni_1534	France	Haute-Savoie	5.9172	46.0986
		Lni_1544	France	Savoie	5.8699	45.4527
		Lni_1588	Switzerland	Vaud	6.2648	46.5286
		Lni_1670	France	Hautes-Alpes	5.9491	44.6588
		Lni_1731	France	Haute-Savoie	6.8984	45.9425
		Lni_1800	Switzerland	Valais	7.5684	46.1712
		Lni_1810	Switzerland	Valais	8.1384	46.3887
		Lni_0066	Italy	Belluno	12.2589	46.5850
		Lni_0096	France	Haute-Savoie	6.3060	45.8405
Lonicera		Lni_1152	Switzerland	Valais	7.1153	46.0234
		Lni_1120	France	Hautes-Alpes	6.9543	44.8092
		Lni_5279	Austria	Northern Tirol	12.4157	47.3692
		Lni_5288	Austria	Styrie	13.6842	47.4390
		Lni_5015	Austria	Styrie	15.6989	47.6825
		Lni_5065	Austria	Carinthie	14.5055	46.5029
		Lni_5160	France	Alpes-de-Haute-Provence	6.7400	44.4792
	xylosteum	Lxy_1504	Switzerland	Genève	6.1236	46.2895
		Lxy_1514	France	Ain	6.0856	46.3593
		Lxy_1517	France	Ain	5.8901	46.1692
		Lxy_1533	France	Haute-Savoie	5.9172	46.0986
		Lxy_0002	France	Ain	5.5718	45.7600
		Lxy_1551	France	Savoie	5.9256	45.5132
		Lxy_1579	Switzerland	Vaud	6.2320	46.4232
		Lxy_1591	Switzerland	Vaud	6.4420	46.6447
		Lxy_1637	France	Isère	5.6193	45.0088
		Lxy_1676	France	Hautes-Alpes	5.9491	44.6588
		Lxy_1768	Switzerland	Valais	7.4846	46.2929
		Lxy_1808	Switzerland	Valais	8.1384	46.3887
		Lxy_1090	France	Hautes-Pyrénées	-0.0398	42.9178
		Lxy_1095	France	Pyrénées Atlantiques	-0.3832	42.9712
		Lxy_1004	Switzerland	Vaud	6.8451	46.4632
		Lxy_5299	Austria	Styrie	13.6816	47.4348
		Lxy_5030	Austria	Styrie	14.7988	47.2129
		Lxy_5042	Austria	Carinthie	14.5228	46.4927
		Lxy_5154	France	Alpes-de-Haute-Provence	6.7712	44.4714
		Lxy_5502	France	Hautes-Alpes	6.0524	44.5746
		Lxy_5555	France	Alpes-de-Haute-Provence	5.8059	43.8978
		Lxy_5653	France	Var	5.8188	43.3507
		Lxy_5744	Switzerland	Lucerne	8.2733	47.0077
		Lxy_5833	Czech Republic	Prague	14.1542	49.9537
		Lxy_6271	Sweden	Uppland	17.6214	59.8417
		Lxy_5839	Switzerland	Vaud	7.0361	46.2658
		Lxy_5860	Switzerland	Valais	7.4593	46.1708
	columbinum	Gco_1717	France	Hautes-Alpes	5.8652	44.5580
		Gco_5365	Italy	Foccia	15.5000	42.1169

		Gco_5398	Spain	Catalunya	0.3652	42.3214
Geranium	dissectum	Gdi_1613	Switzerland	Vaud	6.6063	46.6858
		Gdi_0038	Switzerland	Genève	6.0103	46.1995
		Gdi_1618	France	Isère	5.6933	45.0820
		Gdi_5540	France	Alpes-de-Haute-Provence	5.9510	44.1252
		Gdi_5561	France	Alpes-de-Haute-Provence	5.8059	43.8978
		Gdi_5599	France	Var	6.0771	43.3209
		Gdi_5615	France	Var	6.0919	43.1278
		Gdi_5635	France	Var	5.9744	43.2743
		Gdi_5673	France	Vaucluse	5.2879	43.8433
		Gdi_5395	Spain	Asturias	-4.6444	43.0948
	pusillum	Gpu_1812	Switzerland	Valais	8.1356	46.3932
		Gpu_1322	France	Hautes-Pyrénées	-0.0231	43.1497
		Gpu_1325	France	Aude	1.9525	43.4408
Veronica	persica	Vpe_1509	France	Ain	6.0895	46.3421
		Vpe_1523	France	Haute-Savoie	5.8930	46.1083
		Vpe_1563	France	Haute-Savoie	5.9157	45.9739
		Vpe_1582	Switzerland	Vaud	6.2568	46.4569
		Vpe_1600	Switzerland	Vaud	6.4420	46.6447
		Vpe_1615	France	Isère	5.6933	45.0820
		Vpe_1708	France	Hautes-Alpes	5.8652	44.5580
		Vpe_1319	France	Hautes-Pyrénées	-0.0231	43.1497
		Vpe_1102	France	Morbihan	3.1333	47.6000
		Vpe_1105	France	Morbihan	3.1333	47.6000
		Vpe_5508	France	Hautes-Alpes	6.0524	44.5746
		Vpe_5522	France	Hautes-Alpes	6.0463	44.4556
		Vpe_5534	France	Hautes-Alpes	5.8951	44.2958
		Vpe_5543	France	Alpes-de-Haute-Provence	5.9510	44.1252
		Vpe_5576	France	Alpes-de-Haute-Provence	5.9854	43.8336
		Vpe_5605	France	Var	6.2023	42.9968
		Vpe_5607	France	Var	6.1982	42.9973
		Vpe_5617	France	Var	6.0919	43.1278
		Vpe_5679	France	Vaucluse	5.2879	43.8433
		Vpe_5366	Italy	Foccia	15.5000	42.1169
		Vpe_5128	Spain	Asturias	-4.6444	43.0948
	arvensis	Var_1585	Switzerland	Vaud	6.2568	46.4569
		Var_1611	Switzerland	Vaud	6.6063	46.6858
		Var_1621	France	Isère	5.6926	45.0687
		Var_1685	France	Hautes-Alpes	5.8656	44.6712
		Var_1815	Switzerland	Valais	8.1356	46.3932
		Var_5528	France	Hautes-Alpes	5.8951	44.2958
		Var_5537	France	Alpes-de-Haute-Provence	5.9510	44.1252
		Var_5564	France	Alpes-de-Haute-Provence	5.8059	43.8978
		Var_5570	France	Alpes-de-Haute-Provence	5.9275	43.8123
		Var_5582	France	Alpes-de-Haute-Provence	6.0808	43.7608
		Var_5610	France	Var	6.0919	43.1278
		Var_5641	France	Var	5.9744	43.2743
		Var_5670	France	Vaucluse	5.2879	43.8433
		Var_5377	Italy	Foccia	15.5000	42.1169
		Vpo_1688	France	Hautes-Alpes	5.8656	44.6712
		Vpo_1711	France	Hautes-Alpes	5.8652	44.5580
		Vpo_1802	Switzerland	Valais	8.0208	46.3270

polita	Vpo_1818	Switzerland	Valais	8.1356	46.3932
	Vpo_5531	France	Hautes-Alpes	5.8951	44.2958
	Vpo_5567	France	Alpes-de-Haute-Provence	5.9275	43.8123
	Vpo_5579	France	Alpes-de-Haute-Provence	5.9854	43.8336
	Vpo_5602	France	Var	6.1414	43.1523
	Vpo_5620	France	Var	6.0626	43.1195
	Vpo_5701	France	Vaucluse	5.2879	43.8433
hederifolia	Vhe_1507	France	Ain	6.0895	46.3421
	Vhe_1566	France	Haute-Savoie	5.9157	45.9739
	Vhe_1569	Switzerland	Vaud	6.2320	46.4232
	Vhe_1602	Switzerland	Vaud	6.4420	46.6447
	Vhe_1649	France	Isère	5.5550	44.8228
	Vhe_1664	France	Hautes-Alpes	5.9449	44.6955
	Vhe_1714	France	Hautes-Alpes	5.8652	44.5580
	Vhe_5573	France	Alpes-de-Haute-Provence	5.9275	43.8123
	Vhe_5613	France	Var	6.0919	43.1278
	Vhe_5623	France	Var	6.0626	43.1195
	Vhe_5638	France	Var	5.9744	43.2743
	Vhe_5676	France	Vaucluse	5.2879	43.8433
	Vhe_5857	Switzerland	Valais	7.1495	46.0993
