# Supplementary material for: Evolutionary histories determine DNA barcoding success in vascular plants: seven case studies using intraspecific broad sampling of closely related species
Source: BMC Evol Biol. 2016 May 13;16:103. doi: 10.1186/s12862-016-0678-0 (PMC4866073; doi:10.1186/s12862-016-0678-0)
Supplement: Additional file 2: — Amplification conditions. For each species, annealing temperature and the primer combination are given for matK, rpoC1, rpoB, and trnH-psbA. (PDF 27 kb) [file 12862_2016_678_MOESM2_ESM.pdf]

		n	*mat*K		*rpo*C1		*rpo*B		*trn*H-*psb*A	
			Annealing (°C)	Primer Combination	Annealing (°C)	Primer Combination	Annealing (°C)	Primer Combination	Annealing (°C)	Primer Combination
Acer	campestre	27	45	XF-5R	50	1F-3R	50	1F-3R	52	Shaw *et al.* 2005
	platanoides	15	45	XF-5R	50	1F-3R	50	1F-3R	52	Shaw *et al.* 2005
	opalus	14	45	XF-5R	50	1F-3R	50	1F-3R	52	Shaw *et al.* 2005
	monspessulanum	11	45	XF-5R	50	1F-3R	50	1F-3R	52	Shaw *et al.* 2005
	pseudoplatanus	36	45	2.1F-3.2R	50	1F-3R	50	1F-3R	52	Shaw *et al.* 2005
Salix	herbacea	16	45	2.1F-3.2R	50	1F-3R	50	1F-3R	52	Shaw *et al.* 2005
	reticulata	18	45	2.1F-3.2R	50	1F-3R	50	1F-3R	52	Shaw *et al.* 2005
	retusa	22	45	2.1F-3.2R	50	1F-3R	50	1F-3R	52	Shaw *et al.* 2005
	serpillifolia	13	45	2.1F-3.2R	50	1F-3R	50	1F-3R	52	Shaw *et al.* 2005
Adenostyles	alliariae	14	48	2.1F-5R	50	1F-3R	50	1F-3R	52	Shaw *et al.* 2005
	glabra	16	48	2.1F-5R	50	1F-3R	50	1F-3R	52	Shaw *et al.* 2005
	leucophylla	7	48	2.1F-5R	50	1F-3R	50	1F-3R	52	Shaw *et al.* 2005
Gentiana	angustifolia	31	50	2.1F-5R	50	1F-3R	50	1F-3R	52	Shaw *et al.* 2005
	acaulis	45	50	2.1F-5R	50	1F-3R	50	1F-3R	52	Shaw *et al.* 2005
	alpina	23	50	2.1F-5R	50	1F-3R	50	1F-3R	52	Shaw *et al.* 2005
	clusii	36	50	2.1F-5R	50	1F-3R	50	1F-3R	52	Shaw *et al.* 2005
Lonicera	caerulea	12	47	bcd1F-5R	50	1F-3R	50	1F-3R	52	Shaw *et al.* 2005
	alpigena	12	47	bcd1F-5R	50	1F-3R	50	1F-3R	52	Shaw *et al.* 2005
	nigra	16	47	bcd1F-5R	50	1F-3R	50	1F-3R	52	Shaw *et al.* 2005
	xylosteum	27	47	bcd1F-5R	50	1F-3R	50	1F-3R	52	Shaw *et al.* 2005
Geranium	columbinum	3	45	2.1F-5R	50	1F-3R	50	1F-3R	52	Shaw *et al.* 2005
	dissectum	10	45	2.1F-5R	50	1F-3R	50	1F-3R	52	Shaw *et al.* 2005
	pusillum	3	45	2.1F-5R	50	1F-3R	50	1F-3R	52	Shaw *et al.* 2005
Veronica	persica	21	47	bcd1F-5R	50	1F-3R	50	1F-3R	52	Shaw *et al.* 2005
	arvensis	14	47	bcd1F-5R	50	1F-3R	50	1F-3R	52	Shaw *et al.* 2005
	polita	10	47	bcd1F-5R	50	1F-3R	50	1F-3R	52	Shaw *et al.* 2005
	hederifolia	13	-	-	50	1F-3R	50	1F-3R	52	Shaw *et al.* 2005
