# Supplementary material for: Evolutionary histories determine DNA barcoding success in vascular plants: seven case studies using intraspecific broad sampling of closely related species
Source: BMC Evol Biol. 2016 May 13;16:103. doi: 10.1186/s12862-016-0678-0 (PMC4866073; doi:10.1186/s12862-016-0678-0)

Acer
*mat*K

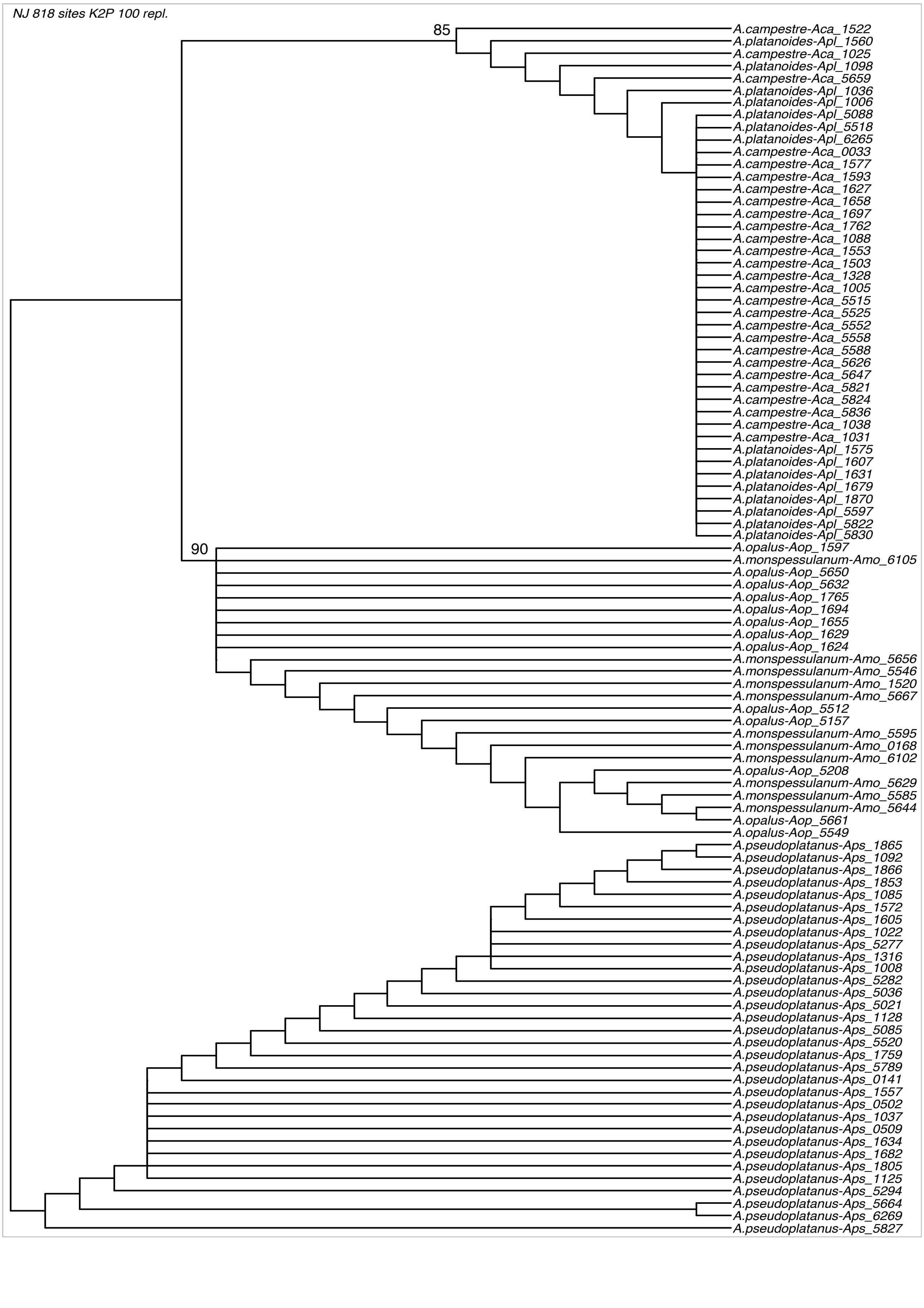
NJ 818 sites K2P 100 repl.
85
90
A.campestre-Aca_1522
A.platanoides-Apl_1560
A.campestre-Aca_1025
A.platanoides-Apl_1098
A.campestre-Aca_5659
A.platanoides-Apl_1036
A.platanoides-Apl_1006
A.platanoides-Apl_5088
A.platanoides-Apl_5518
A.platanoides-Apl_6265
A.campestre-Aca_0033
A.campestre-Aca_1577
A.campestre-Aca_1593
A.campestre-Aca_1627
A.campestre-Aca_1658
A.campestre-Aca_1697
A.campestre-Aca_1762
A.campestre-Aca_1088
A.campestre-Aca_1553
A.campestre-Aca_1503
A.campestre-Aca_1328
A.campestre-Aca_1005
A.campestre-Aca_5515
A.campestre-Aca_5525
A.campestre-Aca_5552
A.campestre-Aca_5558
A.campestre-Aca_5588
A.campestre-Aca_5626
A.campestre-Aca_5647
A.campestre-Aca_5821
A.campestre-Aca_5824
A.campestre-Aca_5836
A.campestre-Aca_1038
A.campestre-Aca_1031
A.platanoides-Apl_1575
A.platanoides-Apl_1607
A.platanoides-Apl_1631
A.platanoides-Apl_1679
A.platanoides-Apl_1870
A.platanoides-Apl_5597
A.platanoides-Apl_5822
A.platanoides-Apl_5830
A.opalus-Aop_1597
A.monspessulanum-Amo_6105
A.opalus-Aop_5650
A.opalus-Aop_5632
A.opalus-Aop_1765
A.opalus-Aop_1694
A.opalus-Aop_1655
A.opalus-Aop_1629
A.opalus-Aop_1624
A.monspessulanum-Amo_5656
A.monspessulanum-Amo_5546
A.monspessulanum-Amo_1520
A.monspessulanum-Amo_5667
A.opalus-Aop_5512
A.opalus-Aop_5157
A.monspessulanum-Amo_5595
A.monspessulanum-Amo_0168
A.monspessulanum-Amo_6102
A.opalus-Aop_5208
A.monspessulanum-Amo_5629
A.monspessulanum-Amo_5585
A.monspessulanum-Amo_5644
A.opalus-Aop_5661
A.opalus-Aop_5549
A.pseudoplatanus-Aps_1865
A.pseudoplatanus-Aps_1092
A.pseudoplatanus-Aps_1866
A.pseudoplatanus-Aps_1853
A.pseudoplatanus-Aps_1085
A.pseudoplatanus-Aps_1572
A.pseudoplatanus-Aps_1605
A.pseudoplatanus-Aps_1022
A.pseudoplatanus-Aps_5277
A.pseudoplatanus-Aps_1316
A.pseudoplatanus-Aps_1008
A.pseudoplatanus-Aps_5282
A.pseudoplatanus-Aps_5036
A.pseudoplatanus-Aps_5021
A.pseudoplatanus-Aps_1128
A.pseudoplatanus-Aps_5085
A.pseudoplatanus-Aps_5520
A.pseudoplatanus-Aps_1759
A.pseudoplatanus-Aps_5789
A.pseudoplatanus-Aps_0141
A.pseudoplatanus-Aps_1557
A.pseudoplatanus-Aps_0502
A.pseudoplatanus-Aps_1037
A.pseudoplatanus-Aps_0509
A.pseudoplatanus-Aps_1634
A.pseudoplatanus-Aps_1682
A.pseudoplatanus-Aps_1805
A.pseudoplatanus-Aps_1125
A.pseudoplatanus-Aps_5294
A.pseudoplatanus-Aps_5664
A.pseudoplatanus-Aps_6269
A.pseudoplatanus-Aps_5827

Acer
*rpo*C1

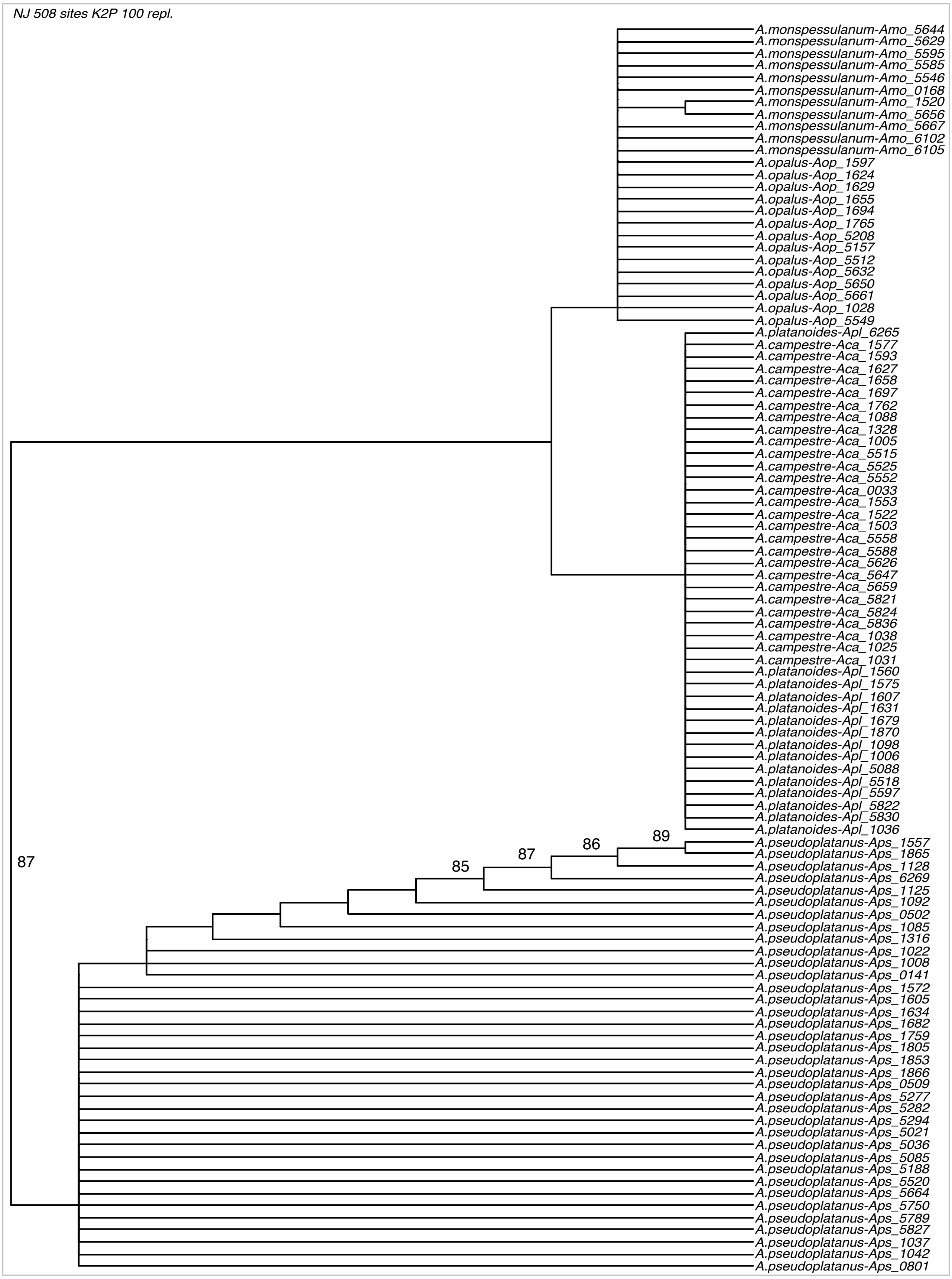
NJ 508 sites K2P 100 repl.
A.monspessulanum-Amo_5644
A.monspessulanum-Amo_5629
A.monspessulanum-Amo_5595
A.monspessulanum-Amo_5585
A.monspessulanum-Amo_5546
A.monspessulanum-Amo_0168
A.monspessulanum-Amo_1520
A.monspessulanum-Amo_5656
A.monspessulanum-Amo_5667
A.monspessulanum-Amo_6102
A.monspessulanum-Amo_6105
A.opalus-Aop_1597
A.opalus-Aop_1624
A.opalus-Aop_1629
A.opalus-Aop_1655
A.opalus-Aop_1694
A.opalus-Aop_1765
A.opalus-Aop_5208
A.opalus-Aop_5157
A.opalus-Aop_5512
A.opalus-Aop_5632
A.opalus-Aop_5650
A.opalus-Aop_5661
A.opalus-Aop_1028
A.opalus-Aop_5549
A.platanoides-Apl_6265
A.campestre-Aca_1577
A.campestre-Aca_1593
A.campestre-Aca_1627
A.campestre-Aca_1658
A.campestre-Aca_1697
A.campestre-Aca_1762
A.campestre-Aca_1088
A.campestre-Aca_1328
A.campestre-Aca_1005
A.campestre-Aca_5515
A.campestre-Aca_5525
A.campestre-Aca_5552
A.campestre-Aca_0033
A.campestre-Aca_1553
A.campestre-Aca_1522
A.campestre-Aca_1503
A.campestre-Aca_5558
A.campestre-Aca_5588
A.campestre-Aca_5626
A.campestre-Aca_5647
A.campestre-Aca_5659
A.campestre-Aca_5821
A.campestre-Aca_5824
A.campestre-Aca_5836
A.campestre-Aca_1038
A.campestre-Aca_1025
A.campestre-Aca_1031
A.platanoides-Apl_1560
A.platanoides-Apl_1575
A.platanoides-Apl_1607
A.platanoides-Apl_1631
A.platanoides-Apl_1679
A.platanoides-Apl_1870
A.platanoides-Apl_1098
A.platanoides-Apl_1006
A.platanoides-Apl_5088
A.platanoides-Apl_5518
A.platanoides-Apl_5597
A.platanoides-Apl_5822
A.platanoides-Apl_5830
A.platanoides-Apl_1036
89
86
87
85
87
A.pseudoplatanus-Aps_1557
A.pseudoplatanus-Aps_1865
A.pseudoplatanus-Aps_1128
A.pseudoplatanus-Aps_6269
A.pseudoplatanus-Aps_1125
A.pseudoplatanus-Aps_1092
A.pseudoplatanus-Aps_0502
A.pseudoplatanus-Aps_1085
A.pseudoplatanus-Aps_1316
A.pseudoplatanus-Aps_1022
A.pseudoplatanus-Aps_1008
A.pseudoplatanus-Aps_0141
A.pseudoplatanus-Aps_1572
A.pseudoplatanus-Aps_1605
A.pseudoplatanus-Aps_1634
A.pseudoplatanus-Aps_1682
A.pseudoplatanus-Aps_1759
A.pseudoplatanus-Aps_1805
A.pseudoplatanus-Aps_1853
A.pseudoplatanus-Aps_1866
A.pseudoplatanus-Aps_0509
A.pseudoplatanus-Aps_5277
A.pseudoplatanus-Aps_5282
A.pseudoplatanus-Aps_5294
A.pseudoplatanus-Aps_5021
A.pseudoplatanus-Aps_5036
A.pseudoplatanus-Aps_5085
A.pseudoplatanus-Aps_5188
A.pseudoplatanus-Aps_5520
A.pseudoplatanus-Aps_5664
A.pseudoplatanus-Aps_5750
A.pseudoplatanus-Aps_5789
A.pseudoplatanus-Aps_5827
A.pseudoplatanus-Aps_1037
A.pseudoplatanus-Aps_1042
A.pseudoplatanus-Aps_0801

Acer

*rpo*B

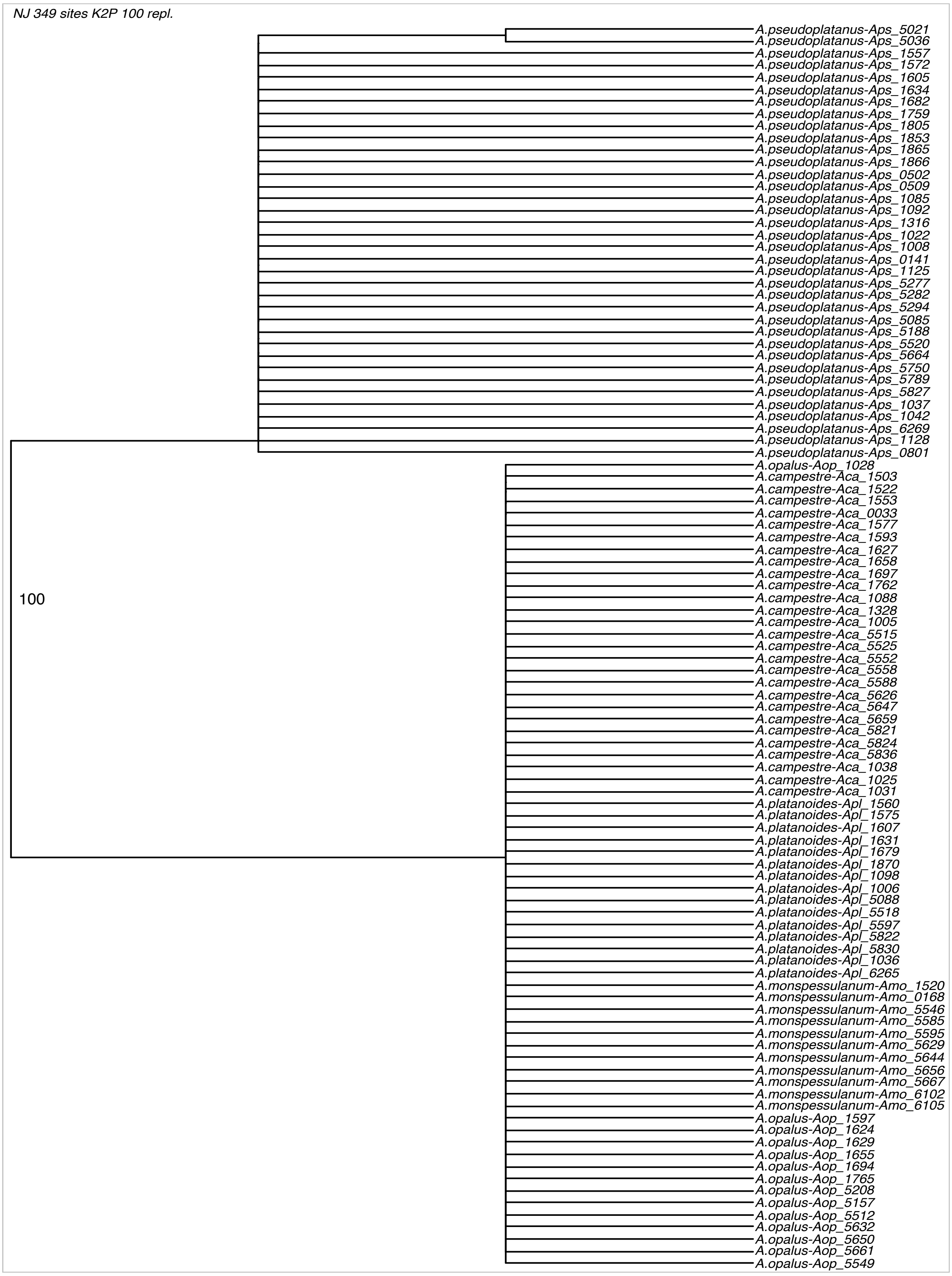

NJ 349 sites K2P 100 repl.
100
A.pseudoplatanus-Aps_5021
A.pseudoplatanus-Aps_5036
A.pseudoplatanus-Aps_1557
A.pseudoplatanus-Aps_1572
A.pseudoplatanus-Aps_1605
A.pseudoplatanus-Aps_1634
A.pseudoplatanus-Aps_1682
A.pseudoplatanus-Aps_1759
A.pseudoplatanus-Aps_1805
A.pseudoplatanus-Aps_1853
A.pseudoplatanus-Aps_1865
A.pseudoplatanus-Aps_1866
A.pseudoplatanus-Aps_0502
A.pseudoplatanus-Aps_0509
A.pseudoplatanus-Aps_1085
A.pseudoplatanus-Aps_1092
A.pseudoplatanus-Aps_1316
A.pseudoplatanus-Aps_1022
A.pseudoplatanus-Aps_1008
A.pseudoplatanus-Aps_0141
A.pseudoplatanus-Aps_1125
A.pseudoplatanus-Aps_5277
A.pseudoplatanus-Aps_5282
A.pseudoplatanus-Aps_5294
A.pseudoplatanus-Aps_5085
A.pseudoplatanus-Aps_5188
A.pseudoplatanus-Aps_5520
A.pseudoplatanus-Aps_5664
A.pseudoplatanus-Aps_5750
A.pseudoplatanus-Aps_5789
A.pseudoplatanus-Aps_5827
A.pseudoplatanus-Aps_1037
A.pseudoplatanus-Aps_1042
A.pseudoplatanus-Aps_6269
A.pseudoplatanus-Aps_1128
A.pseudoplatanus-Aps_0801
A.opalus-Aop_1028
A.campestre-Aca_1503
A.campestre-Aca_1522
A.campestre-Aca_1553
A.campestre-Aca_0033
A.campestre-Aca_1577
A.campestre-Aca_1593
A.campestre-Aca_1627
A.campestre-Aca_1658
A.campestre-Aca_1697
A.campestre-Aca_1762
A.campestre-Aca_1088
A.campestre-Aca_1328
A.campestre-Aca_1005
A.campestre-Aca_5515
A.campestre-Aca_5525
A.campestre-Aca_5552
A.campestre-Aca_5558
A.campestre-Aca_5588
A.campestre-Aca_5626
A.campestre-Aca_5647
A.campestre-Aca_5659
A.campestre-Aca_5821
A.campestre-Aca_5824
A.campestre-Aca_5836
A.campestre-Aca_1038
A.campestre-Aca_1025
A.campestre-Aca_1031
A.platanoides-Apl_1560
A.platanoides-Apl_1575
A.platanoides-Apl_1607
A.platanoides-Apl_1631
A.platanoides-Apl_1679
A.platanoides-Apl_1870
A.platanoides-Apl_1098
A.platanoides-Apl_1006
A.platanoides-Apl_5088
A.platanoides-Apl_5518
A.platanoides-Apl_5597
A.platanoides-Apl_5822
A.platanoides-Apl_5830
A.platanoides-Apl_1036
A.platanoides-Apl_6265
A.monspessulanum-Amo_1520
A.monspessulanum-Amo_0168
A.monspessulanum-Amo_5546
A.monspessulanum-Amo_5585
A.monspessulanum-Amo_5595
A.monspessulanum-Amo_5629
A.monspessulanum-Amo_5644
A.monspessulanum-Amo_5656
A.monspessulanum-Amo_5667
A.monspessulanum-Amo_6102
A.monspessulanum-Amo_6105
A.opalus-Aop_1597
A.opalus-Aop_1624
A.opalus-Aop_1629
A.opalus-Aop_1655
A.opalus-Aop_1694
A.opalus-Aop_1765
A.opalus-Aop_5208
A.opalus-Aop_5157
A.opalus-Aop_5512
A.opalus-Aop_5632
A.opalus-Aop_5650
A.opalus-Aop_5661
A.opalus-Aop_5549

Acer
*trn*H-*psb*A

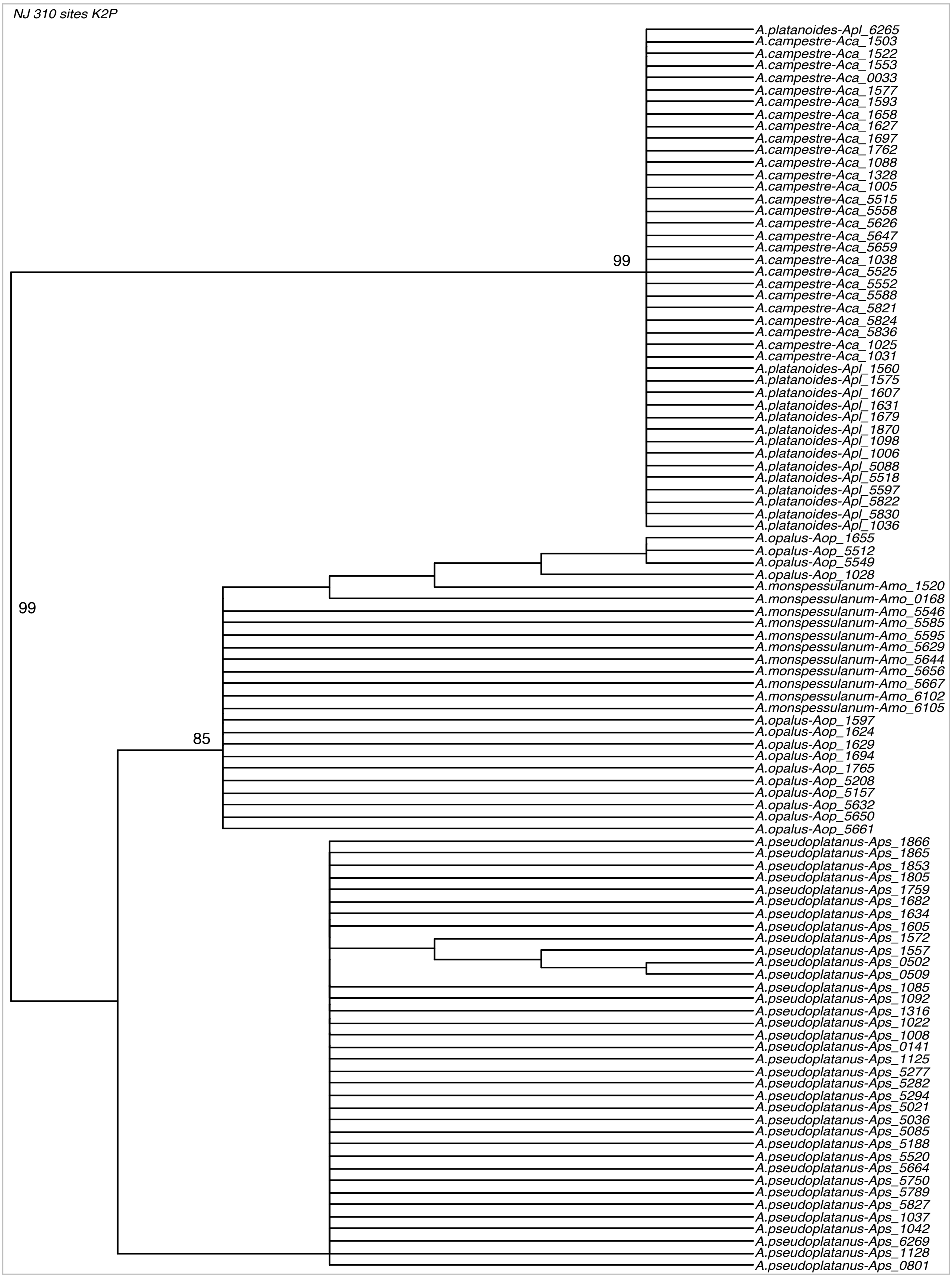

Adenostyles
matK

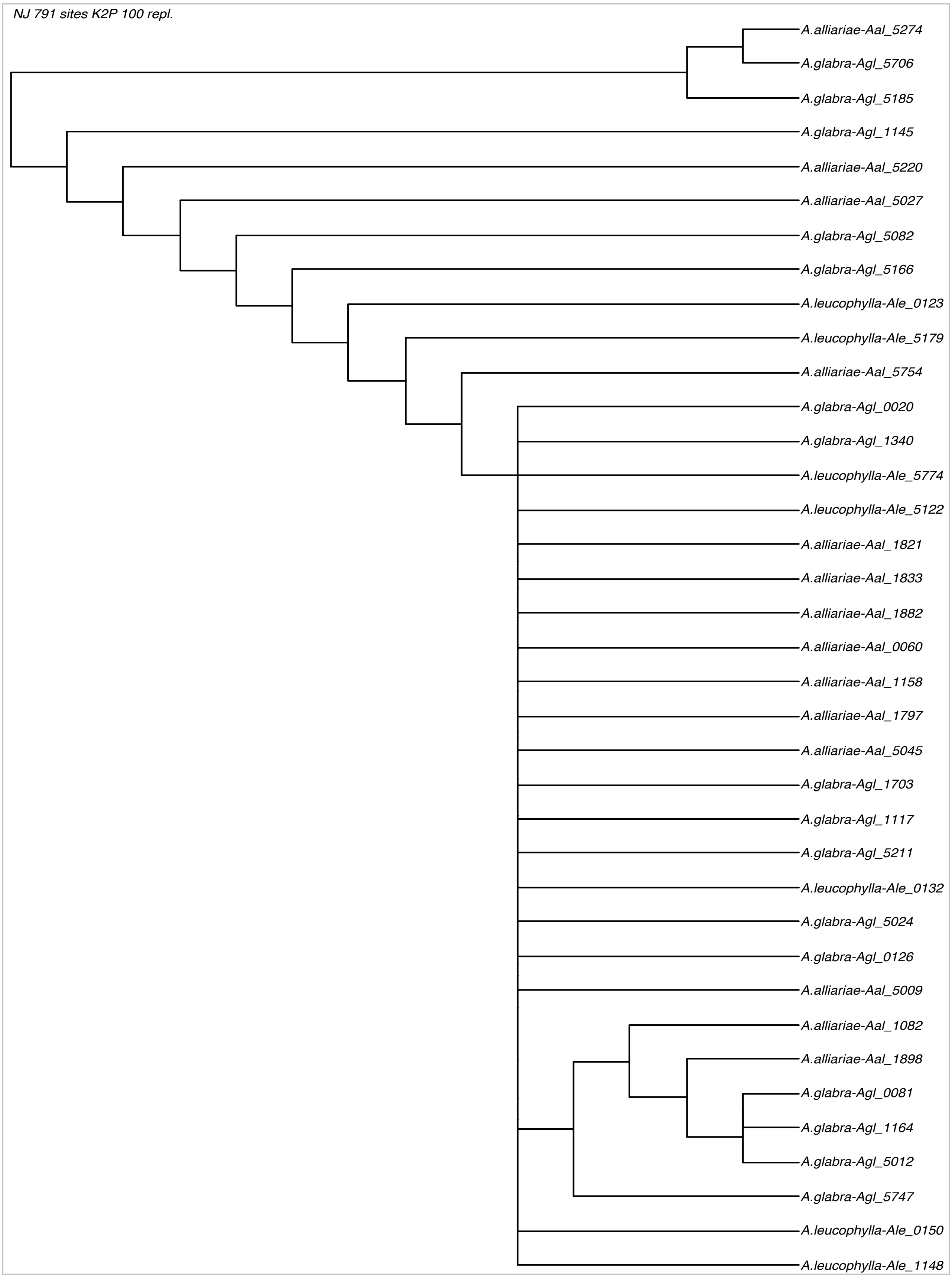

Adenostyles
*rpo*C1

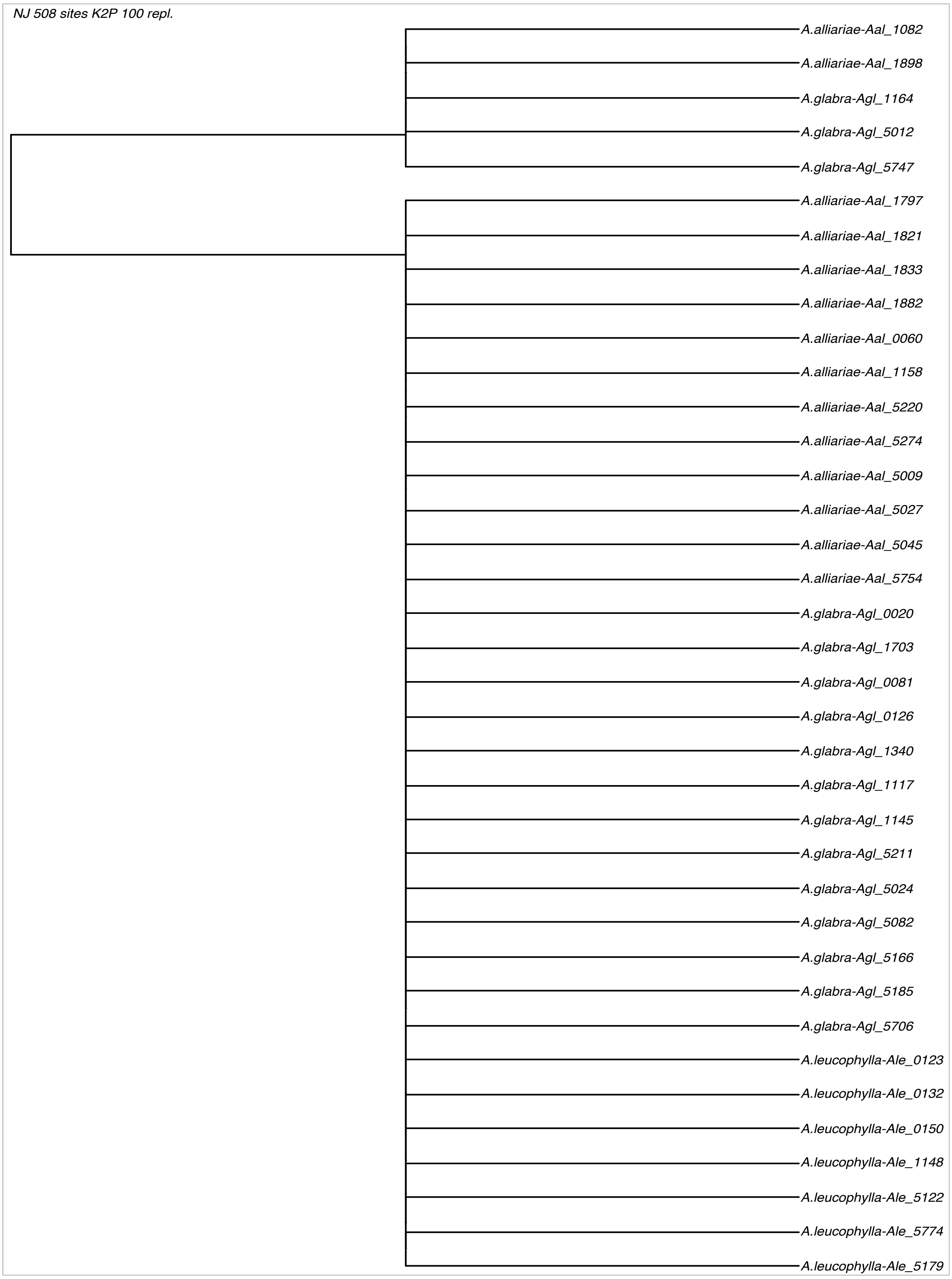

Adenostyles
*rpo*B

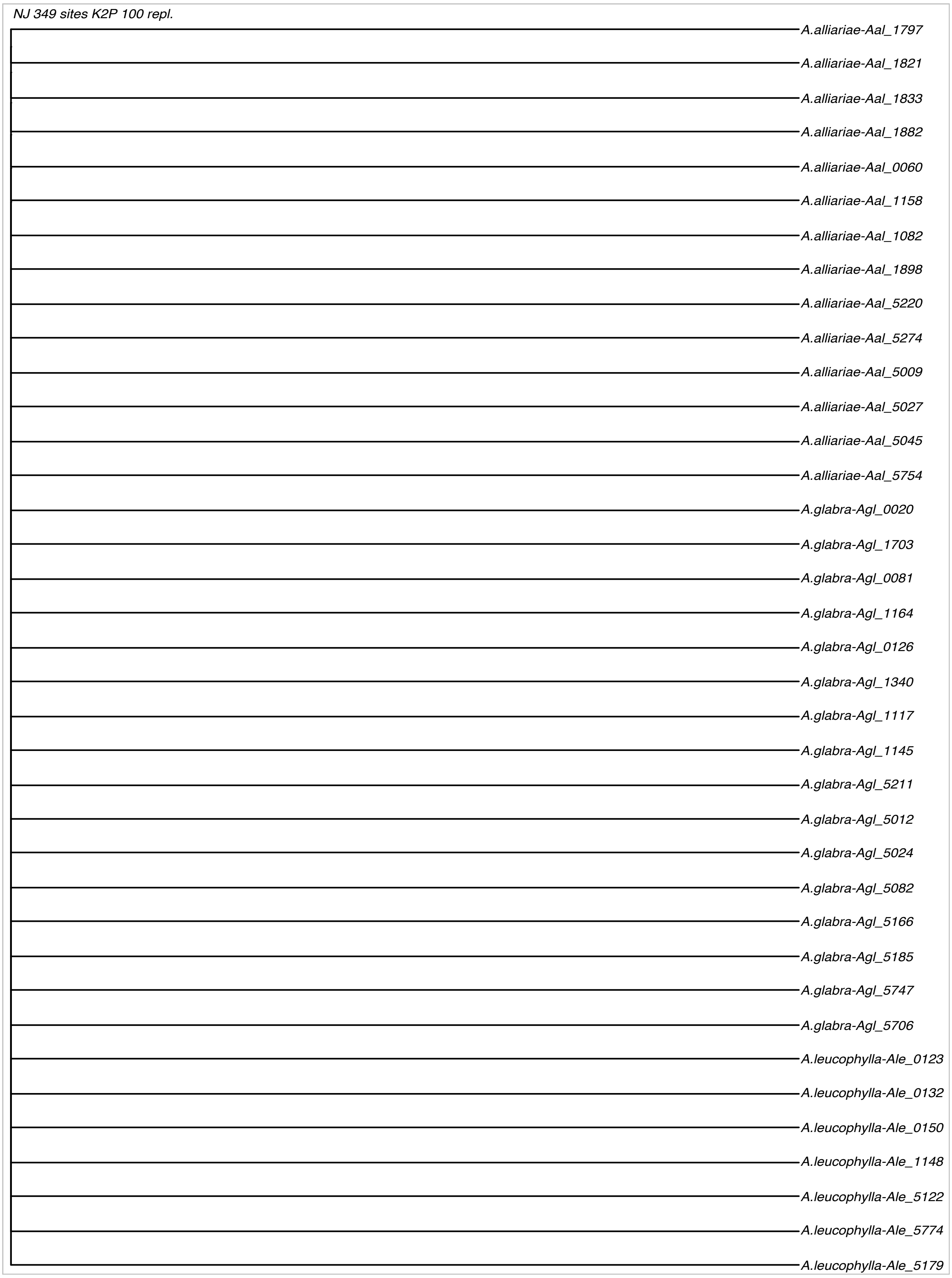

Adenostyles
*trn*H-*psb*A

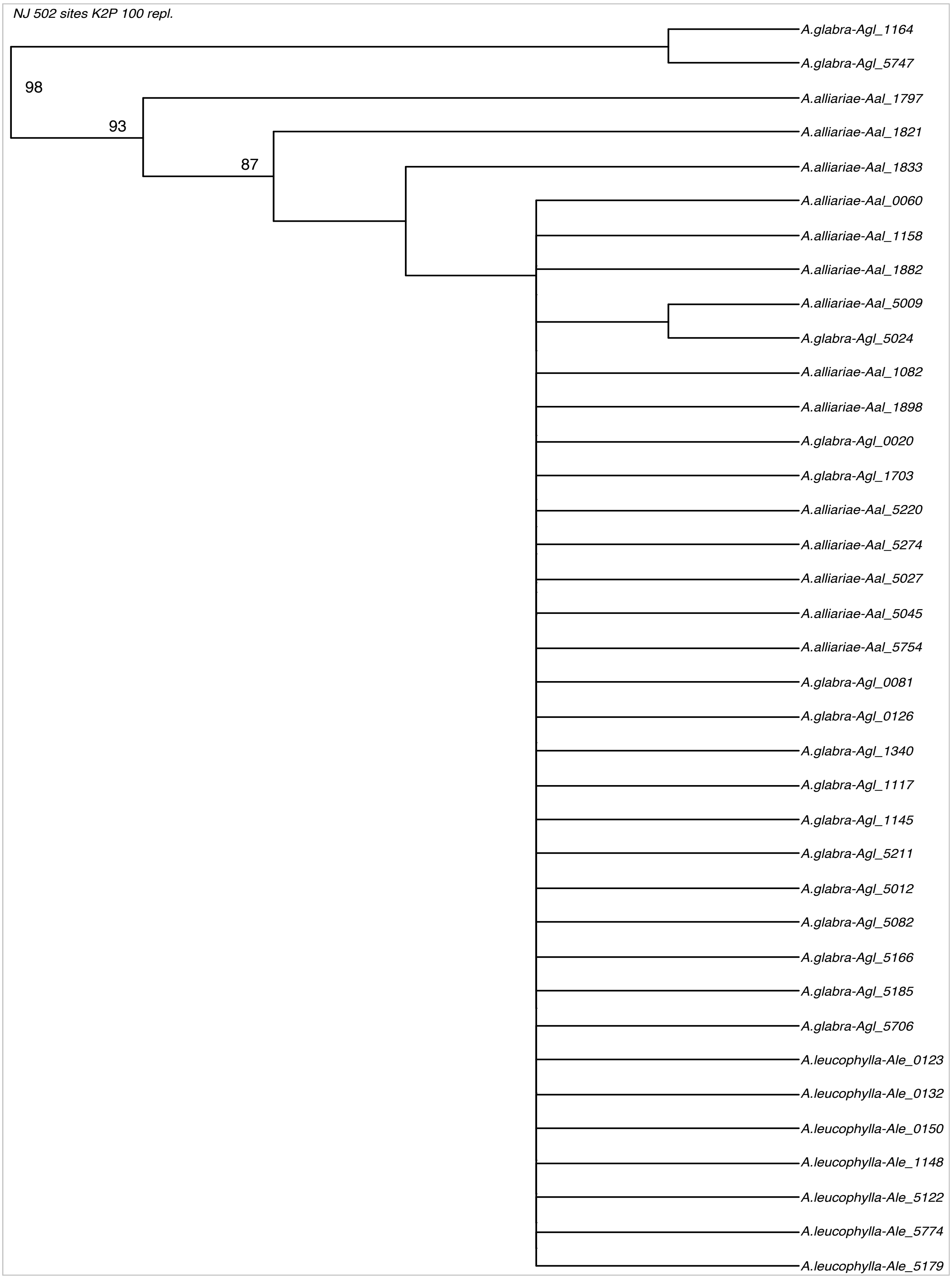

Gentiana
*mat*K

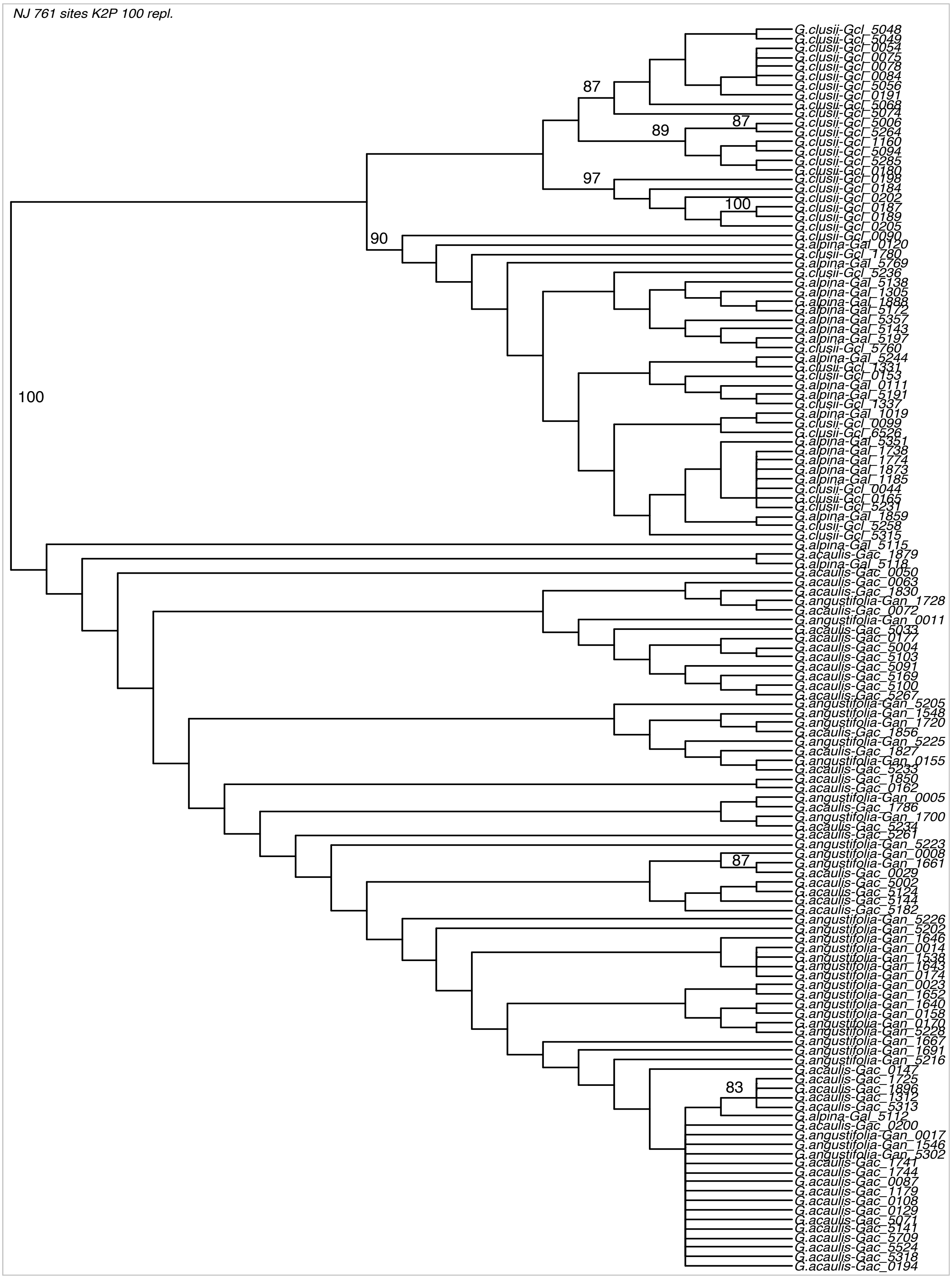

Gentiana
*rpo*C1

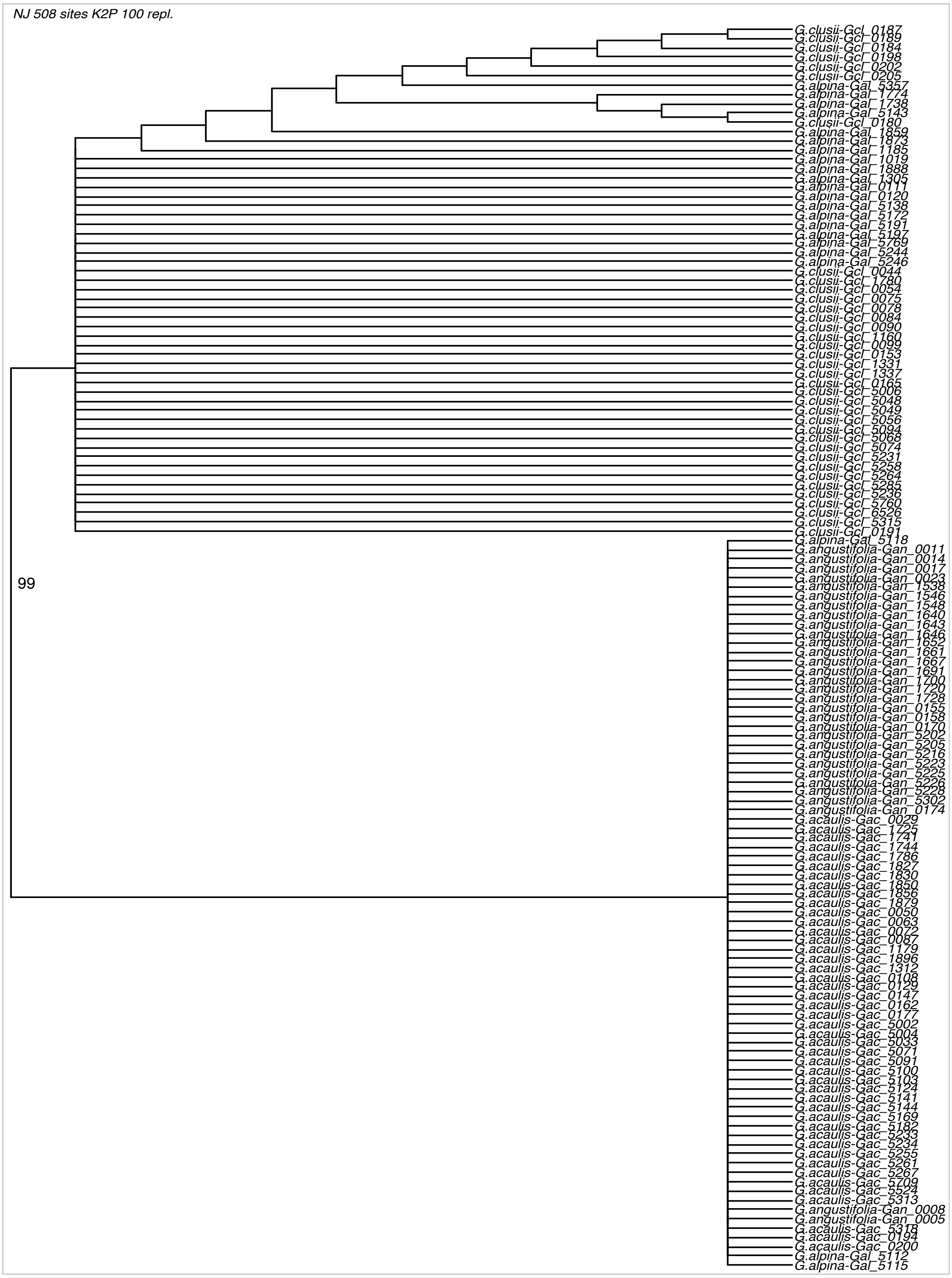

Gentiana
*rpo*B

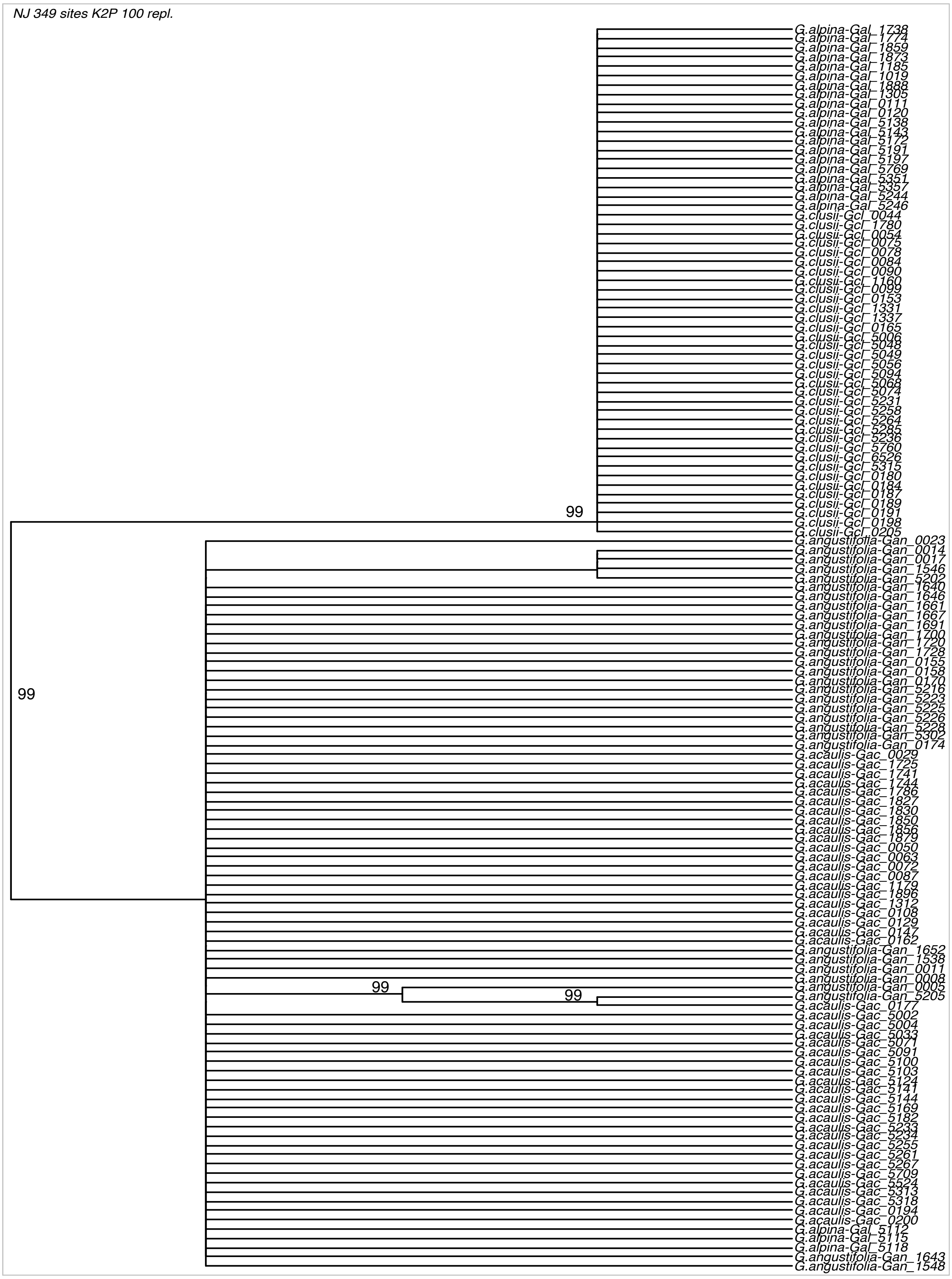

Gentiana

*trn*H-*psb*A

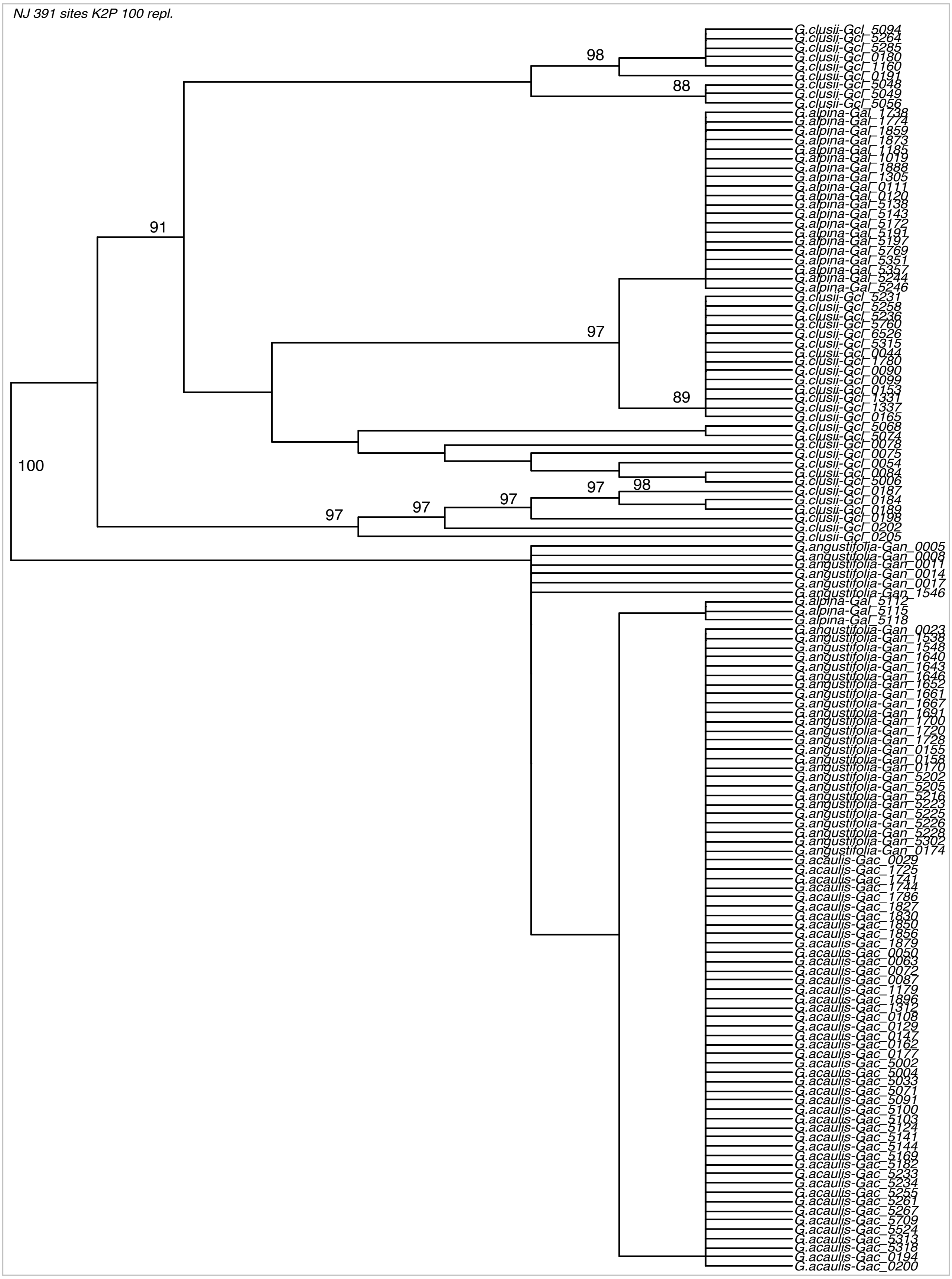

Geranium
matK

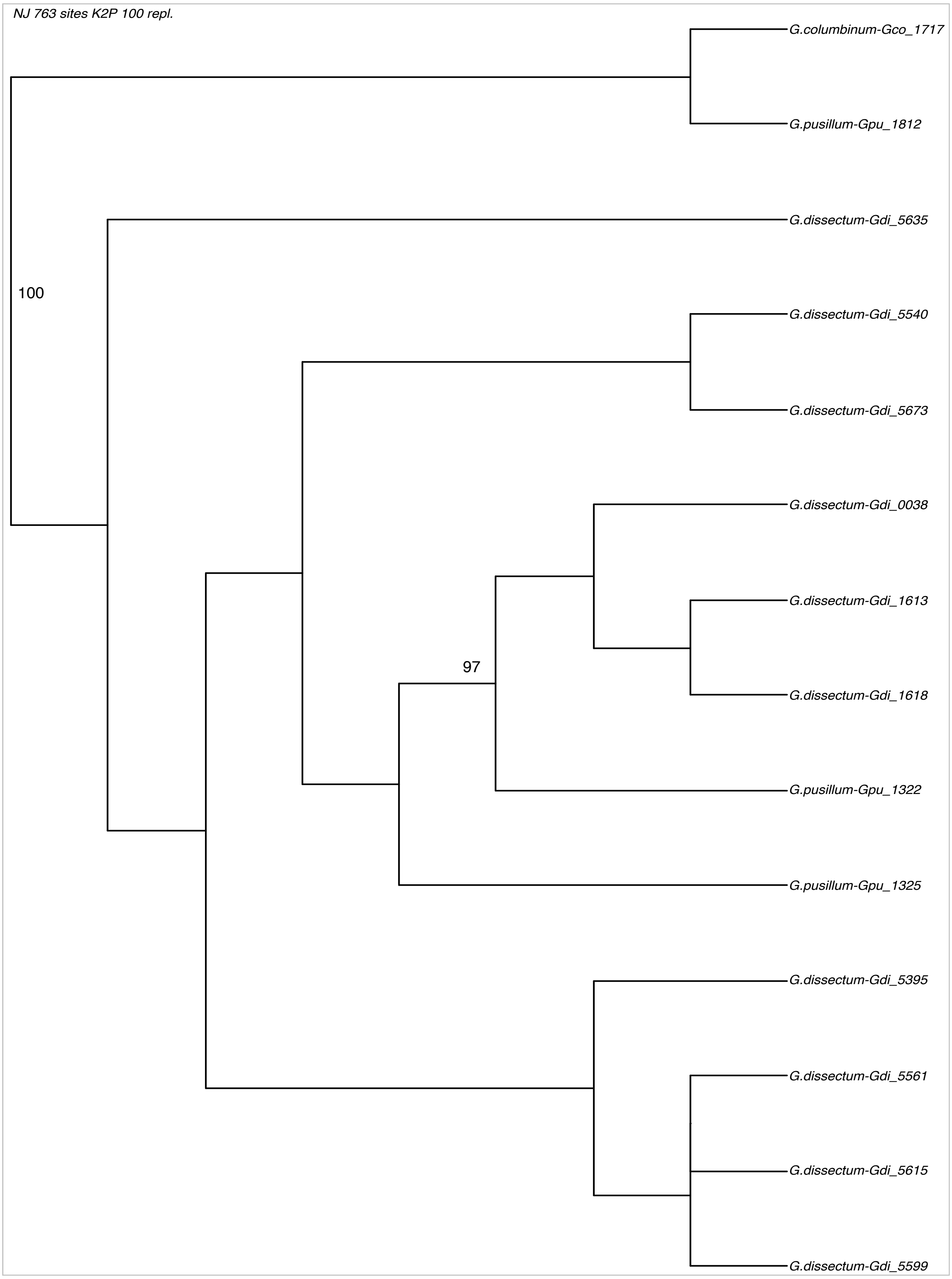

Geranium
*rpo*C1

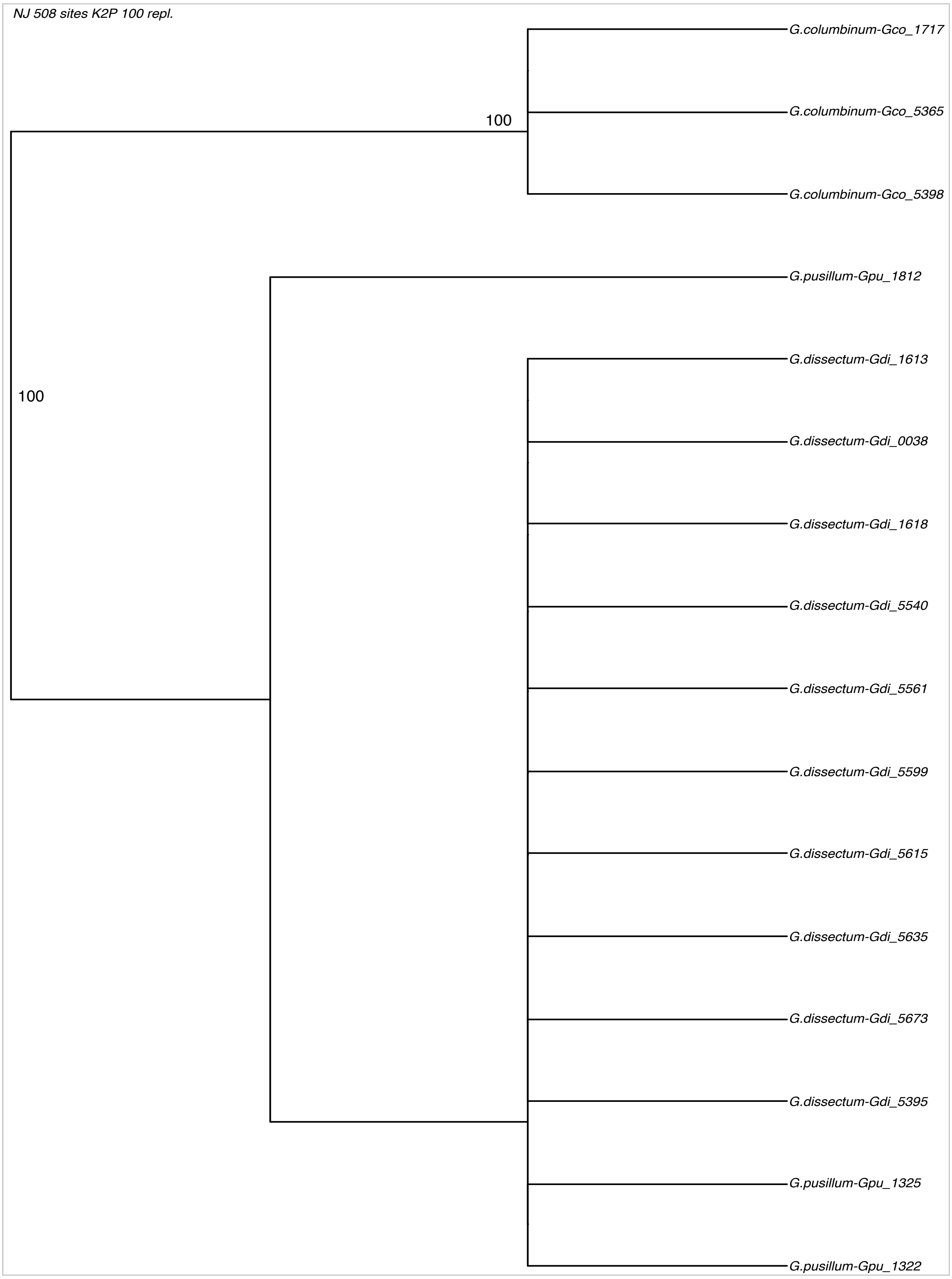

Geranium
*rpo*B

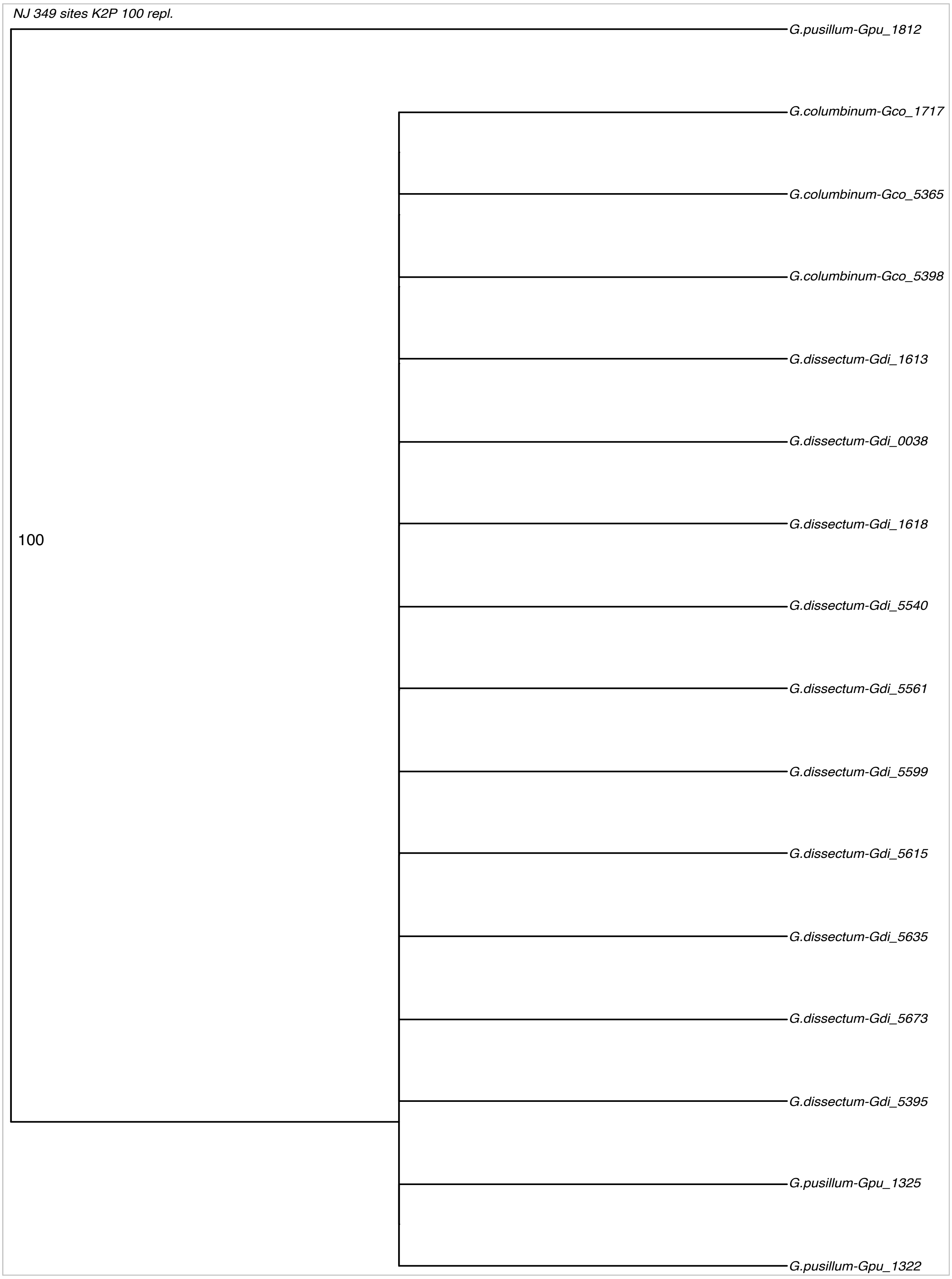

Geranium
*trn*H-*psb*A

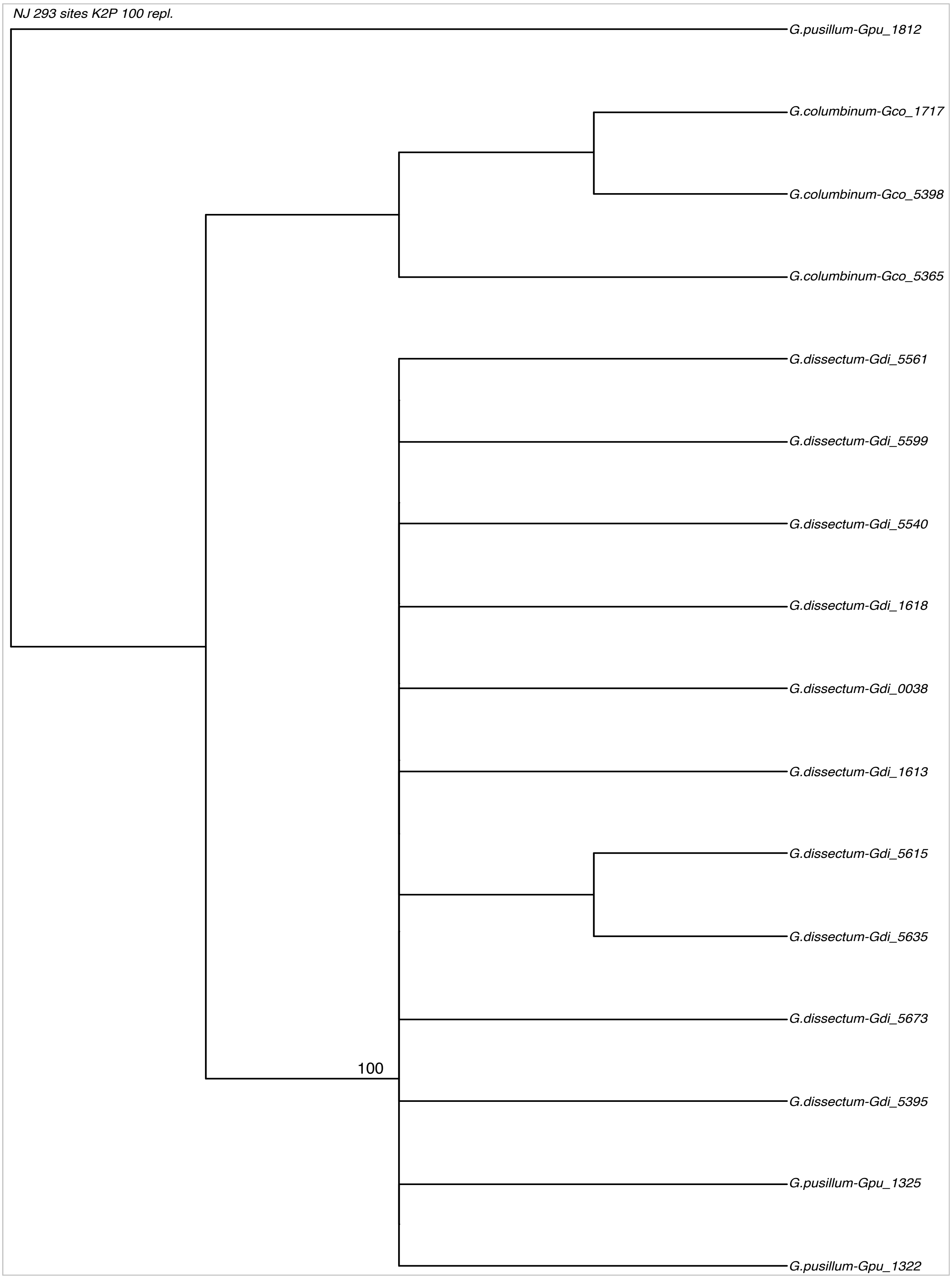

Lonicera
*mat*K

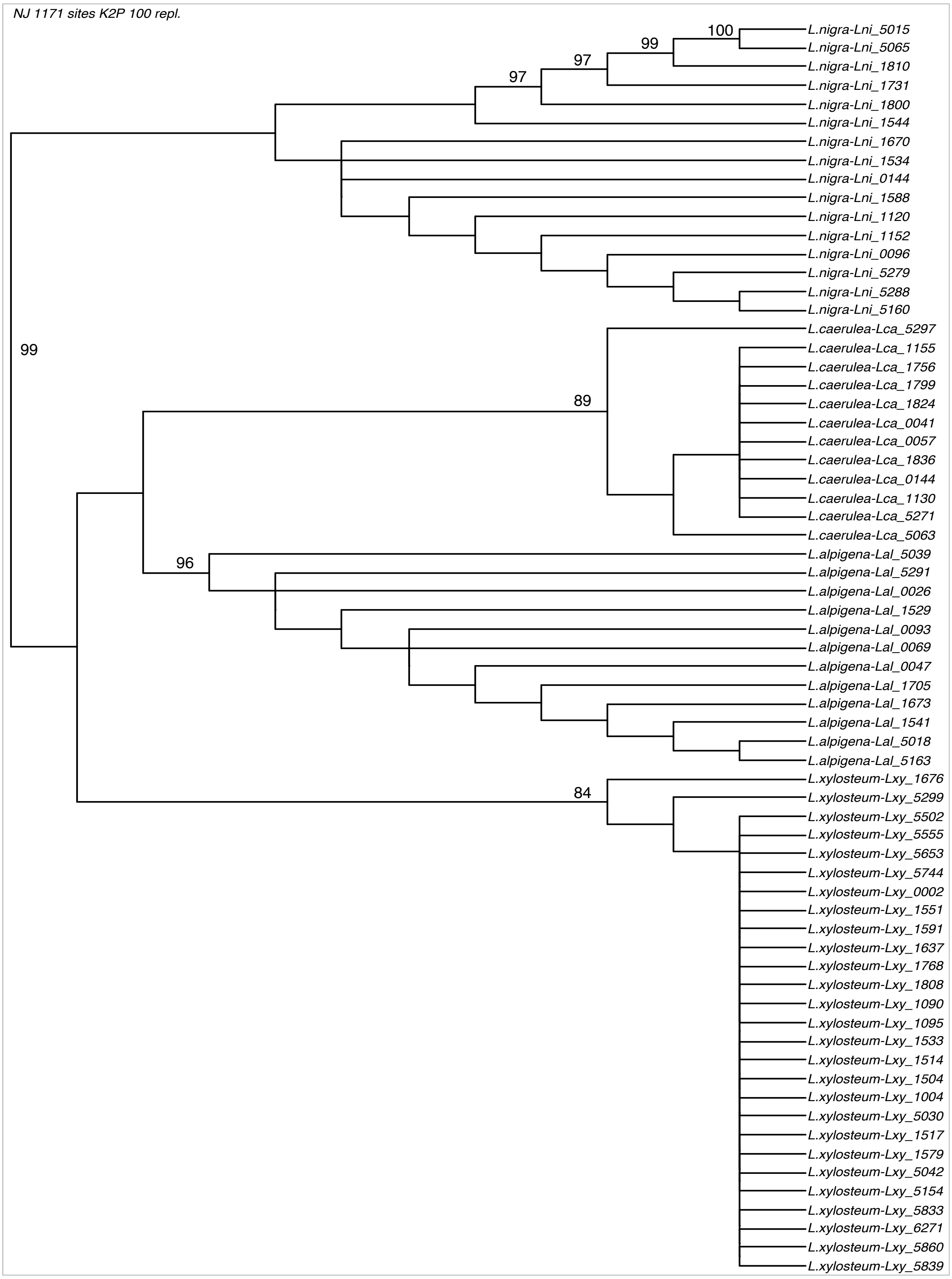

Lonicera
*rpo*C1

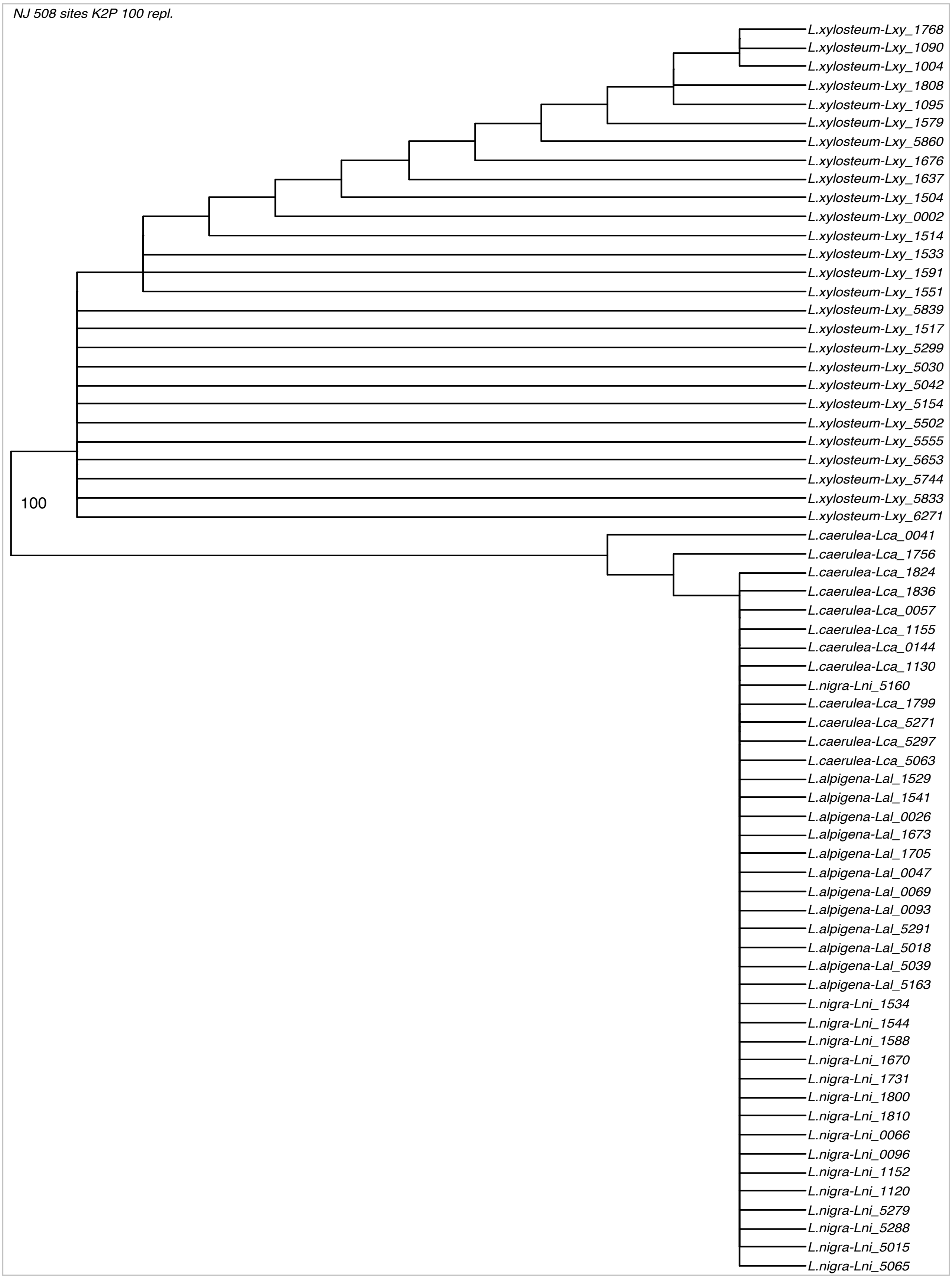

Lonicera
*rpo*B

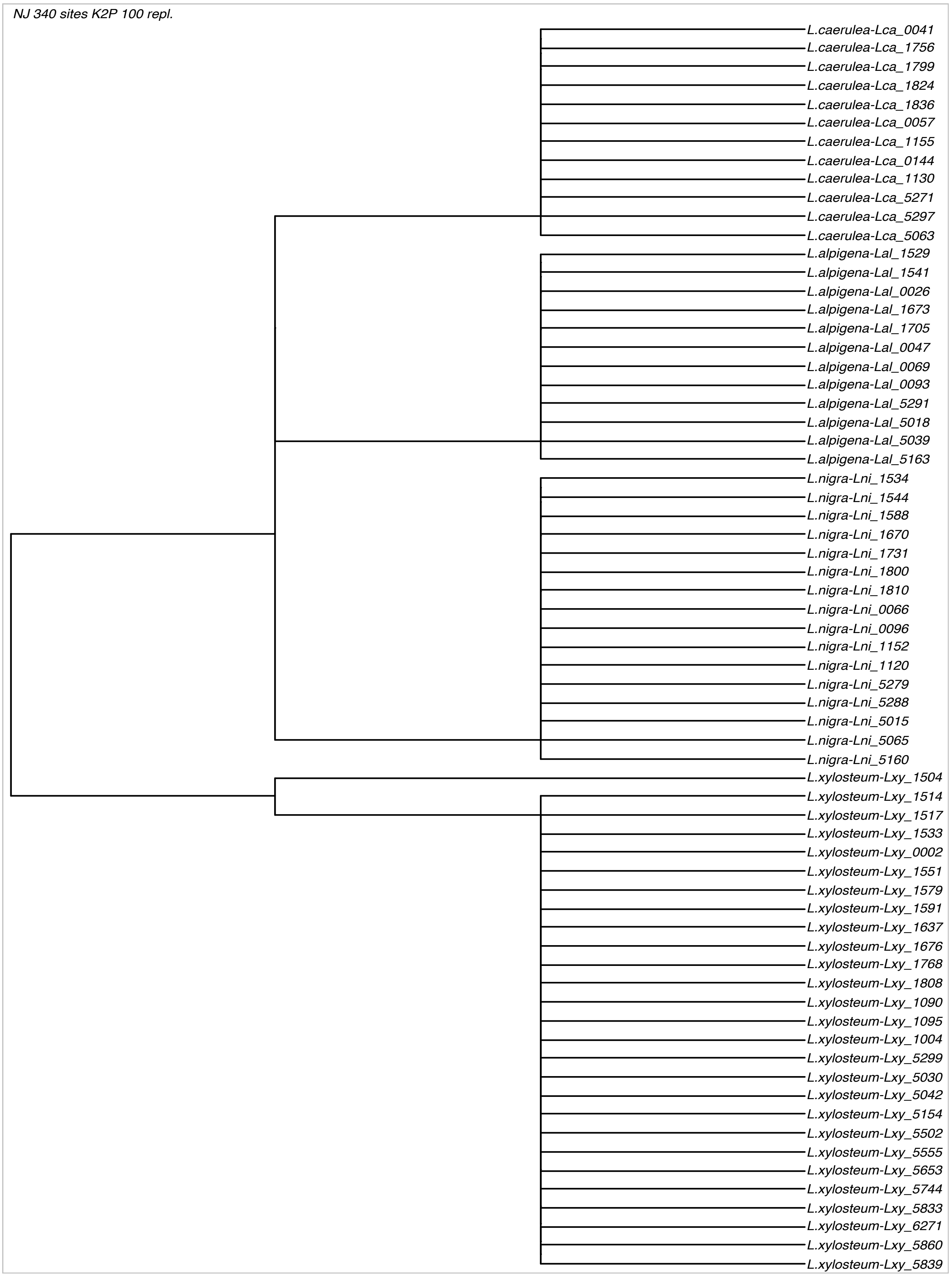

Lonicera
*trn*H-*psb*A

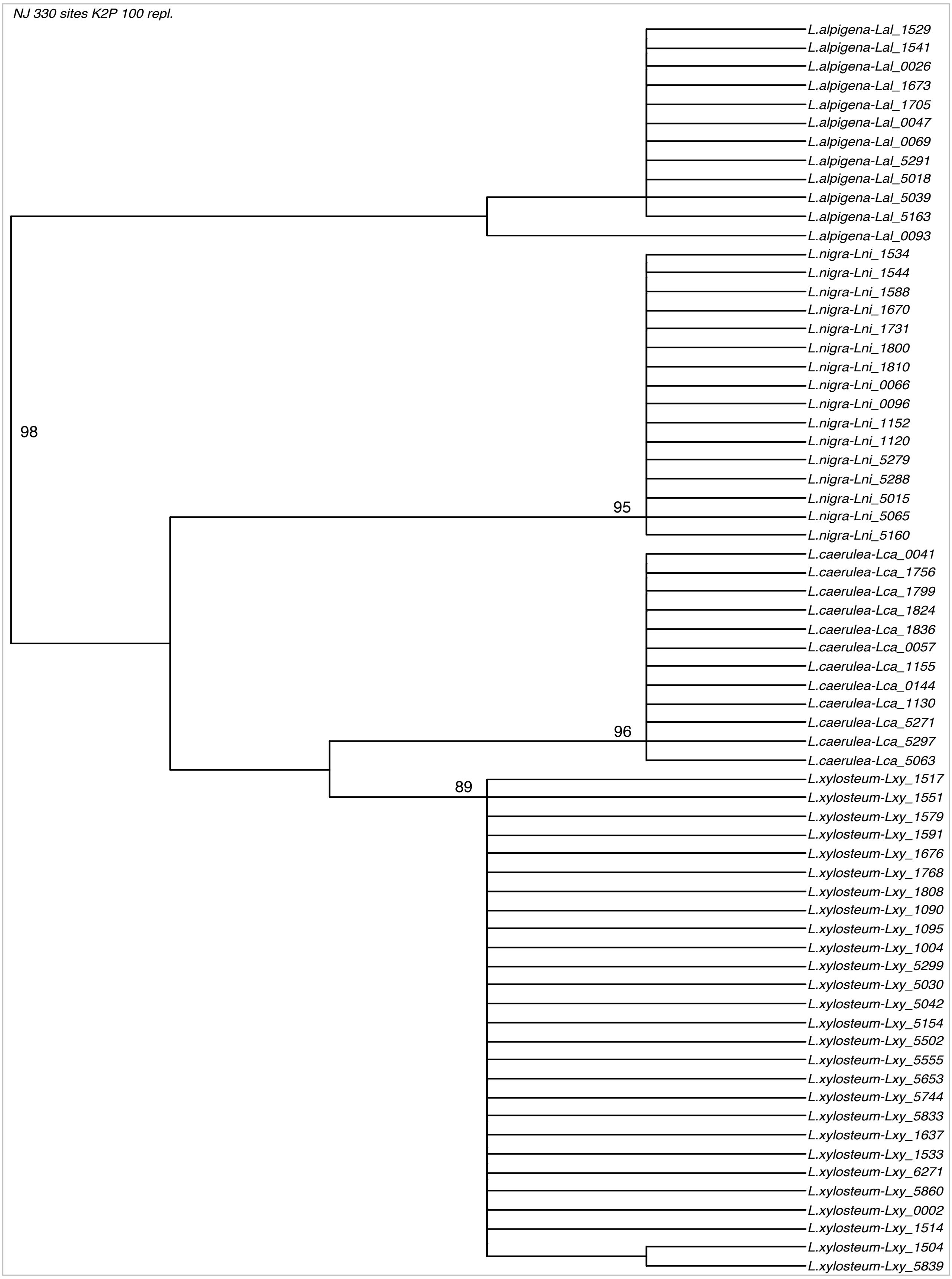

Salix
matK

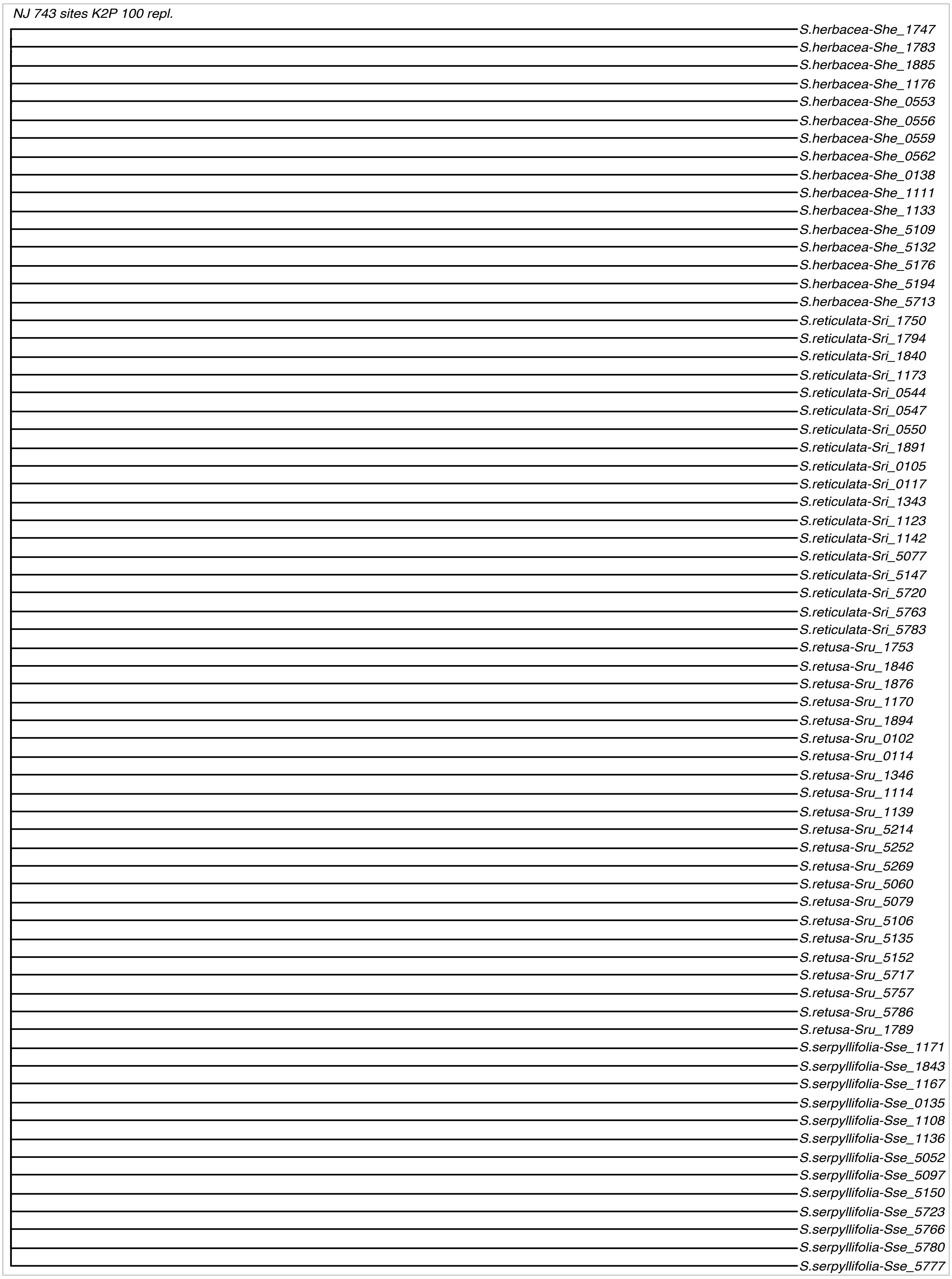
NJ 743 sites K2P 100 repl.
S.herbacea-She_1747
S.herbacea-She_1783
S.herbacea-She_1885
S.herbacea-She_1176
S.herbacea-She_0553
S.herbacea-She_0556
S.herbacea-She_0559
S.herbacea-She_0562
S.herbacea-She_0138
S.herbacea-She_1111
S.herbacea-She_1133
S.herbacea-She_5109
S.herbacea-She_5132
S.herbacea-She_5176
S.herbacea-She_5194
S.herbacea-She_5713
S.reticulata-Sri_1750
S.reticulata-Sri_1794
S.reticulata-Sri_1840
S.reticulata-Sri_1173
S.reticulata-Sri_0544
S.reticulata-Sri_0547
S.reticulata-Sri_0550
S.reticulata-Sri_1891
S.reticulata-Sri_0105
S.reticulata-Sri_0117
S.reticulata-Sri_1343
S.reticulata-Sri_1123
S.reticulata-Sri_1142
S.reticulata-Sri_5077
S.reticulata-Sri_5147
S.reticulata-Sri_5720
S.reticulata-Sri_5763
S.reticulata-Sri_5783
S.retusa-Sru_1753
S.retusa-Sru_1846
S.retusa-Sru_1876
S.retusa-Sru_1170
S.retusa-Sru_1894
S.retusa-Sru_0102
S.retusa-Sru_0114
S.retusa-Sru_1346
S.retusa-Sru_1114
S.retusa-Sru_1139
S.retusa-Sru_5214
S.retusa-Sru_5252
S.retusa-Sru_5269
S.retusa-Sru_5060
S.retusa-Sru_5079
S.retusa-Sru_5106
S.retusa-Sru_5135
S.retusa-Sru_5152
S.retusa-Sru_5717
S.retusa-Sru_5757
S.retusa-Sru_5786
S.retusa-Sru_1789
S.serpyllifolia-Sse_1171
S.serpyllifolia-Sse_1843
S.serpyllifolia-Sse_1167
S.serpyllifolia-Sse_0135
S.serpyllifolia-Sse_1108
S.serpyllifolia-Sse_1136
S.serpyllifolia-Sse_5052
S.serpyllifolia-Sse_5097
S.serpyllifolia-Sse_5150
S.serpyllifolia-Sse_5723
S.serpyllifolia-Sse_5766
S.serpyllifolia-Sse_5780
S.serpyllifolia-Sse_5777

Salix
*rpo*C1

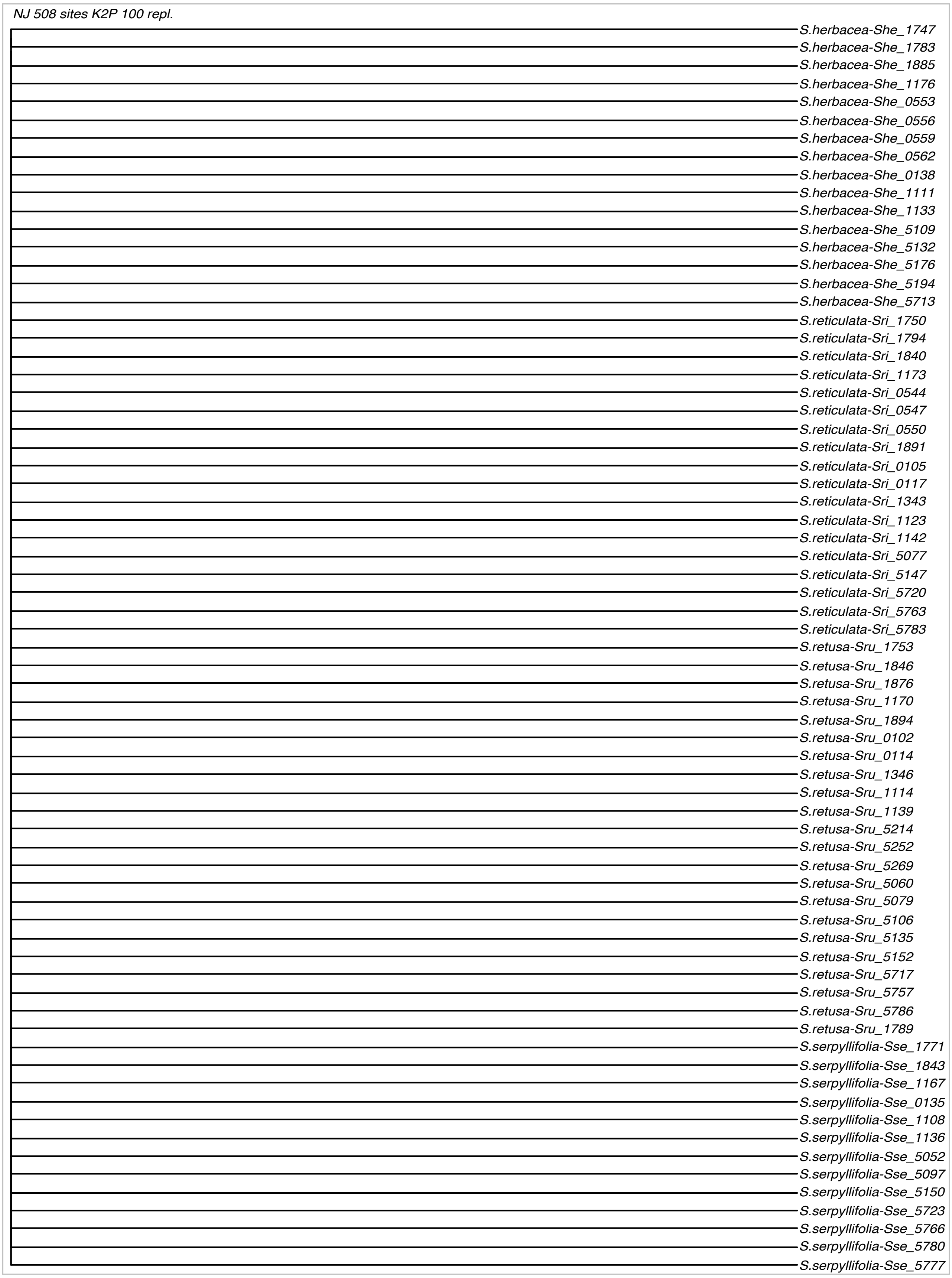
NJ 508 sites K2P 100 repl.
S.herbacea-She_1747
S.herbacea-She_1783
S.herbacea-She_1885
S.herbacea-She_1176
S.herbacea-She_0553
S.herbacea-She_0556
S.herbacea-She_0559
S.herbacea-She_0562
S.herbacea-She_0138
S.herbacea-She_1111
S.herbacea-She_1133
S.herbacea-She_5109
S.herbacea-She_5132
S.herbacea-She_5176
S.herbacea-She_5194
S.herbacea-She_5713
S.reticulata-Sri_1750
S.reticulata-Sri_1794
S.reticulata-Sri_1840
S.reticulata-Sri_1173
S.reticulata-Sri_0544
S.reticulata-Sri_0547
S.reticulata-Sri_0550
S.reticulata-Sri_1891
S.reticulata-Sri_0105
S.reticulata-Sri_0117
S.reticulata-Sri_1343
S.reticulata-Sri_1123
S.reticulata-Sri_1142
S.reticulata-Sri_5077
S.reticulata-Sri_5147
S.reticulata-Sri_5720
S.reticulata-Sri_5763
S.reticulata-Sri_5783
S.retusa-Sru_1753
S.retusa-Sru_1846
S.retusa-Sru_1876
S.retusa-Sru_1170
S.retusa-Sru_1894
S.retusa-Sru_0102
S.retusa-Sru_0114
S.retusa-Sru_1346
S.retusa-Sru_1114
S.retusa-Sru_1139
S.retusa-Sru_5214
S.retusa-Sru_5252
S.retusa-Sru_5269
S.retusa-Sru_5060
S.retusa-Sru_5079
S.retusa-Sru_5106
S.retusa-Sru_5135
S.retusa-Sru_5152
S.retusa-Sru_5717
S.retusa-Sru_5757
S.retusa-Sru_5786
S.retusa-Sru_1789
S.serpyllifolia-Sse_1771
S.serpyllifolia-Sse_1843
S.serpyllifolia-Sse_1167
S.serpyllifolia-Sse_0135
S.serpyllifolia-Sse_1108
S.serpyllifolia-Sse_1136
S.serpyllifolia-Sse_5052
S.serpyllifolia-Sse_5097
S.serpyllifolia-Sse_5150
S.serpyllifolia-Sse_5723
S.serpyllifolia-Sse_5766
S.serpyllifolia-Sse_5780
S.serpyllifolia-Sse_5777

Salix
*rpo*B

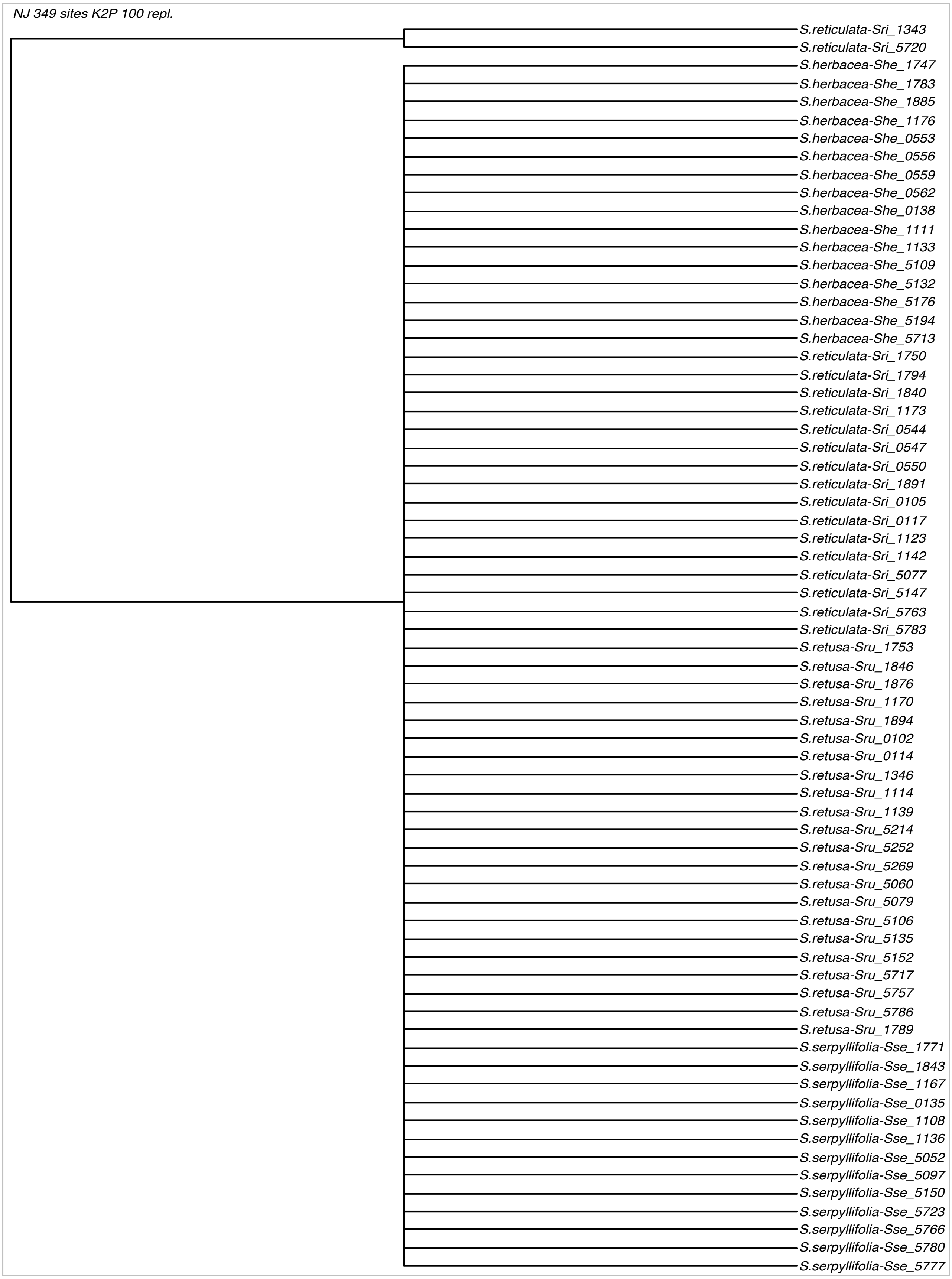
NJ 349 sites K2P 100 repl.
S.reticulata-Sri_1343
S.reticulata-Sri_5720
S.herbacea-She_1747
S.herbacea-She_1783
S.herbacea-She_1885
S.herbacea-She_1176
S.herbacea-She_0553
S.herbacea-She_0556
S.herbacea-She_0559
S.herbacea-She_0562
S.herbacea-She_0138
S.herbacea-She_1111
S.herbacea-She_1133
S.herbacea-She_5109
S.herbacea-She_5132
S.herbacea-She_5176
S.herbacea-She_5194
S.herbacea-She_5713
S.reticulata-Sri_1750
S.reticulata-Sri_1794
S.reticulata-Sri_1840
S.reticulata-Sri_1173
S.reticulata-Sri_0544
S.reticulata-Sri_0547
S.reticulata-Sri_0550
S.reticulata-Sri_1891
S.reticulata-Sri_0105
S.reticulata-Sri_0117
S.reticulata-Sri_1123
S.reticulata-Sri_1142
S.reticulata-Sri_5077
S.reticulata-Sri_5147
S.reticulata-Sri_5763
S.reticulata-Sri_5783
S.retusa-Sru_1753
S.retusa-Sru_1846
S.retusa-Sru_1876
S.retusa-Sru_1170
S.retusa-Sru_1894
S.retusa-Sru_0102
S.retusa-Sru_0114
S.retusa-Sru_1346
S.retusa-Sru_1114
S.retusa-Sru_1139
S.retusa-Sru_5214
S.retusa-Sru_5252
S.retusa-Sru_5269
S.retusa-Sru_5060
S.retusa-Sru_5079
S.retusa-Sru_5106
S.retusa-Sru_5135
S.retusa-Sru_5152
S.retusa-Sru_5717
S.retusa-Sru_5757
S.retusa-Sru_5786
S.retusa-Sru_1789
S.serpyllifolia-Sse_1771
S.serpyllifolia-Sse_1843
S.serpyllifolia-Sse_1167
S.serpyllifolia-Sse_0135
S.serpyllifolia-Sse_1108
S.serpyllifolia-Sse_1136
S.serpyllifolia-Sse_5052
S.serpyllifolia-Sse_5097
S.serpyllifolia-Sse_5150
S.serpyllifolia-Sse_5723
S.serpyllifolia-Sse_5766
S.serpyllifolia-Sse_5780
S.serpyllifolia-Sse_5777

Salix
*trn*H-*psb*A

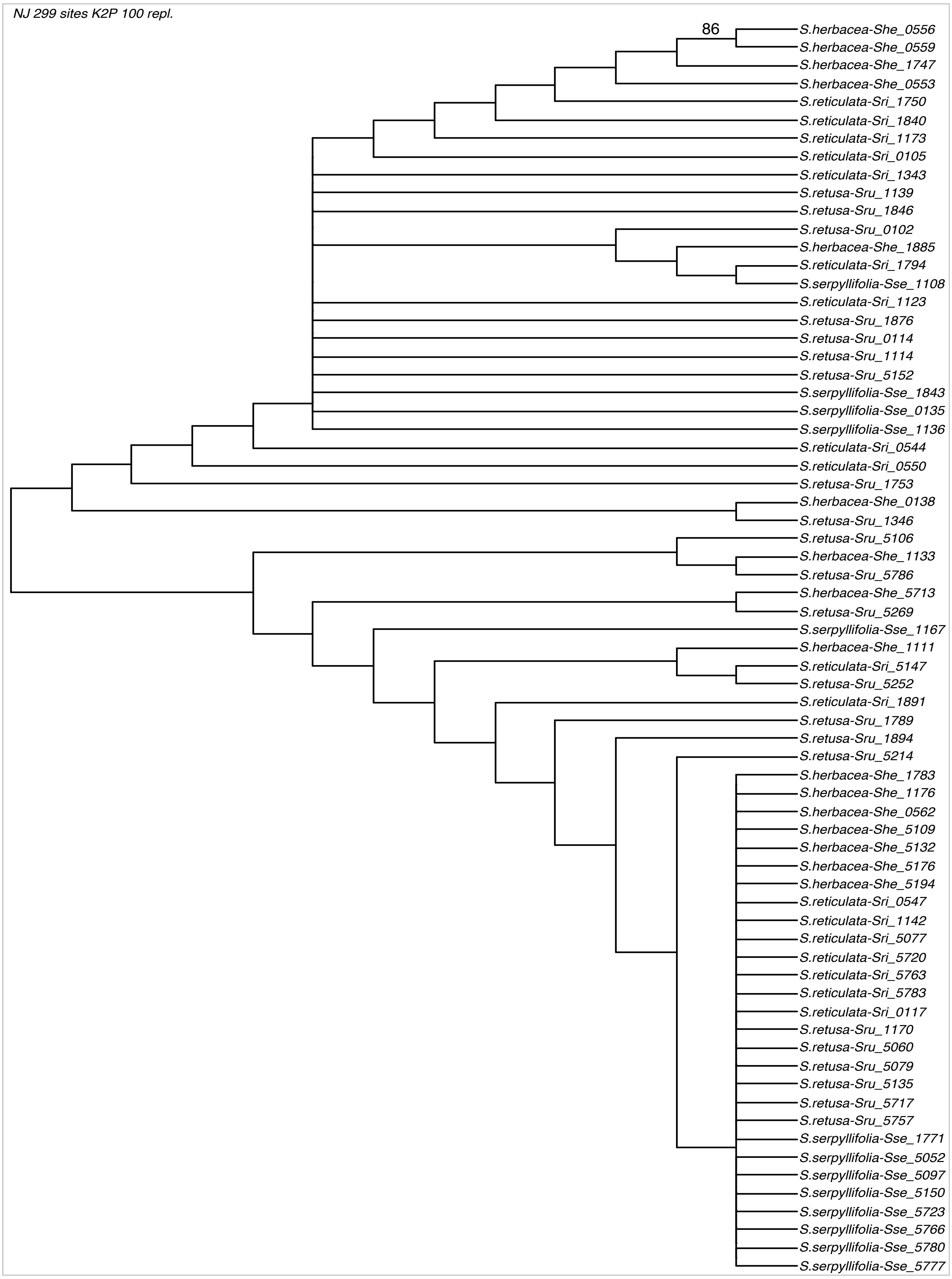

Veronica
*mat*K

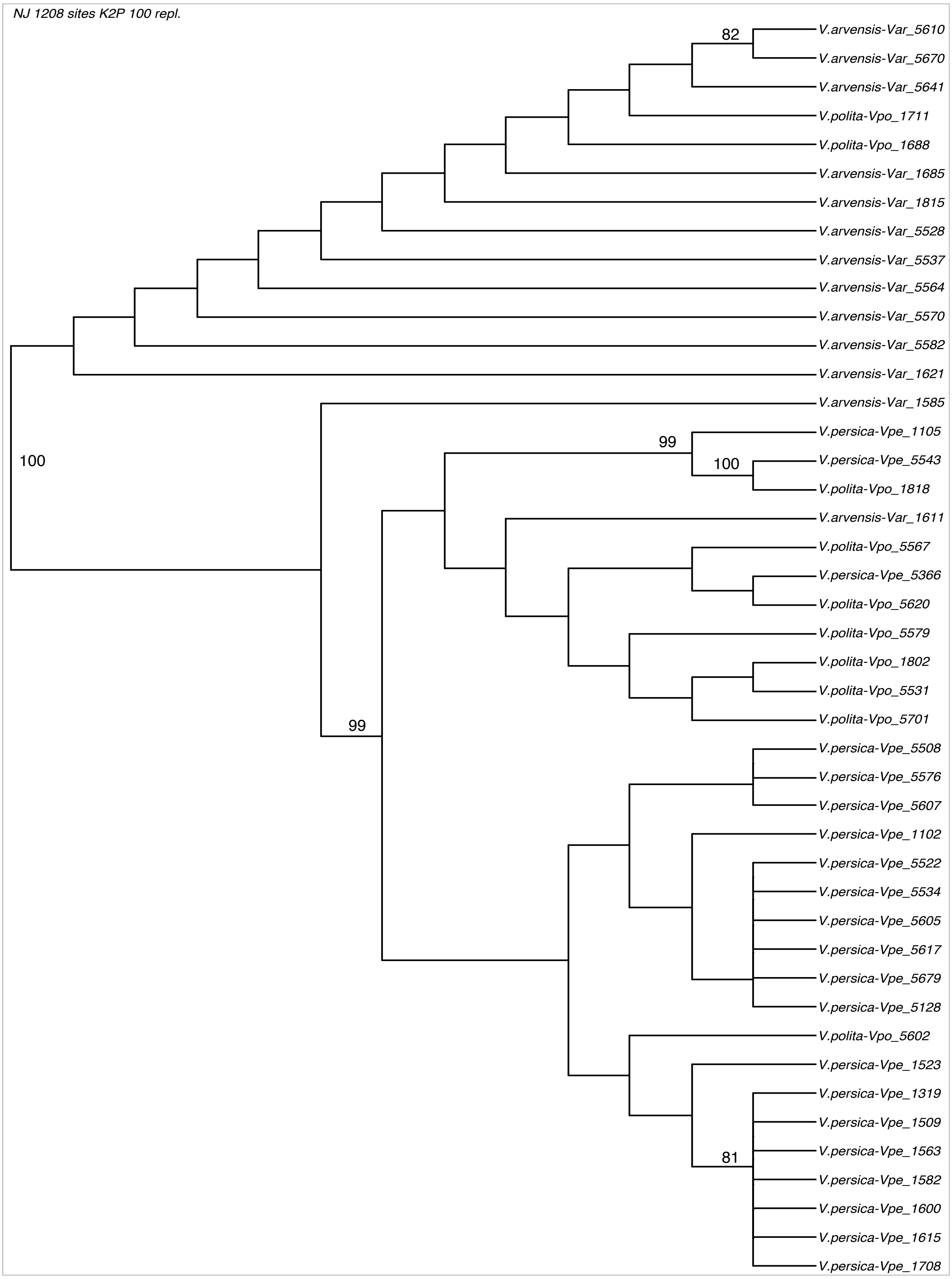

Veronica
*rpo*C1

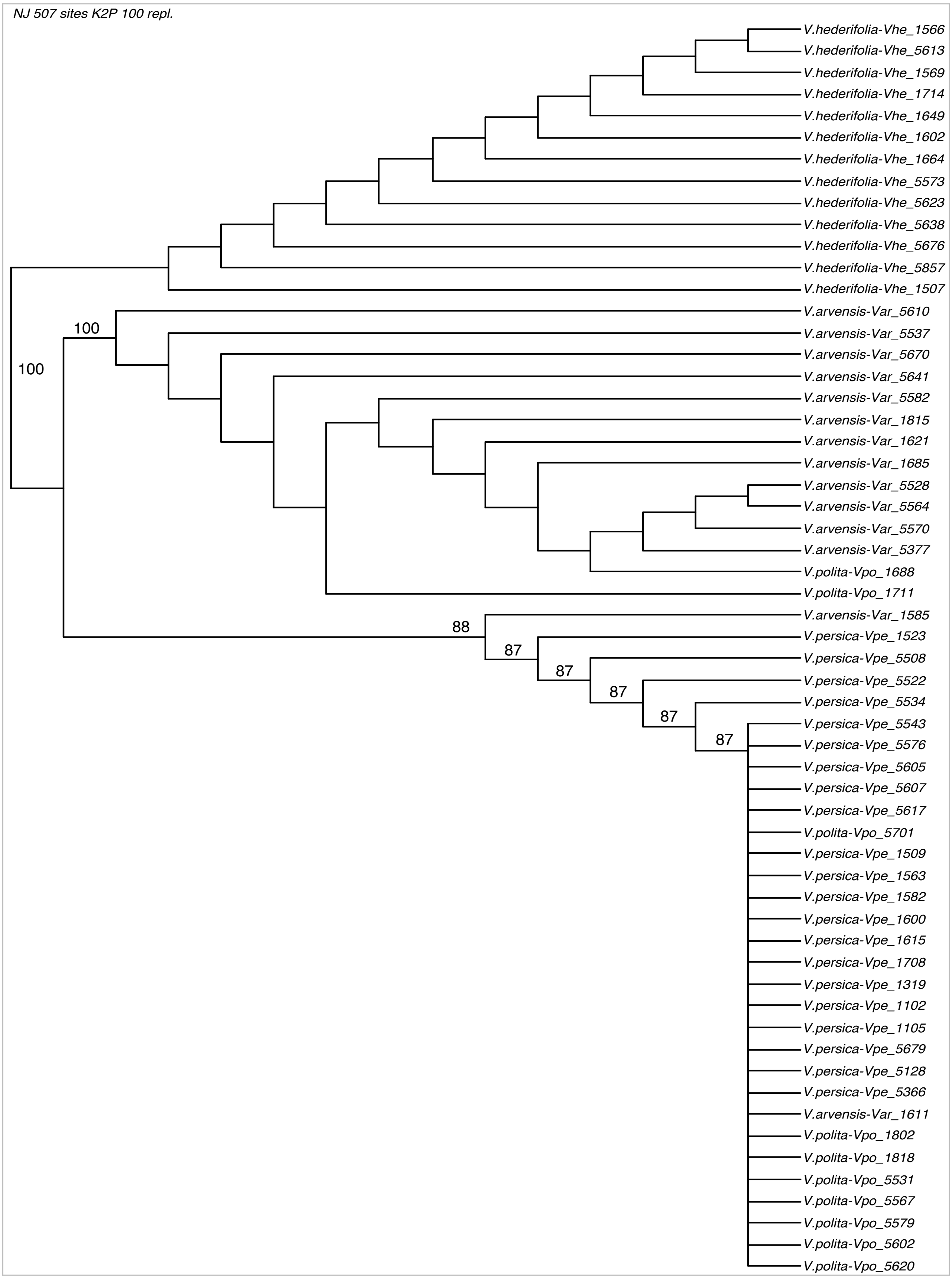

Veronica
*rpo*B

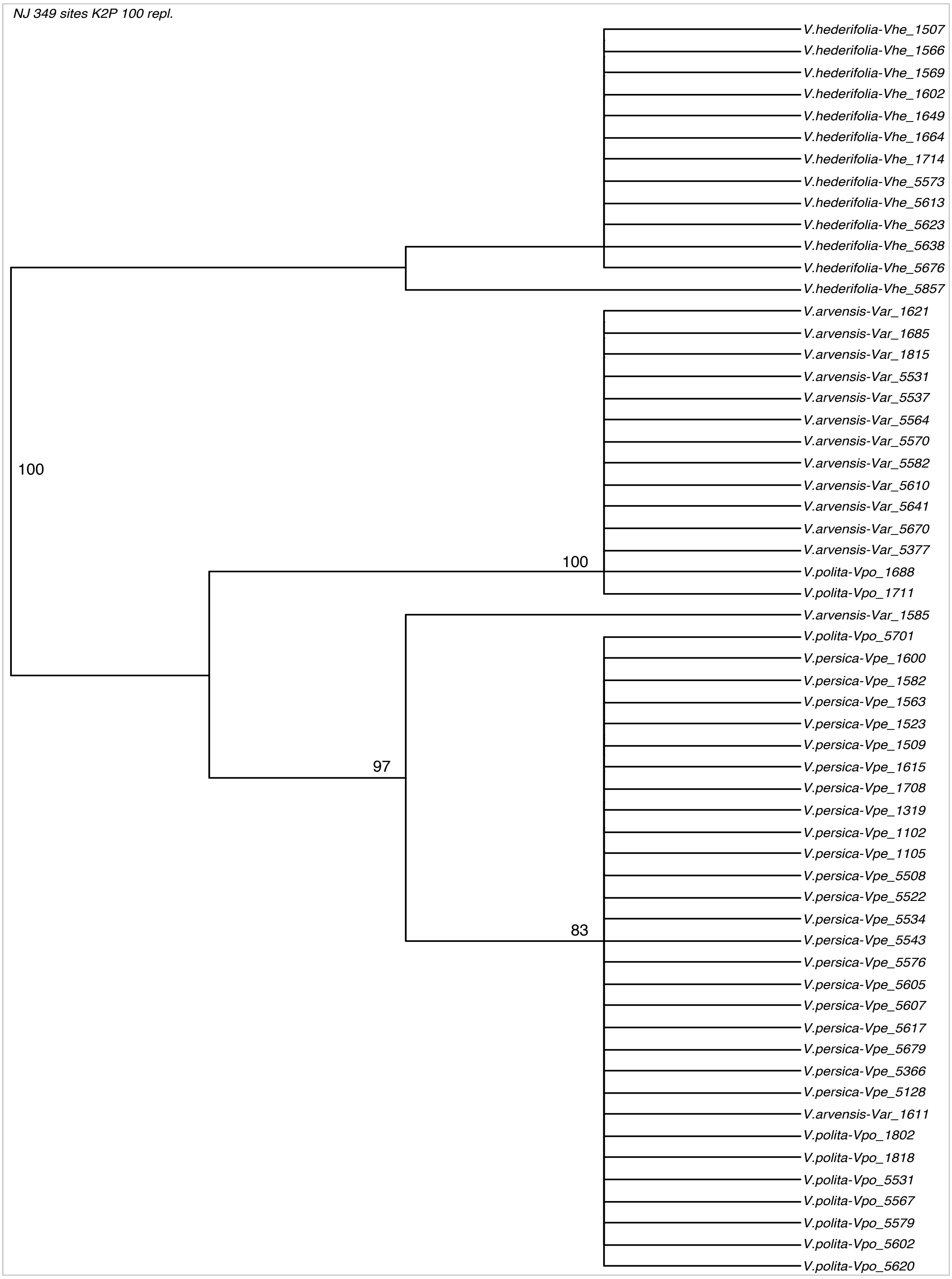

Veronica
*trn*H-*psb*A

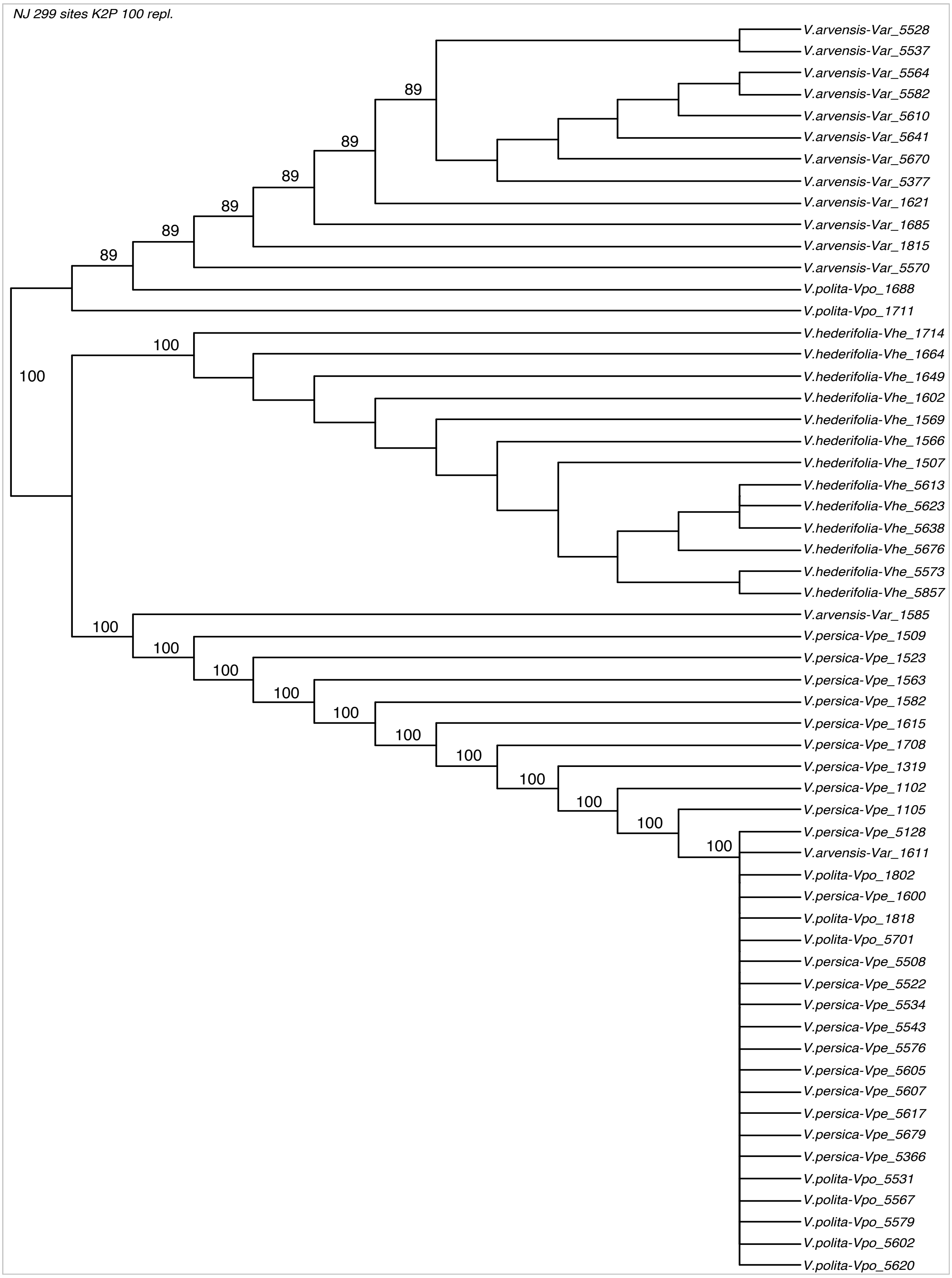

Supplement: Additional file 3: — Phylogenetic trees. For each genus and locus, a neighbour joining tree is presented. Bootstrap values above 80 % are shown above branches. Codes following species names are individual numbers (see Additional file 1). (PDF 572 kb) [file 12862_2016_678_MOESM3_ESM.pdf]
