# Supplementary material for: Evolutionary histories determine DNA barcoding success in vascular plants: seven case studies using intraspecific broad sampling of closely related species
Source: BMC Evol Biol. 2016 May 13;16:103. doi: 10.1186/s12862-016-0678-0 (PMC4866073; doi:10.1186/s12862-016-0678-0)
Supplement: Additional file 4: — Genbank accession numbers. For each sample, accession numbers for the four loci are given. (PDF 71 kb) [file 12862_2016_678_MOESM4_ESM.pdf]

Species	Individual	matK	rpoC1	rpoB	HA
Acer campestre	Aca_1503	KU549935	KU549832	KU549729	KU672731
Acer campestre	Aca_1522	KU549936	KU549833	KU549730	KU672732
Acer campestre	Aca_1553	KU549937	KU549834	KU549731	KU672733
Acer campestre	Aca_0033	KU549938	KU549835	KU549732	KU672734
Acer campestre	Aca_1577	KU549939	KU549836	KU549733	KU672735
Acer campestre	Aca_1593	KU549940	KU549837	KU549734	KU672736
Acer campestre	Aca_1627	KU549941	KU549838	KU549735	KU672737
Acer campestre	Aca_1658	KU549942	KU549839	KU549736	KU672738
Acer campestre	Aca_1697	KU549943	KU549840	KU549737	KU672739
Acer campestre	Aca_1762	KU549944	KU549841	KU549738	KU672740
Acer campestre	Aca_1088	KU549945	KU549842	KU549739	KU672741
Acer campestre	Aca_1328	KU549946	KU549843	KU549740	KU672742
Acer campestre	Aca_1005	KU549947	KU549844	KU549741	KU672743
Acer campestre	Aca_5515	KU549948	KU549845	KU549742	KU672744
Acer campestre	Aca_5525	KU549949	KU549846	KU549743	KU672745
Acer campestre	Aca_5552	KU549950	KU549847	KU549744	KU672746
Acer campestre	Aca_5558	KU549951	KU549848	KU549745	KU672747
Acer campestre	Aca_5588	KU549952	KU549849	KU549746	KU672748
Acer campestre	Aca_5626	KU549953	KU549850	KU549747	KU672749
Acer campestre	Aca_5647	KU549954	KU549851	KU549748	KU672750
Acer campestre	Aca_5659	KU549955	KU549852	KU549749	KU672751
Acer campestre	Aca_5821	KU549956	KU549853	KU549750	KU672752
Acer campestre	Aca_5824	KU549957	KU549854	KU549751	KU672753
Acer campestre	Aca_5836	KU549958	KU549855	KU549752	KU672754
Acer campestre	Aca_1038	KU549959	KU549856	KU549753	KU672755
Acer campestre	Aca_1025	KU549960	KU549857	KU549754	KU672756
Acer campestre	Aca_1031	KU549961	KU549858	KU549755	KU672757
Acer platanoides	Apl_1560	KU549962	KU549859	KU549756	KU672758
Acer platanoides	Apl_1575	KU549963	KU549860	KU549757	KU672759
Acer platanoides	Apl_1607	KU549964	KU549861	KU549758	KU672760
Acer platanoides	Apl_1631	KU549965	KU549862	KU549759	KU672761
Acer platanoides	Apl_1679	KU549966	KU549863	KU549760	KU672762
Acer platanoides	Apl_1870	KU549967	KU549864	KU549761	KU672763
Acer platanoides	Apl_1098	KU549968	KU549865	KU549762	KU672764
Acer platanoides	Apl_1006	KU549969	KU549866	KU549763	KU672765
Acer platanoides	Apl_5088	KU549970	KU549867	KU549764	KU672766
Acer platanoides	Apl_5518	KU549971	KU549868	KU549765	KU672767
Acer platanoides	Apl_5597	KU549972	KU549869	KU549766	KU672768
Acer platanoides	Apl_5822	KU549973	KU549870	KU549767	KU672769
Acer platanoides	Apl_5830	KU549974	KU549871	KU549768	KU672770
Acer platanoides	Apl_1036	KU549975	KU549872	KU549769	KU672771
Acer platanoides	Apl_6265	KU549976	KU549873	KU549770	KU672772
Acer monspessulanum	Amo_1520	KU549977	KU549874	KU549771	KU672773
Acer monspessulanum	Amo_0168	KU549978	KU549875	KU549772	KU672774
Acer monspessulanum	Amo_5546	KU549979	KU549876	KU549773	KU672775
Acer monspessulanum	Amo_5585	KU549980	KU549877	KU549774	KU672776
Acer monspessulanum	Amo_5595	KU549981	KU549878	KU549775	KU672777
Acer monspessulanum	Amo_5629	KU549982	KU549879	KU549776	KU672778
Acer monspessulanum	Amo_5644	KU549983	KU549880	KU549777	KU672779
Acer monspessulanum	Amo_5656	KU549984	KU549881	KU549778	KU672780
Acer monspessulanum	Amo_5667	KU549985	KU549882	KU549779	KU672781
Acer monspessulanum	Amo_6102	KU549986	KU549883	KU549780	KU672782
Acer monspessulanum	Amo_6105	KU549987	KU549884	KU549781	KU672783
Acer opalus	Aop_1597	KU549988	KU549885	KU549782	KU672784
Acer opalus	Aop_1624	KU549989	KU549886	KU549783	KU672785
Acer opalus	Aop_1629	KU549990	KU549887	KU549784	KU672786
Acer opalus	Aop_1655	KU549991	KU549888	KU549785	KU672787
Acer opalus	Aop_1694	KU549992	KU549889	KU549786	KU672788
Acer opalus	Aop_1765	KU549993	KU549890	KU549787	KU672789
Acer opalus	Aop_5208	KU549994	KU549891	KU549788	KU672790
Acer opalus	Aop_5157	KU549995	KU549892	KU549789	KU672791

Acer opalus	Aop_5512	KU549996	KU549893	KU549790	KU672792
Acer opalus	Aop_5632	KU549997	KU549894	KU549791	KU672793
Acer opalus	Aop_5650	KU549998	KU549895	KU549792	KU672794
Acer opalus	Aop_5661	KU549999	KU549896	KU549793	KU672795
Acer opalus	Aop_5549	KU550000	KU549897	KU549794	KU672796
Acer opalus	Aop_1028	-	KU549898	KU549795	KU672797
Acer pseudoplatanus	Aps_1557	KU550001	KU549899	KU549796	KU672798
Acer pseudoplatanus	Aps_1572	KU550002	KU549900	KU549797	KU672799
Acer pseudoplatanus	Aps_1605	KU550003	KU549901	KU549798	KU672800
Acer pseudoplatanus	Aps_1634	KU550004	KU549902	KU549799	KU672801
Acer pseudoplatanus	Aps_1682	KU550005	KU549903	KU549800	KU672802
Acer pseudoplatanus	Aps_1759	KU550006	KU549904	KU549801	KU672803
Acer pseudoplatanus	Aps_1805	KU550007	KU549905	KU549802	KU672804
Acer pseudoplatanus	Aps_1853	KU550008	KU549906	KU549803	KU672805
Acer pseudoplatanus	Aps_1865	KU550009	KU549907	KU549804	KU672806
Acer pseudoplatanus	Aps_1866	KU550010	KU549908	KU549805	KU672807
Acer pseudoplatanus	Aps_0502	KU550011	KU549909	KU549806	KU672808
Acer pseudoplatanus	Aps_0509	KU550012	KU549910	KU549807	KU672809
Acer pseudoplatanus	Aps_1085	KU550013	KU549911	KU549808	KU672810
Acer pseudoplatanus	Aps_1092	KU550014	KU549912	KU549809	KU672811
Acer pseudoplatanus	Aps_1316	KU550015	KU549913	KU549810	KU672812
Acer pseudoplatanus	Aps_1022	KU550016	KU549914	KU549811	KU672813
Acer pseudoplatanus	Aps_1008	KU550017	KU549915	KU549812	KU672814
Acer pseudoplatanus	Aps_0141	KU550018	KU549916	KU549813	KU672815
Acer pseudoplatanus	Aps_1125	KU550019	KU549917	KU549814	KU672816
Acer pseudoplatanus	Aps_5277	KU550020	KU549918	KU549815	KU672817
Acer pseudoplatanus	Aps_5282	KU550021	KU549919	KU549816	KU672818
Acer pseudoplatanus	Aps_5294	KU550022	KU549920	KU549817	KU672819
Acer pseudoplatanus	Aps_5021	KU550023	KU549921	KU549818	KU672820
Acer pseudoplatanus	Aps_5036	KU550024	KU549922	KU549819	KU672821
Acer pseudoplatanus	Aps_5085	KU550025	KU549923	KU549820	KU672822
Acer pseudoplatanus	Aps_5188	-	KU549924	KU549821	KU672823
Acer pseudoplatanus	Aps_5520	KU550026	KU549925	KU549822	KU672824
Acer pseudoplatanus	Aps_5664	KU550027	KU549926	KU549823	KU672825
Acer pseudoplatanus	Aps_5750	-	KU549927	KU549824	KU672826
Acer pseudoplatanus	Aps_5789	KU550028	KU549928	KU549825	KU672827
Acer pseudoplatanus	Aps_5827	KU550029	KU549929	KU549826	KU672828
Acer pseudoplatanus	Aps_1037	KU550030	KU549930	KU549827	KU672829
Acer pseudoplatanus	Aps_1042	-	KU549931	KU549828	KU672830
Acer pseudoplatanus	Aps_6269	KU550031	KU549932	KU549829	KU672831
Acer pseudoplatanus	Aps_1128	KU550032	KU549933	KU549830	KU672832
Acer pseudoplatanus	Aps_0801	-	KU549934	KU549831	KU672833
Salix herbacea	She_1747	KU673302	KU673440	KU673371	KU673509
Salix herbacea	She_1783	KU673303	KU673441	KU673372	KU673510
Salix herbacea	She_1885	KU673304	KU673442	KU673373	KU673511
Salix herbacea	She_1176	KU673305	KU673443	KU673374	KU673512
Salix herbacea	She_0553	KU673306	KU673444	KU673375	KU673513
Salix herbacea	She_0556	KU673307	KU673445	KU673376	KU673514
Salix herbacea	She_0559	KU673308	KU673446	KU673377	KU673515
Salix herbacea	She_0562	KU673309	KU673447	KU673378	KU673516
Salix herbacea	She_0138	KU673310	KU673448	KU673379	KU673517
Salix herbacea	She_1111	KU673311	KU673449	KU673380	KU673518
Salix herbacea	She_1133	KU673312	KU673450	KU673381	KU673519
Salix herbacea	She_5109	KU673313	KU673451	KU673382	KU673520
Salix herbacea	She_5132	KU673314	KU673452	KU673383	KU673521
Salix herbacea	She_5176	KU673315	KU673453	KU673384	KU673565
Salix herbacea	She_5194	KU673316	KU673454	KU673385	KU673522
Salix herbacea	She_5713	KU673317	KU673455	KU673386	KU673523
Salix reticulata	Sri_1750	KU673318	KU673456	KU673387	KU673524
Salix reticulata	Sri_1794	KU673319	KU673457	KU673388	KU673525
Salix reticulata	Sri_1840	KU673320	KU673458	KU673389	KU673526
Salix reticulata	Sri_1173	KU673321	KU673459	KU673390	KU673527
Salix reticulata	Sri_0544	KU673322	KU673460	KU673391	KU673528

Salix reticulata	Sri_0547	KU673323	KU673461	KU673392	KU673529
Salix reticulata	Sri_0550	KU673324	KU673462	KU673393	KU673530
Salix reticulata	Sri_1891	KU673325	KU673463	KU673394	KU673531
Salix reticulata	Sri_0105	KU673326	KU673464	KU673395	KU673532
Salix reticulata	Sri_0117	KU673327	KU673465	KU673396	KU673533
Salix reticulata	Sri_1343	KU673328	KU673466	KU673397	KU673566
Salix reticulata	Sri_1123	KU673329	KU673467	KU673398	KU673534
Salix reticulata	Sri_1142	KU673330	KU673468	KU673399	KU673535
Salix reticulata	Sri_5077	KU673331	KU673469	KU673400	KU673536
Salix reticulata	Sri_5147	KU673332	KU673470	KU673401	KU673537
Salix reticulata	Sri_5720	KU673333	KU673471	KU673402	KU673538
Salix reticulata	Sri_5763	KU673334	KU673472	KU673403	KU673539
Salix reticulata	Sri_5783	KU673335	KU673473	KU673404	KU673567
Salix retusa	Sru_1753	KU673336	KU673474	KU673405	KU673540
Salix retusa	Sru_1846	KU673337	KU673475	KU673406	KU673568
Salix retusa	Sru_1876	KU673338	KU673476	KU673407	KU673569
Salix retusa	Sru_1170	KU673339	KU673477	KU673408	KU673541
Salix retusa	Sru_1894	KU673340	KU673478	KU673409	KU673542
Salix retusa	Sru_0102	KU673341	KU673479	KU673410	KU673570
Salix retusa	Sru_0114	KU673342	KU673480	KU673411	KU673571
Salix retusa	Sru_1346	KU673343	KU673481	KU673412	KU673543
Salix retusa	Sru_1114	KU673344	KU673482	KU673413	KU673572
Salix retusa	Sru_1139	KU673345	KU673483	KU673414	KU673544
Salix retusa	Sru_5214	KU673346	KU673484	KU673415	KU673545
Salix retusa	Sru_5252	KU673347	KU673485	KU673416	KU673573
Salix retusa	Sru_5269	KU673348	KU673486	KU673417	KU673546
Salix retusa	Sru_5060	KU673349	KU673487	KU673418	KU673547
Salix retusa	Sru_5079	KU673350	KU673488	KU673419	KU673574
Salix retusa	Sru_5106	KU673351	KU673489	KU673420	KU673548
Salix retusa	Sru_5135	KU673352	KU673490	KU673421	KU673549
Salix retusa	Sru_5152	KU673353	KU673491	KU673422	KU673550
Salix retusa	Sru_5717	KU673354	KU673492	KU673423	KU673551
Salix retusa	Sru_5757	KU673355	KU673493	KU673424	KU673575
Salix retusa	Sru_5786	KU673356	KU673494	KU673425	KU673576
Salix retusa	Sru_1789	KU673357	KU673495	KU673426	KU673552
Salix serpyllifolia	Sse_1771	KU673358	KU673496	KU673427	KU673553
Salix serpyllifolia	Sse_1843	KU673359	KU673497	KU673428	KU673554
Salix serpyllifolia	Sse_1167	KU673360	KU673498	KU673429	KU673555
Salix serpyllifolia	Sse_0135	KU673361	KU673499	KU673430	KU673556
Salix serpyllifolia	Sse_1108	KU673362	KU673500	KU673431	KU673557
Salix serpyllifolia	Sse_1136	KU673363	KU673501	KU673432	KU673558
Salix serpyllifolia	Sse_5052	KU673364	KU673502	KU673433	KU673577
Salix serpyllifolia	Sse_5097	KU673365	KU673503	KU673434	KU673559
Salix serpyllifolia	Sse_5150	KU673366	KU673504	KU673435	KU673560
Salix serpyllifolia	Sse_5723	KU673367	KU673505	KU673436	KU673561
Salix serpyllifolia	Sse_5766	KU673368	KU673506	KU673437	KU673562
Salix serpyllifolia	Sse_5777	KU673369	KU673507	KU673438	KU673563
Salix serpyllifolia	Sse_5780	KU673370	KU673508	KU673439	KU673564
Adenostyles alliariae	Aal_1797	KU672834	KU672908	KU672871	KU672945
Adenostyles alliariae	Aal_1821	KU672835	KU672909	KU672872	KU672946
Adenostyles alliariae	Aal_1833	KU672836	KU672910	KU672873	KU672947
Adenostyles alliariae	Aal_1882	KU672837	KU672911	KU672874	KU672948
Adenostyles alliariae	Aal_0060	KU672838	KU672912	KU672875	KU672949
Adenostyles alliariae	Aal_1158	KU672839	KU672913	KU672876	KU672950
Adenostyles alliariae	Aal_1082	KU672840	KU672914	KU672877	KU672951
Adenostyles alliariae	Aal_1898	KU672841	KU672915	KU672878	KU672952
Adenostyles alliariae	Aal_5220	KU672842	KU672916	KU672879	KU672953
Adenostyles alliariae	Aal_5274	KU672843	KU672917	KU672880	KU672954
Adenostyles alliariae	Aal_5009	KU672844	KU672918	KU672881	KU672955
Adenostyles alliariae	Aal_5027	KU672845	KU672919	KU672882	KU672956
Adenostyles alliariae	Aal_5045	KU672846	KU672920	KU672883	KU672957
Adenostyles alliariae	Aal_5754	KU672847	KU672921	KU672884	KU672958
Adenostyles glabra	Agl_0020	KU672848	KU672922	KU672885	KU672959

Adenostyles glabra	Agl_1703	KU672849	KU672923	KU672886	KU672960
Adenostyles glabra	Agl_0081	KU672850	KU672924	KU672887	KU672961
Adenostyles glabra	Agl_1164	KU672851	KU672925	KU672888	KU672962
Adenostyles glabra	Agl_0126	KU672852	KU672926	KU672889	KU672963
Adenostyles glabra	Agl_1340	KU672853	KU672927	KU672890	KU672964
Adenostyles glabra	Agl_1117	KU672854	KU672928	KU672891	KU672965
Adenostyles glabra	Agl_1145	KU672855	KU672929	KU672892	KU672966
Adenostyles glabra	Agl_5211	KU672856	KU672930	KU672893	KU672967
Adenostyles glabra	Agl_5012	KU672857	KU672931	KU672894	KU672968
Adenostyles glabra	Agl_5024	KU672858	KU672932	KU672895	KU672969
Adenostyles glabra	Agl_5082	KU672859	KU672933	KU672896	KU672970
Adenostyles glabra	Agl_5166	KU672860	KU672934	KU672897	KU672971
Adenostyles glabra	Agl_5185	KU672861	KU672935	KU672898	KU672972
Adenostyles glabra	Agl_5747	KU672862	KU672936	KU672899	KU672973
Adenostyles glabra	Agl_5706	KU672863	KU672937	KU672900	KU672974
Adenostyles leucophylla	Ale_0123	KU672864	KU672938	KU672901	KU672975
Adenostyles leucophylla	Ale_0132	KU672865	KU672939	KU672902	KU672976
Adenostyles leucophylla	Ale_0150	KU672866	KU672940	KU672903	KU672977
Adenostyles leucophylla	Ale_1148	KU672867	KU672941	KU672904	KU672978
Adenostyles leucophylla	Ale_5122	KU672868	KU672942	KU672905	KU672979
Adenostyles leucophylla	Ale_5179	KU672869	KU672943	KU672906	KU672980
Adenostyles leucophylla	Ale_5774	KU672870	KU672944	KU672907	KU672981
Gentiana angustifolia	Gan_0005	KU673789	KU674047	KU673918	KU674176
Gentiana angustifolia	Gan_0008	KU673790	KU674048	KU673919	KU674177
Gentiana angustifolia	Gan_0011	KU673791	KU674049	KU673920	KU674178
Gentiana angustifolia	Gan_0014	KU673792	KU674050	KU673921	KU674179
Gentiana angustifolia	Gan_0017	KU673793	KU674051	KU673922	KU674180
Gentiana angustifolia	Gan_0023	KF536671	KF536619	KF536645	KF533126
Gentiana angustifolia	Gan_1538	KU673794	KU674052	KU673923	KU674181
Gentiana angustifolia	Gan_1546	KU673795	KU674053	KU673924	KU674182
Gentiana angustifolia	Gan_1548	KU673796	KU674054	KU673925	KU674183
Gentiana angustifolia	Gan_1640	KU673797	KU674055	KU673926	KU674184
Gentiana angustifolia	Gan_1643	KU673798	KU674056	KU673927	KU674185
Gentiana angustifolia	Gan_1646	KU673799	KU674057	KU673928	KU674186
Gentiana angustifolia	Gan_1652	KU673800	KU674058	KU673929	KU674187
Gentiana angustifolia	Gan_1661	KU673801	KU674059	KU673930	KU674188
Gentiana angustifolia	Gan_1667	KU673802	KU674060	KU673931	KU674189
Gentiana angustifolia	Gan_1691	KU673803	KU674061	KU673932	KU674190
Gentiana angustifolia	Gan_1700	KU673804	KU674062	KU673933	KU674191
Gentiana angustifolia	Gan_1720	KU673805	KU674063	KU673934	KU674192
Gentiana angustifolia	Gan_1728	KU673806	KU674064	KU673935	KU674193
Gentiana angustifolia	Gan_0155	KU673807	KU674065	KU673936	KU674194
Gentiana angustifolia	Gan_0158	KU673808	KU674066	KU673937	KU674195
Gentiana angustifolia	Gan_0170	KU673809	KU674067	KU673938	KU674196
Gentiana angustifolia	Gan_5202	KU673810	KU674068	KU673939	KU674197
Gentiana angustifolia	Gan_5205	KU673811	KU674069	KU673940	KU674198
Gentiana angustifolia	Gan_5216	KU673812	KU674070	KU673941	KU674199
Gentiana angustifolia	Gan_5223	KU673813	KU674071	KU673942	KU674200
Gentiana angustifolia	Gan_5225	KU673814	KU674072	KU673943	KU674201
Gentiana angustifolia	Gan_5226	KU673815	KU674073	KU673944	KU674202
Gentiana angustifolia	Gan_5228	KU673816	KU674074	KU673945	KU674203
Gentiana angustifolia	Gan_5302	KU673817	KU674075	KU673946	KU674204
Gentiana angustifolia	Gan_0174	KU673818	KU674076	KU673947	KU674205
Gentiana acaulis	Gac_0029	KU673819	KU674077	KU673948	KU674206
Gentiana acaulis	Gac_1725	KF536684	KF536642	KF536668	KF536698
Gentiana acaulis	Gac_1741	KU673820	KU674078	KU673949	KU674207
Gentiana acaulis	Gac_1744	KU673821	KU674079	KU673950	KU674208
Gentiana acaulis	Gac_1786	KU673822	KU674080	KU673951	KU674209
Gentiana acaulis	Gac_1827	KU673823	KU674081	KU673952	KU674210
Gentiana acaulis	Gac_1830	KU673824	KU674082	KU673953	KU674211
Gentiana acaulis	Gac_1850	KU673825	KU674083	KU673954	KU674212
Gentiana acaulis	Gac_1856	KU673826	KU674084	KU673955	KU674213
Gentiana acaulis	Gac_1879	KU673827	KU674085	KU673956	KU674214

Gentiana acaulis	Gac_0050	KU673828	KU674086	KU673957	KU674215
Gentiana acaulis	Gac_0063	KU673829	KU674087	KU673958	KU674216
Gentiana acaulis	Gac_0072	KU673830	KU674088	KU673959	KU674217
Gentiana acaulis	Gac_0087	KU673831	KU674089	KU673960	KU674218
Gentiana acaulis	Gac_1179	KU673832	KU674090	KU673961	KU674219
Gentiana acaulis	Gac_1896	KU673833	KU674091	KU673962	KU674220
Gentiana acaulis	Gac_1312	KU673834	KU674092	KU673963	KU674221
Gentiana acaulis	Gac_0108	KU673835	KU674093	KU673964	KU674222
Gentiana acaulis	Gac_0129	KU673836	KU674094	KU673965	KU674223
Gentiana acaulis	Gac_0147	KU673837	KU674095	KU673966	KU674224
Gentiana acaulis	Gac_0162	KU673838	KU674096	KU673967	KU674225
Gentiana acaulis	Gac_0177	KU673839	KU674097	KU673968	KU674226
Gentiana acaulis	Gac_5002	KU673840	KU674098	KU673969	KU674227
Gentiana acaulis	Gac_5004	KU673841	KU674099	KU673970	KU674228
Gentiana acaulis	Gac_5033	KU673842	KU674100	KU673971	KU674229
Gentiana acaulis	Gac_5071	KU673843	KU674101	KU673972	KU674230
Gentiana acaulis	Gac_5091	KU673844	KU674102	KU673973	KU674231
Gentiana acaulis	Gac_5100	KU673845	KU674103	KU673974	KU674232
Gentiana acaulis	Gac_5103	KU673846	KU674104	KU673975	KU674233
Gentiana acaulis	Gac_5124	KU673847	KU674105	KU673976	KU674234
Gentiana acaulis	Gac_5141	KU673848	KU674106	KU673977	KU674235
Gentiana acaulis	Gac_5144	KU673849	KU674107	KU673978	KU674236
Gentiana acaulis	Gac_5169	KU673850	KU674108	KU673979	KU674237
Gentiana acaulis	Gac_5182	KU673851	KU674109	KU673980	KU674238
Gentiana acaulis	Gac_5233	KU673852	KU674110	KU673981	KU674239
Gentiana acaulis	Gac_5234	KU673853	KU674111	KU673982	KU674240
Gentiana acaulis	Gac_5255	KU673854	KU674112	KU673983	KU674241
Gentiana acaulis	Gac_5261	KU673855	KU674113	KU673984	KU674242
Gentiana acaulis	Gac_5267	KU673856	KU674114	KU673985	KU674243
Gentiana acaulis	Gac_5709	KU673857	KU674115	KU673986	KU674244
Gentiana acaulis	Gac_5524	KU673858	KU674116	KU673987	KU674245
Gentiana acaulis	Gac_5313	KU673859	KU674117	KU673988	KU674246
Gentiana acaulis	Gac_5318	KU673860	KU674118	KU673989	KU674247
Gentiana acaulis	Gac_0194	KU673861	KU674119	KU673990	KU674248
Gentiana acaulis	Gac_0200	KU673862	KU674120	KU673991	KU674249
Gentiana alpina	Gal_1738	KU673863	KU674121	KU673992	KU674250
Gentiana alpina	Gal_1774	KU673864	KU674122	KU673993	KU674251
Gentiana alpina	Gal_1859	KU673865	KU674123	KU673994	KU674252
Gentiana alpina	Gal_1873	KU673866	KU674124	KU673995	KU674253
Gentiana alpina	Gal_1185	KU673867	KU674125	KU673996	KU674254
Gentiana alpina	Gal_1019	KU673868	KU674126	KU673997	KU674255
Gentiana alpina	Gal_1888	KU673869	KU674127	KU673998	KU674256
Gentiana alpina	Gal_1305	KU673870	KU674128	KU673999	KU674257
Gentiana alpina	Gal_0111	KU673871	KU674129	KU674000	KU674258
Gentiana alpina	Gal_0120	KU673872	KU674130	KU674001	KU674259
Gentiana alpina	Gal_5112	KU673873	KU674131	KU674002	KU674260
Gentiana alpina	Gal_5115	KU673874	KU674132	KU674003	KU674261
Gentiana alpina	Gal_5118	KU673875	KU674133	KU674004	KU674262
Gentiana alpina	Gal_5138	KU673876	KU674134	KU674005	KU674263
Gentiana alpina	Gal_5143	KU673877	KU674135	KU674006	KU674264
Gentiana alpina	Gal_5172	KU673878	KU674136	KU674007	KU674265
Gentiana alpina	Gal_5191	KU673879	KU674137	KU674008	KU674266
Gentiana alpina	Gal_5197	KU673880	KU674138	KU674009	KU674267
Gentiana alpina	Gal_5769	KU673881	KU674139	KU674010	KU674268
Gentiana alpina	Gal_5351	KU673882	-	KU674011	KU674269
Gentiana alpina	Gal_5357	KU673883	KU674140	KU674012	KU674270
Gentiana alpina	Gal_5244	KU673884	KU674141	KU674013	KU674271
Gentiana alpina	Gal_5246	-	KU674142	KU674014	KU674272
Gentiana clusii	Gcl_0044	KU673885	KU674143	KU674015	KU674273
Gentiana clusii	Gcl_1780	KU673886	KU674144	KU674016	KU674274
Gentiana clusii	Gcl_0054	KU673887	KU674145	KU674017	KU674275
Gentiana clusii	Gcl_0075	KU673888	KU674146	KU674018	KU674276
Gentiana clusii	Gcl_0078	KU673889	KU674147	KU674019	KU674277

Gentiana clusii	Gcl_0084	KU673890	KU674148	KU674020	KU674278
Gentiana clusii	Gcl_0090	KU673891	KU674149	KU674021	KU674279
Gentiana clusii	Gcl_1160	KF536688	KF536632	KF536661	KF533123
Gentiana clusii	Gcl_0099	KU673892	KU674150	KU674022	KU674280
Gentiana clusii	Gcl_0153	KU673893	KU674151	KU674023	KU674281
Gentiana clusii	Gcl_1331	KU673894	KU674152	KU674024	KU674282
Gentiana clusii	Gcl_1337	KU673895	KU674153	KU674025	KU674283
Gentiana clusii	Gcl_0165	KU673896	KU674154	KU674026	KU674284
Gentiana clusii	Gcl_5006	KU673897	KU674155	KU674027	KU674285
Gentiana clusii	Gcl_5048	KF536690	KF536634	KF536663	KF533125
Gentiana clusii	Gcl_5049	KU673898	KU674156	KU674028	KU674286
Gentiana clusii	Gcl_5056	KU673899	KU674157	KU674029	KU674287
Gentiana clusii	Gcl_5094	KU673900	KU674158	KU674030	KU674288
Gentiana clusii	Gcl_5068	KU673901	KU674159	KU674031	KU674289
Gentiana clusii	Gcl_5074	KU673902	KU674160	KU674032	KU674290
Gentiana clusii	Gcl_5231	KU673903	KU674161	KU674033	KU674291
Gentiana clusii	Gcl_5258	KU673904	KU674162	KU674034	KU674292
Gentiana clusii	Gcl_5264	KU673905	KU674163	KU674035	KU674293
Gentiana clusii	Gcl_5285	KU673906	KU674164	KU674036	KU674294
Gentiana clusii	Gcl_5236	KU673907	KU674165	KU674037	KU674295
Gentiana clusii	Gcl_5760	KU673908	KU674166	KU674038	KU674296
Gentiana clusii	Gcl_6526	KU673909	KU674167	KU674039	KU674297
Gentiana clusii	Gcl_5315	KU673910	KU674168	KU674040	KU674298
Gentiana clusii	Gcl_0180	KU673911	KU674169	KU674041	KU674299
Gentiana clusii	Gcl_0184	KU673912	KU674170	KU674042	KU674300
Gentiana clusii	Gcl_0187	KU673913	KU674171	KU674043	KU674301
Gentiana clusii	Gcl_0189	KF536693	KF536639	KF536666	KF533133
Gentiana clusii	Gcl_0191	KU673914	KU674172	KU674044	KU674302
Gentiana clusii	Gcl_0198	KU673915	KU674173	KU674045	KU674303
Gentiana clusii	Gcl_0202	KU673916	KU674174	-	KU674304
Gentiana clusii	Gcl_0205	KU673917	KU674175	KU674046	KU674305
Lonicera caerulea	Lca_0041	KU673044	KU673168	KU673101	KU673235
Lonicera caerulea	Lca_1756	KU673045	KU673169	KU673102	KU673236
Lonicera caerulea	Lca_1799	KU673046	KU673170	KU673103	KU673237
Lonicera caerulea	Lca_1824	KU673047	KU673171	KU673104	KU673238
Lonicera caerulea	Lca_1836	KU673048	KU673172	KU673105	KU673239
Lonicera caerulea	Lca_0057	KU673049	KU673173	KU673106	KU673240
Lonicera caerulea	Lca_1155	KU673050	KU673174	KU673107	KU673241
Lonicera caerulea	Lca_0144	KU673051	KU673175	KU673108	KU673242
Lonicera caerulea	Lca_1130	KU673052	KU673176	KU673109	KU673243
Lonicera caerulea	Lca_5271	KU673053	KU673177	KU673110	KU673244
Lonicera caerulea	Lca_5297	-	KU673178	KU673111	KU673245
Lonicera caerulea	Lca_5063	-	KU673179	KU673112	KU673246
Lonicera alpigena	Lal_1529	KU673054	KU673180	KU673113	KU673247
Lonicera alpigena	Lal_1541	KU673055	KU673181	KU673114	KU673248
Lonicera alpigena	Lal_0026	KU673056	KU673182	KU673115	KU673249
Lonicera alpigena	Lal_1673	KU673057	KU673183	KU673116	KU673250
Lonicera alpigena	Lal_1705	KU673058	KU673184	KU673117	KU673251
Lonicera alpigena	Lal_0047	KU673059	KU673185	KU673118	KU673252
Lonicera alpigena	Lal_0069	KU673060	KU673186	KU673119	KU673253
Lonicera alpigena	Lal_0093	KU673061	KU673187	KU673120	KU673254
Lonicera alpigena	Lal_5291	KU673062	KU673188	KU673121	KU673255
Lonicera alpigena	Lal_5018	KU673063	KU673189	KU673122	KU673256
Lonicera alpigena	Lal_5039	-	KU673190	KU673123	KU673257
Lonicera alpigena	Lal_5163	KU673064	KU673191	KU673124	KU673258
Lonicera nigra	Lni_1534	KU673065	KU673192	KU673125	KU673259
Lonicera nigra	Lni_1544	KU673066	KU673193	KU673126	KU673260
Lonicera nigra	Lni_1588	KU673067	KU673194	KU673127	KU673261
Lonicera nigra	Lni_1670	KU673068	KU673195	KU673128	KU673262
Lonicera nigra	Lni_1731	KU673069	KU673196	KU673129	KU673263
Lonicera nigra	Lni_1800	KU673070	KU673197	KU673130	KU673264
Lonicera nigra	Lni_1810	KU673071	KU673198	KU673131	KU673265
Lonicera nigra	Lni_0066	KU673072	KU673199	KU673132	KU673266

Lonicera nigra	Lni_0096	KU673073	KU673200	KU673133	KU673267
Lonicera nigra	Lni_1152	KU673074	KU673201	KU673134	KU673268
Lonicera nigra	Lni_1120	KU673075	KU673202	KU673135	KU673269
Lonicera nigra	Lni_5279	-	KU673203	KU673136	KU673270
Lonicera nigra	Lni_5288	-	KU673204	KU673137	KU673271
Lonicera nigra	Lni_5015	KU673076	KU673205	KU673138	KU673272
Lonicera nigra	Lni_5065	-	KU673206	KU673139	KU673273
Lonicera nigra	Lni_5160	-	KU673207	KU673140	KU673274
Lonicera xylosteum	Lxy_1504	KU673077	KU673208	KU673141	KU673275
Lonicera xylosteum	Lxy_1514	KU673078	KU673209	KU673142	KU673276
Lonicera xylosteum	Lxy_1517	-	KU673210	KU673143	KU673277
Lonicera xylosteum	Lxy_1533	KU673079	KU673211	KU673144	KU673278
Lonicera xylosteum	Lxy_0002	KU673080	KU673212	KU673145	KU673279
Lonicera xylosteum	Lxy_1551	KU673081	KU673213	KU673146	KU673280
Lonicera xylosteum	Lxy_1579	KU673082	KU673214	KU673147	KU673281
Lonicera xylosteum	Lxy_1591	KU673083	KU673215	KU673148	KU673282
Lonicera xylosteum	Lxy_1637	KU673084	KU673216	KU673149	KU673283
Lonicera xylosteum	Lxy_1676	KU673085	KU673217	KU673150	KU673284
Lonicera xylosteum	Lxy_1768	KU673086	KU673218	KU673151	KU673285
Lonicera xylosteum	Lxy_1808	KU673087	KU673219	KU673152	KU673286
Lonicera xylosteum	Lxy_1090	KU673088	KU673220	KU673153	KU673287
Lonicera xylosteum	Lxy_1095	KU673089	KU673221	KU673154	KU673288
Lonicera xylosteum	Lxy_1004	KU673090	KU673222	KU673155	KU673289
Lonicera xylosteum	Lxy_5299	KU673091	KU673223	KU673156	KU673290
Lonicera xylosteum	Lxy_5030	KU673092	KU673224	KU673157	KU673291
Lonicera xylosteum	Lxy_5042	-	KU673225	KU673158	KU673292
Lonicera xylosteum	Lxy_5154	-	KU673226	KU673159	KU673293
Lonicera xylosteum	Lxy_5502	KU673093	KU673227	KU673160	KU673294
Lonicera xylosteum	Lxy_5555	KU673094	KU673228	KU673161	KU673295
Lonicera xylosteum	Lxy_5653	KU673095	KU673229	KU673162	KU673296
Lonicera xylosteum	Lxy_5744	KU673096	KU673230	KU673163	KU673297
Lonicera xylosteum	Lxy_5833	KU673097	KU673231	KU673164	KU673298
Lonicera xylosteum	Lxy_6271	KU673098	KU673232	KU673165	KU673299
Lonicera xylosteum	Lxy_5839	KU673099	KU673233	KU673166	KU673300
Lonicera xylosteum	Lxy_5860	KU673100	KU673234	KU673167	KU673301
Geranium columbinum	Gco_1717	KU672982	KU673012	KU672996	KU673028
Geranium columbinum	Gco_5365	-	KU673013	KU672997	KU673029
Geranium columbinum	Gco_5398	-	KU673014	KU672998	KU673030
Geranium dissectum	Gdi_1613	KU672983	KU673015	KU672999	KU673031
Geranium dissectum	Gdi_0038	KU672984	KU673016	KU673000	KU673032
Geranium dissectum	Gdi_1618	KU672985	KU673017	KU673001	KU673033
Geranium dissectum	Gdi_5540	KU672986	KU673018	KU673002	KU673034
Geranium dissectum	Gdi_5561	KU672987	KU673019	KU673003	KU673035
Geranium dissectum	Gdi_5599	KU672988	KU673020	KU673004	KU673036
Geranium dissectum	Gdi_5615	KU672989	KU673021	KU673005	KU673037
Geranium dissectum	Gdi_5635	KU672990	KU673022	KU673006	KU673038
Geranium dissectum	Gdi_5673	KU672991	KU673023	KU673007	KU673039
Geranium dissectum	Gdi_5395	KU672992	KU673024	KU673008	KU673040
Geranium pusillum	Gpu_1812	KU672993	KU673025	KU673009	KU673041
Geranium pusillum	Gpu_1322	KU672994	KU673026	KU673010	KU673042
Geranium pusillum	Gpu_1325	KU672995	KU673027	KU673011	KU673043
Veronica persica	Vpe_1509	KU673578	KU673673	KU673615	KU673731
Veronica persica	Vpe_1523	KU673579	KU673674	KU673616	KU673732
Veronica persica	Vpe_1563	KU673580	KU673675	KU673617	KU673733
Veronica persica	Vpe_1582	KU673581	KU673676	KU673618	KU673734
Veronica persica	Vpe_1600	KU673582	KU673677	KU673619	KU673735
Veronica persica	Vpe_1615	KU673583	KU673678	KU673620	KU673736
Veronica persica	Vpe_1708	KU673584	KU673679	KU673621	KU673737
Veronica persica	Vpe_1319	KU673585	KU673680	KU673622	KU673738
Veronica persica	Vpe_1102	KU673586	KU673681	KU673623	KU673739
Veronica persica	Vpe_1105	KU673587	KU673682	KU673624	KU673740
Veronica persica	Vpe_5508	KU673588	KU673683	KU673625	KU673741

Veronica persica	Vpe_5522	KU673589	KU673684	KU673626	KU673742
Veronica persica	Vpe_5534	KU673590	KU673685	KU673627	KU673743
Veronica persica	Vpe_5543	KU673591	KU673686	KU673628	KU673744
Veronica persica	Vpe_5576	KU673592	KU673687	KU673629	KU673745
Veronica persica	Vpe_5605	KU673593	KU673688	KU673630	KU673746
Veronica persica	Vpe_5607	KU673594	KU673689	KU673631	KU673747
Veronica persica	Vpe_5617	KU673595	KU673690	KU673632	KU673748
Veronica persica	Vpe_5679	KU673596	KU673691	KU673633	KU673749
Veronica persica	Vpe_5366	KU673597	KU673692	KU673634	KU673750
Veronica persica	Vpe_5128	KU673598	KU673693	KU673635	KU673751
Veronica arvensis	Var_1585	KU673599	KU673694	KU673636	KU673752
Veronica arvensis	Var_1611	KU673600	KU673695	KU673637	KU673753
Veronica arvensis	Var_1621	KU673601	KU673696	KU673638	KU673754
Veronica arvensis	Var_1685	KU673602	KU673697	KU673639	KU673755
Veronica arvensis	Var_1815	KU673603	KU673698	KU673640	KU673756
Veronica arvensis	Var_5528	KU673604	KU673699	KU673641	KU673757
Veronica arvensis	Var_5537	KU673605	KU673700	KU673642	KU673758
Veronica arvensis	Var_5564	KU673606	KU673701	KU673643	KU673759
Veronica arvensis	Var_5570	KU673607	KU673702	KU673644	KU673760
Veronica arvensis	Var_5582	KU673608	KU673703	KU673645	KU673761
Veronica arvensis	Var_5610	-	KU673704	KU673646	KU673762
Veronica arvensis	Var_5641	KU673609	KU673705	KU673647	KU673763
Veronica arvensis	Var_5670	-	KU673706	KU673648	KU673764
Veronica arvensis	Var_5377	-	KU673707	KU673649	KU673765
Veronica polita	Vpo_1688	KU673610	KU673708	KU673650	KU673766
Veronica polita	Vpo_1711	KU673611	KU673709	KU673651	KU673767
Veronica polita	Vpo_1802	KU673612	KU673710	KU673652	KU673768
Veronica polita	Vpo_1818	KU673613	KU673711	KU673653	KU673769
Veronica polita	Vpo_5531	-	KU673712	KU673654	KU673770
Veronica polita	Vpo_5567	-	KU673713	KU673655	KU673771
Veronica polita	Vpo_5579	-	KU673714	KU673656	KU673772
Veronica polita	Vpo_5602	KU673614	KU673715	KU673657	KU673773
Veronica polita	Vpo_5620	-	KU673716	KU673658	KU673774
Veronica polita	Vpo_5701	-	KU673717	KU673659	KU673775
Veronica hederifolia	Vhe_1507	-	KU673718	KU673660	KU673776
Veronica hederifolia	Vhe_1566	-	KU673719	KU673661	KU673777
Veronica hederifolia	Vhe_1569	-	KU673720	KU673662	KU673778
Veronica hederifolia	Vhe_1602	-	KU673721	KU673663	KU673779
Veronica hederifolia	Vhe_1649	-	KU673722	KU673664	KU673780
Veronica hederifolia	Vhe_1664	-	KU673723	KU673665	KU673781
Veronica hederifolia	Vhe_1714	-	KU673724	KU673666	KU673782
Veronica hederifolia	Vhe_5573	-	KU673725	KU673667	KU673783
Veronica hederifolia	Vhe_5613	-	KU673726	KU673668	KU673784
Veronica hederifolia	Vhe_5623	-	KU673727	KU673669	KU673785
Veronica hederifolia	Vhe_5638	-	KU673728	KU673670	KU673786
Veronica hederifolia	Vhe_5676	-	KU673729	KU673671	KU673787
Veronica hederifolia	Vhe_5857	-	KU673730	KU673672	KU673788
